# Protein-Protein Docking with Dynamic Residue Protonation States

**DOI:** 10.1371/journal.pcbi.1004018

**Published:** 2014-12-11

**Authors:** Krishna Praneeth Kilambi, Kavan Reddy, Jeffrey J. Gray

**Affiliations:** 1 Department of Chemical & Biomolecular Engineering, Johns Hopkins University, Baltimore, Maryland, United States of America; 2 Program in Molecular Biophysics, Johns Hopkins University, Baltimore, Maryland, United States of America; University of Houston, United States of America

## Abstract

Protein-protein interactions depend on a host of environmental factors. Local pH conditions influence the interactions through the protonation states of the ionizable residues that can change upon binding. In this work, we present a pH-sensitive docking approach, pHDock, that can sample side-chain protonation states of five ionizable residues (Asp, Glu, His, Tyr, Lys) on-the-fly during the docking simulation. pHDock produces successful local docking funnels in approximately half (79/161) the protein complexes, including 19 cases where standard RosettaDock fails. pHDock also performs better than the two control cases comprising docking at pH 7.0 or using fixed, predetermined protonation states. On average, the top-ranked pHDock structures have lower interface RMSDs and recover more native interface residue-residue contacts and hydrogen bonds compared to RosettaDock. Addition of backbone flexibility using a computationally-generated conformational ensemble further improves native contact and hydrogen bond recovery in the top-ranked structures. Although pHDock is designed to improve docking, it also successfully predicts a large pH-dependent binding affinity change in the Fc–FcRn complex, suggesting that it can be exploited to improve affinity predictions. The approaches in the study contribute to the goal of structural simulations of whole-cell protein-protein interactions including all the environmental factors, and they can be further expanded for pH-sensitive protein design.

## Author Summary

Protein-protein interactions are fundamental for biological function and are strongly influenced by their local environment. Cellular pH is tightly controlled and is one of the critical environmental factors that regulates protein-protein interactions. Three-dimensional structures of the protein complexes can help us understand the mechanism of the interactions. Since experimental determination of the structures of protein-protein complexes is expensive and time-consuming, computational docking algorithms are helpful to predict the structures. However, none of the current protein-protein docking algorithms account for the critical environmental pH effects. So we developed a pH-sensitive docking algorithm that can dynamically pick the favorable protonation states of the ionizable amino-acid residues. Compared to our previous standard docking algorithm, the new algorithm improves docking accuracy and generates higher-quality predictions over a large dataset of protein-protein complexes. We also use a case study to demonstrate efficacy of the algorithm in predicting a large pH-dependent binding affinity change that cannot be captured by the other methods that neglect pH effects. In principle, the approaches in the study can be used for rational design of pH-dependent protein inhibitors or industrial enzymes that are active over a wide range of pH values.

This is a *PLOS Computational Biology* Methods article.

## Introduction

Through tightly controlled cellular pH, posttranslational modification by protons regulates biological function [1]. Cellular pH can vary from highly-acidic in the lysosomes (∼pH 5) to basic in the peroxisomes (∼pH 8) [2], profoundly influencing biomolecular folding and assembly processes [3], [4]. pH effects are especially critical in protein-protein binding, and binding-induced protonation state changes contribute to the association energy of most protein-protein complexes [5], [6]. However, computational protein-protein docking algorithms often ignore the pH effects. In this paper, we develop a pH-sensitive protein-protein docking algorithm and demonstrate that it can improve prediction accuracy and recover pH-dependent binding effects.

Computational docking algorithms are playing an increasingly influential role in driving large-scale protein-protein interactions (PPI) surveys [7], [8] and genome-wide interactome studies [9], but they need to accommodate sensitivity to local environment pH for improved reliability. Although pH effects on protein-small molecule complex calculations are well studied (e.g., refs. [10]–[15]), efforts to incorporate pH effects in computational protein-protein complex calculations have just begun. For example, Spassov *et al*. [16] recently demonstrated a pH-sensitive binding prediction method with an aim to prolong the half-life of therapeutic antibodies. HADDOCK [17] determines the missing protonation state of the histidine residues in the input protein complex using the WHATIF server [18] before the start of the docking simulation. However, in real systems protonation states are affected not only by the solution pH but also the change in the local environment of the ionizable surface residues due to the receptor-ligand interactions during binding. p*K*_a_ calculation studies (e.g. [19]) stress the importance of simultaneously evaluating both favorable residue side-chain conformations and their preferred ionization states. Similarly, in docking algorithms, residue p*K*_a_ values vary depending on the conformations of the ligand relative to the receptor. Hence dynamic evaluation of the protonation states during docking using p*K*_a_ calculation algorithms on-the-fly is more true to the physical process of binding and may improve prediction accuracy.

Current computational p*K*_a_ calculation algorithms have been collectively assessed by the scientific community recently to improve their accuracy [20]. One of the primary aims of the p*K*_a_ calculation methods is to identify and improve the deficiencies of the energy function, particularly the electrostatics [21]. Despite the deficiencies, p*K*_a_ calculations by many algorithms are within a root-mean-square deviation (RMSD) of 1 pH unit from the experimental p*K*_a_ values (except in extreme cases with very large p*K*_a_ shifts [22]–[24]). Hence unless the solution pH is very close to the shifted p*K*_a_ values of the ionizable residues, current algorithms can in principle reasonably estimate the relevant pH-sensitive protonation state during docking. Since computational protein-protein docking algorithms typically generate hundreds to several thousand target conformations, effective use of the protonation state data requires p*K*_a_ calculations to be fast, accurate and compatible with the docking methodology. Unfortunately, the most rigorous physics-based p*K*_a_ calculation methods prohibitively require several minutes to hours to calculate a single p*K*_a_ value, and the faster empirical methods are not currently compatible with the docking frameworks.

We previously created Rosetta-pH [25], a fast and efficient p*K*_a_ calculation algorithm with a focus on the use of the protonation state data in protein structure prediction and design. After we added a pH-sensitive score term to the standard (pH-independent) Rosetta score function and calibrated the electrostatic and solvation score terms, Rosetta-pH achieved a RMSD of 0.83 pH units from the experimental p*K*_a_ values. Since we built Rosetta-pH using the object-oriented Rosetta biomolecular modeling suite [26] which forms the basis for the protein-protein docking algorithm RosettaDock [27], [28], we were able to fuse the methods to create, to our knowledge, the first pH-sensitive protein-protein docking algorithm.

In the remainder of this article, we describe our fast pH-sensitive docking algorithm (pHDock) that can sample side-chain protonation states of five ionizable residue types (Asp, Glu, His, Tyr, Lys) on-the-fly during the docking simulation. After combining the Rosetta-pH and RosettaDock frameworks, we recalibrate the pHDock score function to accommodate the new pH-sensitive score term. We use local docking studies to test pHDock's performance on a dataset of protein-protein complexes [29] and compare it to RosettaDock. We also study the effects of incorporating backbone flexibility in pHDock using a backbone conformational ensemble for docking a subset of the complexes. Finally, we explore a case study to investigate the efficacy of pHDock in the prediction of large pH-dependent binding affinity change in a protein complex [30].

## Results

### pHDock algorithm

We developed pHDock, a multi-scale Monte Carlo (MC) algorithm based on the RosettaDock framework [27], [28] with modifications to allow dynamic sampling of the residue protonation states during simulation. Residue protonation states at the environment pH are constantly updated during multiple side-chain packing steps throughout the protocol by explicitly sampling both protonated and deprotonated versions of the side chains from a discrete rotamer library [31].

**Figure 1:**
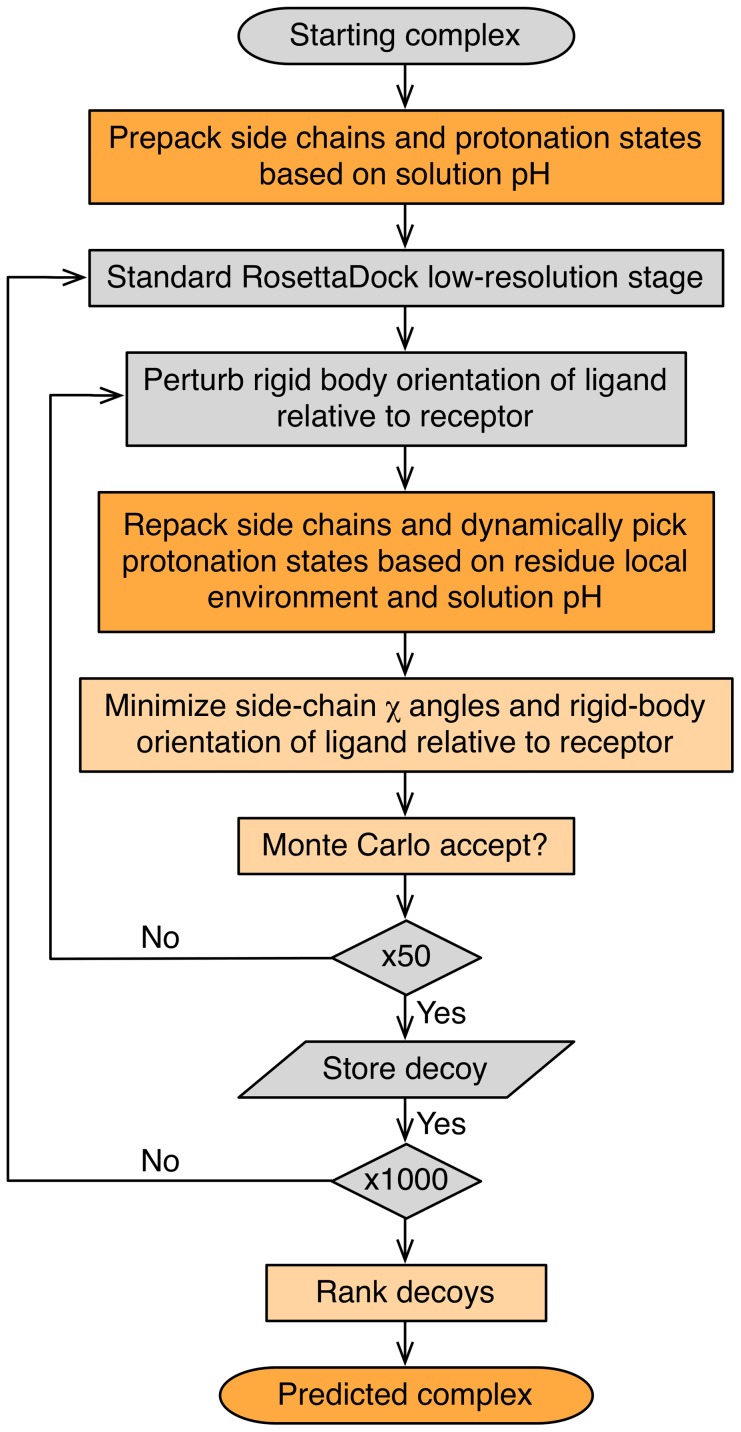
pHDock flowchart. Each step in the pHDock workflow is colored based on the differences compared to RosettaDock: unmodified steps are colored in grey, and steps with minor (light orange) and major (dark orange) modifications are colored in shades of orange.

The pHDock algorithm is illustrated in Fig. 1. In the first pre-packing step, the protein complex side chains are idealized, and the residue ionization states are equilibrated with the solution pH using Rosetta-pH [25]. Then, following the standard RosettaDock low-resolution stage, the residue side chains are represented by coarse-grained centroid atoms. This stage comprises i) a random initial perturbation of the partners, and ii) rigid-body ligand moves relative to the receptor which are accepted/rejected based on the Metropolis criteria. In the high-resolution stage, the side-chain centroid pseudo-atoms are replaced by the side-chain atoms from the initial unbound conformation. The high-resolution stage involves i) repacking the residue side chains with simultaneous evaluation of the most favorable residue protonation states at the environmental pH, and ii) minimization of the side-chain torsion angles and rigid-body orientation of the ligand relative to the receptor with an accompanying Metropolis criteria check. One thousand candidate structures, or models, are generated for each target and then ranked according to their interface scores, and the top-ranked model is picked as the final prediction.

To test the performance of the algorithm, we use both standard RosettaDock (henceforth referred to as simply ‘RosettaDock’) and pHDock to generate local docked models starting from a dataset of unbound structures from the curated Docking Benchmark 4.0 [29]. For pHDock, we assume the crystallization pH of the corresponding bound complex as the solution pH. In the following sections, we first illustrate the docking performance analysis of the new algorithm using a sample protein complex. Next, we compare the performance of pHDock to RosettaDock over the complete benchmark dataset using several metrics and inspect a few predictions in greater detail. We later focus on the effects of backbone flexibility on the docking accuracy. Finally we use a case study to demonstrate pHDock's performance in the prediction of pH effects on binding affinities.

### Sample docking analysis: Xylanase–TAXI-IA binding at non-standard pH

Performance of structural docking algorithms can be analyzed by studying the distribution plots of the free energies or score function vs. the deviation from the starting native bound complex. The native complex is assumed to be at the free energy minimum, hence structural models generated using the docking algorithm with receptor-ligand orientation close to the native structure are expected to have lower energies compared to the structures farther away. To create a set of models sampling both near-native and non-native conformations, starting positions of the ligand relative to the receptor are perturbed by up to 3 Å translation and 8° rotation around the axis joining the centers of the two partners.

**Figure 2:**
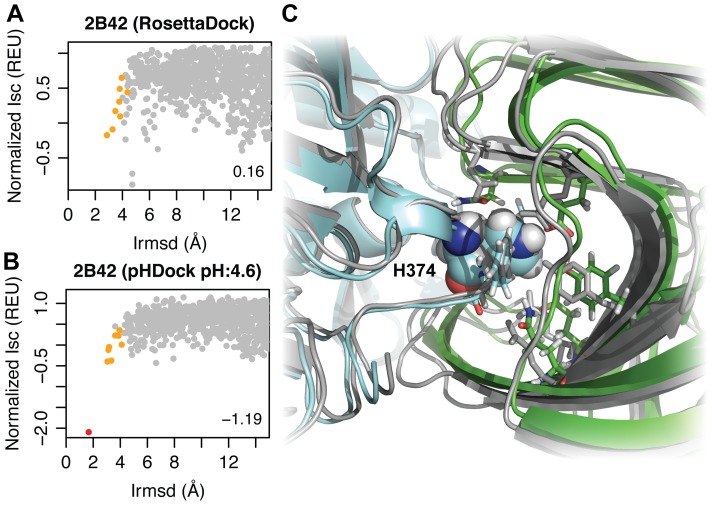
Docking predictions for xylanase – TAXI-IA complex. Docking plots generated by (A) RosettaDock, and (B) pHDock at pH 4.6. Grey, orange, and red points represent incorrect, acceptable-, and medium- quality predictions, respectively. Discrimination scores are shown in the bottom right corner of the plots. (C) Interface of the top-scoring pHDock prediction (medium accuracy) superimposed on the crystal complex (grey) (2B42 [32]). Predicted orientation of the TAXI-IA inhibitor and xylanase, cyan and green, respectively; critical His-374 residue from TAXI-IA, spheres; xylanase active site and other critical binding site residues, sticks.

Fig. 2 shows sample plots for the *Triticum aestivum* xylanase inhibitor-I (TAXI-I) in complex with *Bacillus subtilis* xylanase crystallized at a pH of 4.6 (PDB: 2B42 [32]). The *y*-axis represents the interface score (Isc), an approximation of the binding free energy, normalized by the difference between the 5^th^ and 95^th^ percentile scores. The *x*-axis quantifies deviation from the native complex using interface RMSD (Irmsd). Each point on the plot represents a single docking model and is colored based on the CAPRI structural quality rating [33] (see Methods). The interface of the top-scoring pHDock-generated structure (Fig. 2B) is just 1.7 Å from the native interface, compared to 4.7 Å for the RosettaDock-generated structure (Fig. 2A). While RosettaDock does not generate any structures better than acceptable quality, pHDock produces a structure with higher native residue-residue contact recovery qualifying as medium quality.

We quantified the docking performance using a discrimination score [34] (shown in bottom right in the docking score plot), which captures the extent to which the low-rmsd models have lower energies compared to the high-rmsd (incorrect) models. The discrimination score is calculated by dividing the *x*-axis using multiple Irmsd cut-offs and averaging the energy gaps between the lowest scoring structure on the left and right of each cut-off (see Methods). A lower discrimination score is an indicator of better docking performance, with a negative score indicating a successful docking prediction. The additional side-chain protonation state sampling helps pHDock produce a successful and more pronounced docking funnel (discrimination score: −1.19) compared to RosettaDock (discrimination score: 0.16).

Fig. 2C compares the interfaces of the crystal structure and the top-ranked pHDock model for the xylanase–TAXI-IA complex. Experimental studies [32], [35] discussed the importance of the strong salt bridge between the positively charged imidazole side chain of TAXI-IA His-374 (spheres) with the negatively charged Asp-37. This ionic interaction is critical for binding, and the pH optimum of the xylanase (determined by the p*K*_a_ value of Asp-37) is reported to directly influence the affinity of the enzyme–inhibitor complex, with a lower Asp p*K*_a_ value leading to stronger binding. The top-scoring pHDock model not only captures this interaction through precise prediction of the positively charged His-374 side-chain rotamer but also recovers all the xylanase active-site-residue side-chain rotamers. RosettaDock, which assumes a neutral His side chain, fails to capture the interaction. Overall, while the top-scoring RosettaDock model recovers just 13% of the native interface contacts, the pHDock model recovers 49% of all the interface residue-residue contacts.

### pHDock improves docking accuracy in a majority of docking targets

**Figure 3:**
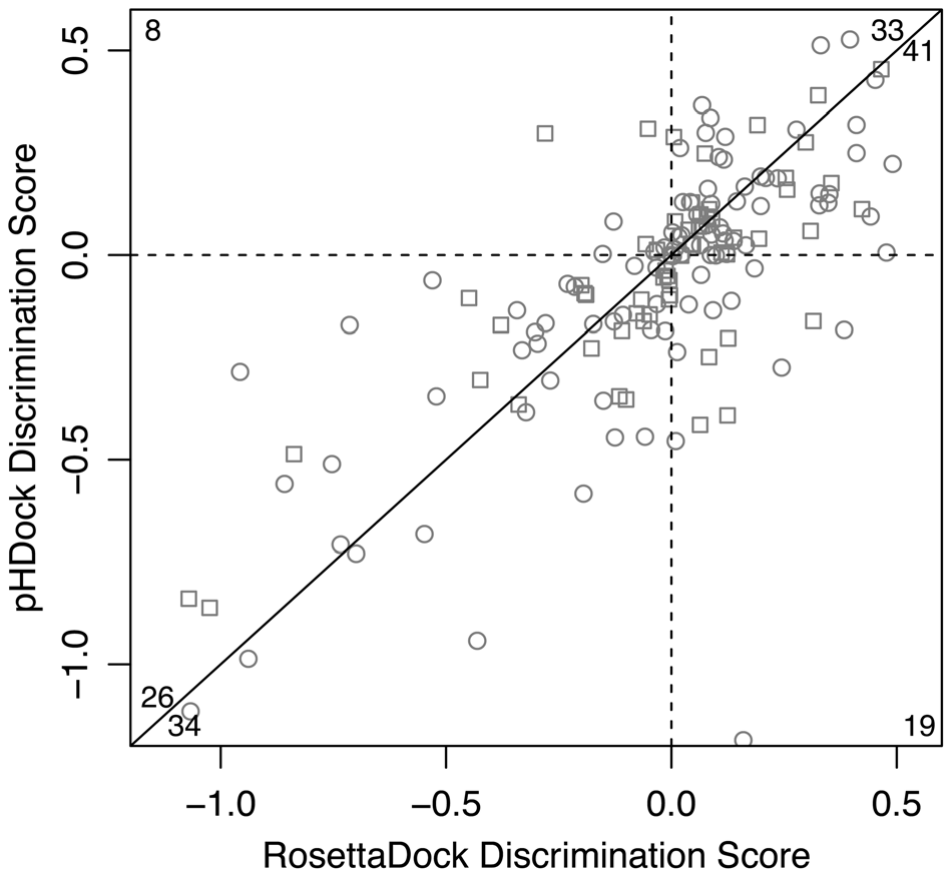
Summary of pHDock performance. Correlation plot comparing discrimination scores of pHDock and RosettaDock docking predictions for each target in the complete benchmark dataset. Complexes docked at acidic pH (pH≤7.0) and basic pH (pH>7.0) are represented as circles and squares, respectively. The discrimination score cutoffs for a successful prediction (*D*<0) are marked using broken lines. Corner numbers indicate the total predictions in each plot section (edges defined by the broken lines and the solid line at 45°).

For a large-scale docking performance analysis, we tested pHDock over a dataset of diverse protein–protein complexes from the curated Docking Benchmark 4.0 [29]. On average, 25% of the interface residues in the dataset complexes are ionizable (Asp, Glu, His, Tyr, Lys) (S1 Figure). Fig. 3 compares the discrimination scores of the docking funnels generated using pHDock and RosettaDock. pHDock produces successful docking funnels (discrimination score ≤0) in approximately half (79/161) the structures from the dataset, including 19 cases where RosettaDock fails to produce a successful prediction. Based on the discrimination score, pHDock outperforms RosettaDock in approximately 60% of the targets (94/161) (Table S2), and the improvements are statistically significant (paired *t*-test, *p* = 0.039). Additionally, since models are generated stochastically, we performed bootstrap case resampling [36] to quantify the variation of the discrimination scores. The bootstrap mean discrimination scores µ(*D*) (S2 Figure) again show that pHDock produces successful funnels [µ(*D*) ≤0] in half the targets (79/161) including 17 cases where RosettaDock fails. Hence the results are robust to the stochastic sampling noise. The average standard deviation of the discrimination scores [σ(*D*): 0.07] is approximately 4% of the total observed µ (*D*) range.

As pHDock has access to nonstandard residue protonation states unlike RosettaDock, we examined the prevalence of such protonation states and their effect on docking accuracy. In docking funnel plots in S9 Figure, structures with nonstandard residue protonation states are distinguished. pHDock produces models with nonstandard protonation states for all the target complexes (S3 Figure), with a majority of the nonstandard protonation states observed in complexes with docking pH within one pH unit of the residue intrinsic p*K*_a_ values (S4 Figure). Overall, pHDock outperforms RosettaDock in 67% (20/30) of the cases where the top-ranked pHDock model recovers a nonstandard protonation state observed in the native bound complex (S5 Figure). pHDock also performs better than RosettaDock in 64% (7/11) of the cases where the top-ranked pHDock produces a nonstandard protonation state different from the one observed in the native bound complex illustrating the importance of dynamic protonation states.

**Figure 4:**
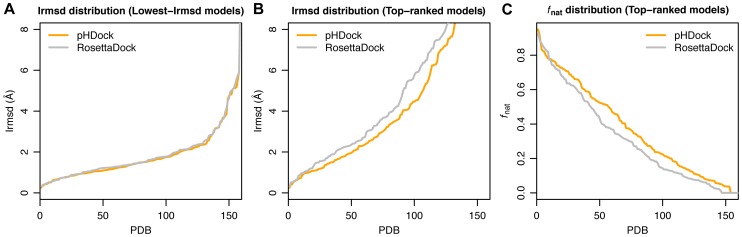
Distribution curves of interface RMSDs (Irmsd) and fraction of recovered native contacts (*f*_nat_) for the docking models. (A) Irmsd distribution curve of the lowest-Irmsd models generated using pHDock (orange) and RosettaDock (grey). (B, C) Irmsd and *f*_nat_ distribution curve for the top-ranked models according to interface scores (Isc) for each protein complex. The distribution curves are generated after independent sorting of the pHDock and RosettaDock models based on (A, B) increasing Irmsd values and (C) decreasing *f*_nat_.

Since pHDock is a stochastic docking algorithm that generates several candidate models, the performance of the algorithm broadly depends on (i) the quality and diversity of the generated ensemble of models, or ‘sampling’, and (ii) the ability of the final score function to discriminate native-like models from non-native-like models, or ‘scoring’. To test the sampling performance of pHDock, we examined the lowest-Irmsd models for all the complexes in the dataset. The Irmsd distribution for pHDock is similar to RosettaDock (Fig. 4A), and in 92% of the docking targets, it generates at least one model within 4 Å from the native interface. Out of 1000 models generated for each target, pHDock creates on average 1.9, 18.5, and 90.8 high-, medium-, and acceptable-quality models, respectively. In comparison, RosettaDock samples 7–12% fewer medium- and high-quality models (S6 Figure). To test the scoring performance of pHDock, we calculated the Irmsd and *f*_nat_ distributions of the top-scoring models for each target (Figs. 4B–C). pHDock generates top-ranked models within 4 Å in 57% of the targets (RosettaDock 51%), and 52% of the time these models recover more than 30% of the native residue-residue contacts (RosettaDock 46%).

**Figure 5:**
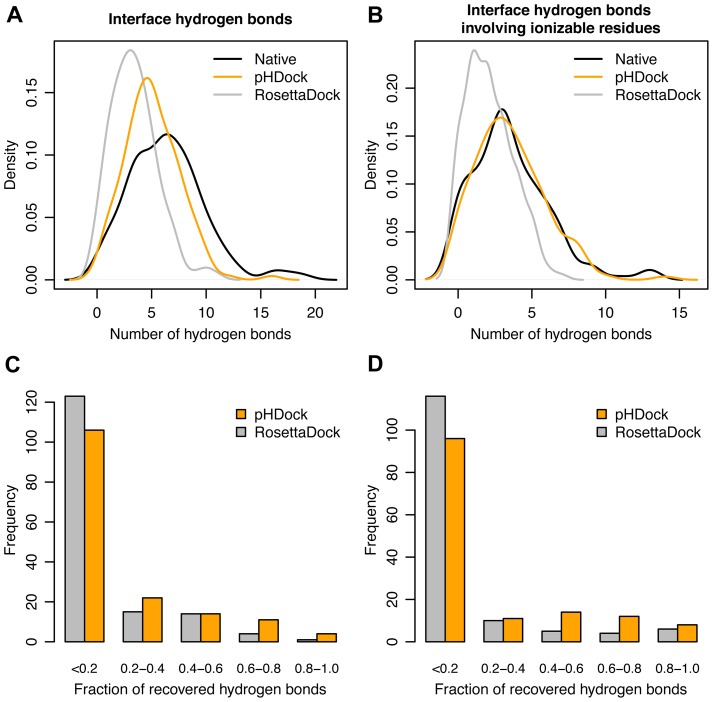
Distributions of native and model interface hydrogen bonds. Kernel density estimate curves for the number of (A) interface hydrogen bonds and (B) interface hydrogen bonds involving ionizable residues in the top-scoring models generated using pHDock (orange) and RosettaDock (grey), and the native crystal complexes (black) across the complete Docking Benchmark dataset. Frequency histograms of the fraction of (C) recovered interface hydrogen bonds and (D) recovered interface hydrogen bonds involving ionizable residues in the top-scoring models.

To further assess the quality of the predicted top-ranked structures, we examined the receptor-ligand interface hydrogen bonds (henceforth referred to as simply ‘interface hydrogen bonds’). Previous surveys found 8–13 interface hydrogen bonds in each protein–protein complex [37], [38]. Using Rosetta's hydrogen bonding definition, the native crystal complexes in our dataset contain 6.4±3.5 interface hydrogen bonds on average (Fig. 5A). In comparison, the top pHDock models are involved in 5.1±2.5 interface hydrogen bonds, while the top RosettaDock models form only 3.4±2.1 interface hydrogen bonds. As pHDock primarily focuses on ionizable residues, we also calculated the number of interface hydrogen bonds containing such residues as donors or acceptors. The native complexes contain 3.5±2.6 ionizable interface hydrogen bonds (Fig. 5B). Encouragingly, the top pHDock models are found to form an identical 3.5±2.4 ionizable interface hydrogen bonds, while the top RosettaDock models form only 2.1±1.6 hydrogen bonds.

The analysis of the total number of interface hydrogen bonds shows significant pHDock improvements in generating models with a larger receptor-ligand hydrogen bond network. However, such an analysis does not reveal the accuracy of the generated interface hydrogen bonds. So we also examined the fraction of the native interface hydrogen bonds recovered in the top-ranked models. pHDock recovers more than one-fifth of the native interface hydrogen bonds in only 33% of the targets from the dataset, while RosettaDock performs worse, recovering the same fraction in just 22% of the targets (Fig. 5C). The results are similar for the fraction of recovered ionizable interface hydrogen bonds. pHDock recovers more than one-fifth of the ionizable interface hydrogen bonds in 32% of the targets, while the performance of RosettaDock drops further to just 19% of the total targets in the dataset (Fig. 5D). In summary, while pHDock generates more interface hydrogen bonds, only a minor faction of these hydrogen bonds match those seen in the native complex.

**Figure 6:**
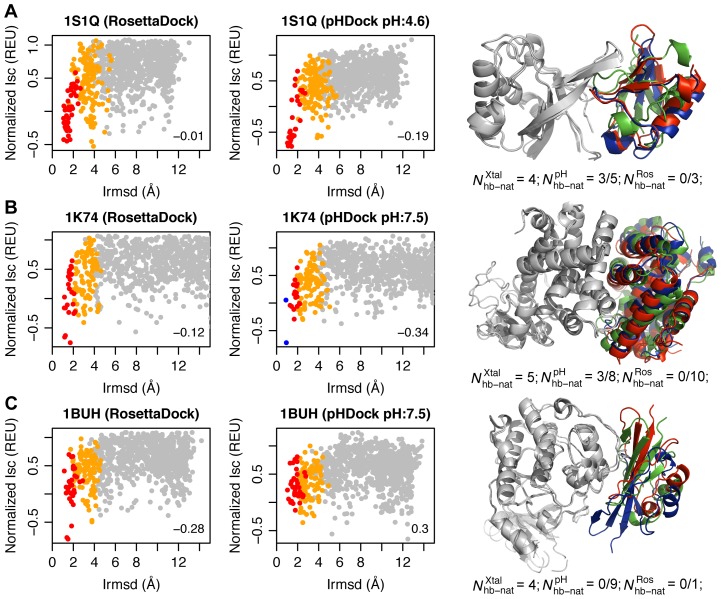
Hydrogen bonding recovery correlates with docking performance. Docking plots generated using RosettaDock and pHDock for (A) tumor susceptibility gene 101 protein–Ubiquitin complex (1S1Q; pH 4.6), (B) PPARgamma+RXRalpha–GW409544+co-activator peptide complex (1K74; pH 7.5), and (C) CDK2 kinase–cell cycle-regulatory protein CksHs1 complex (1BUH; pH 7.5). Grey, orange, red, and blue points represent incorrect, acceptable-, medium-, and high-quality models, respectively. Discrimination scores are shown in the bottom right corner of the plots. The right panel shows structures of the top pHDock (blue) and RosettaDock (green) models superimposed on the native complex (red). The number of native hydrogen bonds among the total interface hydrogen bonds observed in the bound crystal complex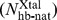, and the top-scoring pHDock
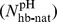
and RosettaDock
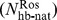
models are also listed.

Finally, to test the effects of hydrogen bonding accuracy on docking results, we examined a few sample cases in greater detail. The tumor susceptibility gene 101 protein–ubiquitin complex (1S1Q; pH 4.6 [39]) has four native interface hydrogen bonds. The top pHDock model recovers three of them and forms a total five interface hydrogen bonds, while the top RosettaDock model exhibits three interface hydrogen bonds but none of them are native. The docking plots for both pHDock and RosettaDock (discrimination score −0.19 vs. −0.01) show success based on discrimination scores, but the docking funnel is clearly more pronounced in pHDock (Fig. 6A). Although the near-native sampling in both pHDock and RosettaDock is comparable, the additional recovered native hydrogen bonds help pHDock in the final scoring, and the top model interface is only 1.4 Å away from the native interface. The improved performance is likely due to a protonated interface histidine (His-66) in ubiquitin. In a second case, the PPARgamma+RXRalpha–GW409544+co-activator peptide complex (1K74; pH 7.5 [40]) has five interface hydrogen bonds. The top pHDock model exhibits eight interface hydrogen bonds, three of them being native, while none of the ten hydrogen bonds found in the top RosettaDock model are native (Fig. 6B). In this case, pHDock (discrimination score −0.35) outperforms RosettaDock (discrimination score −0.12) in both sampling and scoring (Fig. 6B). The top-scoring pHDock model is a high-quality prediction just 0.93 Å from the native interface. In this case, the interface residues are all in their standard protonation states; we infer that the improvement must be due to kinetic effects during the Monte Carlo docking search. The larger number of interface hydrogen bonds in pHDock models do not always translate to improvements in docking predictions. For example, the CDK2 kinase–cell cycle-regulatory protein CksHs1 complex (1BUH; pH 7.5 [41]) has four native hydrogen bonds. Again, the interface residues in the top-ranked pHDock model are predicted to be in their standard protonation states. Neither top pHDock nor RosettaDock models recover any of the native interface hydrogen bonds although they form nine and one interface hydrogen bonds, respectively. As shown in the docking plots in Fig. 6C, pHDock scoring favors a false-positive docking prediction with a large number of interface hydrogen bonds more than 12 Å from the native interface.

### Backbone flexibility further improves native contacts and hydrogen bond recovery

Inclusion of backbone flexibility in protein-protein docking is critical to capture the conformational changes during the binding event [42]. Within RosettaDock, backbone flexibility mimicking both conformer selection (CS) and induced fit (IF) binding models increases native contact recovery, although the computational costs are higher and there is a risk of false positive predictions [43]. Thus we tested whether the addition of backbone flexibility further improved native contact recovery in pHDock. We chose a subset of 14 complexes common among the published study and the curated Docking Benchmark 4.0 used for pHDock (Table S3). We then used the RosettaRelax [44], [45] protocol to generate an ensemble of unbound backbones. RosettaRelax, an MC algorithm, employs a cycle of small backbone dihedral (φ, ψ) perturbations, residue side-chain packing and score function minimization along the gradient in the torsion space to generate a backbone ensemble typically within 1 Å C_α_ RMSD of the starting structure. We generated 500 models starting from the ligand unbound coordinates for each of the complexes and picked the ten top-scoring models for docking.

S10 Figure compares the docking funnels generated using RosettaDock, pHDock and ensemble pHDock. The ligand backbone flexibility helps ensemble pHDock generate better docking funnels (based on discrimination score) in 11 targets compared to pHDock. The Irmsd values of the lowest-Irmsd models generated using ensemble pHDock are not significantly better compared to pHDock. However, there is a noticeable improvement in the quality of the receptor-ligand interfaces in the top-ranked models. The top-ranked models generated using ensemble pHDock outperform pHDock in native contact recovery with comparable or better *f*_nat_ values in 12 targets. Encouragingly, the top-ranked models also recover comparable or more native interface hydrogen bonds in all the targets compared to pHDock and RosettaDock (Table S3). To summarize, the additional backbone flexibility further improves the docking funnel quality in a majority of the targets and generates top-ranked models that recover more native contacts and hydrogen bonds.

### pHDock is better at solution pH than pH 7 or using fixed, predetermined protonation states

pHDock simulates the complexes at solution pH and relies on dynamic residue protonation state sampling. To assess the individual contribution of these two components, we performed control docking experiments using a subset of complexes (same 14 complexes used for ensemble pHDock). First, to test the robustness of the docking predictions to changes in the solution pH, we used pHDock at physiological pH (pH 7.0). Second, to test the benefits of employing dynamic residue protonation states, we docked the complexes with fixed residue protonation states obtained from the lowest energy rotamer state of the starting partners at the solution pH (fix-pHDock).

Of the cases where both RosettaDock and pHDock either fail (four targets) or succeed (eight targets), the fix-pHDock and pHDock at pH 7.0 runs perform similarly (see docking funnel plots, S11 Figure), showing, as might be expected, an insensitivity to pH effects. There are two cases in this test set where RosettaDock fails and pHDock produces a successful docking funnel. In the α-chymotrypsin–eglin C complex (1ACB; pH 6.5 [46]), pHDock produces a discrimination score of −0.24 at pH 6.5, and RosettaDock a discrimination score of 0.01. pHDock at pH 7.0 produces a weaker funnel (discrimination score: −0.1) while fix-pHDock fails (discrimination score: 0.09) due to a false positive model 7 Å Irmsd away from the native complex. Similarly, in the Fab D44.1–lysozyme complex (1MLC; pH 6.0 [47]), pHDock generates a discrimination score of −0.11 while RosettaDock, pHDock at pH 7.0, and fix-pHDock all fail (discrimination scores 0.13, 0.07, 0.33, respectively). Thus, in these two cases where RosettaDock fails, both pHDock at pH 7.0 and fix-pHDock fail to completely capture pHDock's success. These cases suggest that accurate knowledge of the solution pH and the dynamic protonation states are vital for maximum pHDock accuracy.

### pHDock captures the large pH-dependent binding affinity change in the Fc–FcRn complex

In the discussion so far, we analyzed pHDock's performance at the solution pH and compared it to RosettaDock (no pH dependence) over a large dataset of protein complexes. However, such an analysis does not test pHDock's performance in predicting effects of subtle environmental pH changes on a single protein-protein complex. In previous work, we and other groups have used RosettaDock interface scores in correlating binding affinities [48], [49] and in predicting relative affinities [50]. The neonatal Fc receptor (FcRn) binds maternal immunoglobulin G (IgG) from ingested milk in the gut at acidic pH (pH≤6.5) and releases it in the bloodstream of the newborn at basic pH (pH 7.4) [51]. This process is facilitated through a drastic drop in the binding affinity by more than two orders of magnitude as the pH changes from 6.0–6.5 to 7.0–7.5 [51], [52]. The Fc–FcRn system has been previously used for a pH-dependent binding calculation [16], but to our knowledge, there are no existing pH-sensitive docking studies.

**Figure 7:**
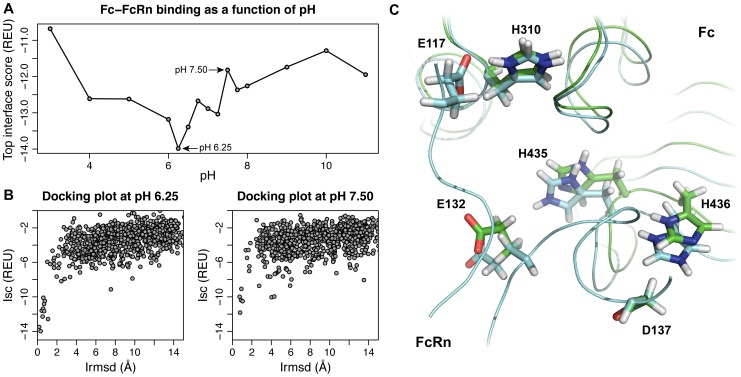
pH-dependent binding effects in Fc–FcRn complex. (A) Interface score of the top pHDock prediction for the Fc–FcRn complex as a function of the docking pH. (B) Interface score vs Irmsd plots generated using pHDock at pH 6.25 and pH 7.50. (C) Top pHDock models at pH 6.25 (cyan) and pH 7.50 (green) showing the three critical ionic interactions responsible for the large pH-dependent binding affinity change. Note the change in the protonation states of His-435 and His-436.

To test the efficacy of pHDock in predicting pH effects on binding affinities, we used the pHDock algorithm to dock the murine Fc–FcRn complex (1I1A [30]) at various environmental pH values. We tested all integral pH values between 3.0 and 11.0, and used a finer interval of 0.25 pH units for the relevant pH range of 6.0–8.0 where the striking binding affinity change is observed. We used the interface scores (*I*) of the top-scoring pHDock models to approximate the binding affinity at different pH values. Fc–FcRn complex shows a binding minimum at pH 6.25 (*I*_pH6.25_: −13.99 Rosetta Energy Units (REU)), and thereafter the affinity rapidly weakens as the environment pH increases to 7.50 (*I*_pH7.50_: −11.82 REU) (Fig. 7A). Converting the binding energies to equilibrium constants using the relation 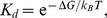
we estimated the ratio of equilibrium constants at pH values 6.25 and 7.50 as 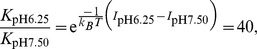
where *K*_pH6.25_ and *K*_pH7.50_ are the equilibrium binding constants at pH 6.25 and 7.50, respectively, and *k*_B_T is 0.59 kcal/mol at 298K. The equation yields a 40-fold drop in the binding affinity as the pH increases from 6.25 to 7.50, which is similar to the 50 to 100-fold drop from experiments [52]. Interestingly, the docking plots show successful energy funnels for both pH values (Fig. 7B). However, the energy funnel is more pronounced at pH 6.25 (discrimination score −0.96) than pH 7.50 (discrimination score −0.47), indicating a site-specific binding event at both pH values, but with markedly different affinities.

Previous studies [30], [51] attribute the pH-dependence of Fc–FcRn binding to the titration of interface histidine residues with p*K*_a_ values in the range of binding affinity transition (6.5≤pH≤7.0). The Fc–FcRn interface has three salt bridges with the residues His-310, His-435, and His-436 from Fc interacting with Glu-117, Glu-132, and Asp-137 from FcRn. The proposed mechanism involves titration of all the three histidine residues disrupting the binding as the environment pH increases, but studies have shown two buried titratable salt bridges are sufficient to confer pH dependence. Encouragingly, the top-scoring pHDock-generated models at different pH values successfully capture the titration event. While His-310 remains protonated in the models at both pH values, His-435 and His-436 are protonated at pH 6.25 and deprotonated at pH 7.50 and are involved in salt bridges with Glu-132 and Asp-137, respectively (Fig. 7C). Thus, pHDock not only predicts the relative Fc–FcRn binding affinities at different pH values, but also captures the expected physical mechanisms responsible for the different affinities.

## Discussion

We have created pHDock, the first pH-sensitive protein-protein docking algorithm that samples residue protonation states dynamically during the search. The algorithm integrates the Rosetta-pH p*K*_a_ calculation method [25] with the RosettaDock framework using the object-oriented design of the Rosetta modeling suite [26]. Local docking studies show that pHDock outperforms RosettaDock in 60% of the docking targets and also performs better than control cases involving docking at pH 7.0 or using fixed, predetermined protonation states. pHDock also shows encouraging improvements in the quality of the generated candidate predictions. On average, the top-ranked pHDock structures have lower interface RMSDs and recover more native residue-residue contacts and hydrogen bonds. While pHDock is designed to improve docking predictions by accounting for environmental pH effects, the successful prediction of a large pH-dependent binding affinity change in the Fc–FcRn complex suggests that it can be further exploited to improve affinity predictions.

pHDock improves docking primarily by enhancing the scoring in the docking high-resolution stage, as the improved score function finely tuned for p*K*_a_ predictions is active only during the high-resolution steps involving dynamic protonation states. Although there are few cases where pHDock samples conformations closer to the native compared to RosettaDock, the similarity of the interface RMSD distributions of the closest-sampled models (to the native complex) shows that its sampling quality is largely unchanged, likely because it retains the RosettaDock low-resolution stage which is largely responsible for model diversity. Over the complete dataset, pHDock generates at least one high-quality model in 25% of the complexes (41 targets), slightly higher than RosettaDock (34 targets). ReplicaDock [53], which uses a set of temperature replicas, overcomes the kinetic barriers and improves sampling in the low-resolution docking stage. Further work can thus focus on combining the principles of ReplicaDock with pHDock to improve the model diversity in the low-resolution centroid phase. Also, availability of even sparse biochemical information [54] can be used as an alternative to constrain the conformational search space and circumvent the sampling concerns in the centroid phase to improve docking accuracy.

Although the top-ranked pHDock models show significant advancements in recovering native contacts, the hydrogen bonding performance is mixed. The geometry of interface hydrogen bonds is less optimal than intra-chain hydrogen bonds, but they are nevertheless critical for protein-protein binding [37]. The top pHDock models exhibit more hydrogen bonds than RosettaDock on average. The increase is especially evident in the case of ionizable residues where the pHDock hydrogen bond distribution matches the native distribution. However, many of the pHDock interface hydrogen bonds are non-native, *i.e*., they are not observed in the bound crystal complexes. In fact, in two-thirds of the targets, pHDock fails to recover more than one-fifth of the native interface hydrogen bonds, a shocking number revealing the limitations still present in the hydrogen bonding model.

There are a few possible explanations for the poor hydrogen bond performance. First, pHDock uses an implicit solvation model and thus fails to capture the water-mediated interface hydrogen bonds. Although the water-mediated hydrogen bonds are excluded from native hydrogen bond calculations, ignoring the water molecules during docking can result in the compensation of unsatisfied hydrogen bond donors/acceptors through formation of non-native hydrogen bonds. Second, pHDock ignores protein backbone flexibility and uses the unbound coordinates of the protein partners for docking, hence any resulting backbone inaccuracies can shift the hydrogen bond network. Accounting for backbone flexibility using a conformational ensemble for a small subset of complexes improves hydrogen bond recovery compared to pHDock, but the top-ranked models still recover just a quarter of the native interface hydrogen bonds. Further studies to improve hydrogen bond recovery can focus on calibrating the score function using the bound coordinates of the complex to minimize the errors introduced due to the rigid backbone assumption and the inaccuracies in the receptor-ligand orientation in the docking models. However, work will be needed to reconcile the changes with the docking score function that is tuned for recovering native-like structures.

We tested pHDock's ability to capture the large pH-dependent binding affinity change in the Fc–FcRn complex. Since the binding changes are a result of protonation state shifts in the interface histidine residues, any docking algorithm ignoring environment pH will fail to capture the effect. pHDock predicts a 40-fold drop in the binding affinity due to the increase in the environment pH, and the top-scoring model captures the resulting disrupted salt bridges at the Fc–FcRn complex interface. The accuracy of the affinity prediction suggests that pHDock can be expanded to power computational protein design studies such as those that recently began to exploit the pH-dependence for regulating protein binding activity [55]. Previously during the CAPRI rounds 20–27 [56], we used pHDock for the blind prediction of the g-type lysozyme–PliG inhibitor complex [50]. Lysozyme operates in a low pH environment [57] and hence provided an opportunity to test pHDock's performance. Docking the complex at pH 6.2 (crystallization pH of the unbound lysozyme) generated a medium-quality prediction just 2.0 Å from the interface of the native complex. The encouraging performance of pHDock proves that it can be effective in capturing environment-pH effects on both docking and binding.

Recent efforts have begun to capture structural details of protein interactions in complete cellular environments [58]–[60]. There is tremendous scope for computational docking algorithms to power such studies, but the methods must be versatile and include the effects of environmental conditions. Since intracellular pH is strictly regulated across multiple eukaryotic cellular compartments and is critical for protein interactions [61], accounting for pH effects can boost prediction accuracy. The results in this paper contribute to the community effort to simulate protein-protein interactions in the complete cell with all environmental factors.

## Methods

### Benchmark dataset

The Protein-Protein Docking Benchmark 4.0 by Hwang *et al.* [29] is a set of 176 non-redundant protein-protein complexes with both bound and corresponding unbound crystal coordinates from the Protein Data Bank [62]. The dataset comprises 121 ‘rigid-body’, 30 ‘medium’, and 25 ‘difficult’ targets based on the interface backbone conformation variation between bound and unbound coordinates [63].

We curated the benchmark dataset in multiple stages. First, we removed water and all non-peptide molecules containing heteroatoms from the complex structures. Since Rosetta pH does not currently predict protonation states of non-peptide molecules, we excluded complexes with such molecules at the interface. We also eliminated structures in which Rosetta was unable to resolve the steric clashes in the starting atomic coordinates due to the conformational changes between bound and unbound complexes, leaving 161 test complexes for the study. Second, we truncated both the unbound and bound structures to the same amino-acid sequences for Rosetta scoring consistency. Third, we collected the crystallization pH values in the PDB coordinate file for each bound complex to determine the docking environment pH. For structures missing pH information in the PDB files, we used the pH value from the corresponding original research article if available. For the remaining structures, we assumed a physiological pH of 7.0 (Table S2).

### Rosetta-pH

Rosetta-pH [25] is a Metropolis Monte Carlo algorithm in which the protonation state of the lowest energy conformation is evaluated using the Rosetta-pH score function at intervals of pH to estimate p*K*_a_ values. The Rosetta-pH score function is based on the standard Rosetta score function with additional terms including:

i) Protonation potential based on the probability of protonation of individual amino acid residues at a given pH. The probability of protonation 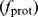 of an amino acid is 
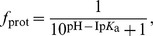
and the protonation potential (*E*_pH_) is



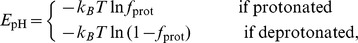
where pH is defined by the environment, and Ip*K*_a_ is the unperturbed intrinsic p*K*_a_ value of the model compound in solution (4.0 for Asp, 4.4 for Glu, 6.3 for His, 10.0 for Tyr and 10.4 for Lys). *k*_B_T is assigned a value of 0.59 kcal/mol, corresponding to *T* = 298K. Cys protonation state changes (intrinsic p*K*_a_ 8.5) are ignored due to the complications of coupling between p*K*_a_ and redox equilibrium [64].

ii) Coulomb electrostatic potential with a distance-dependent dielectric (*ε* = 10*r*) for gradual shielding at increasing interatomic distances [65], and

iii) Recalibrated solvation reference energies 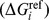 for the non-standard protonation variants in the Lazaridis–Karplus implicit model for solvation [66] (See [25] for details).

### pHDock development

Rosetta pHDock uses the object-oriented design of the Rosetta biomolecular modeling suite [26] to implement the environment pH effects in the RosettaDock protocol. The pHDock development workflow can be broadly classified into three stages:

i) In the first stage, we incorporated explicit protonation state sampling from Rosetta-pH [25] into the RosettaDock algorithm. RosettaDock accounts for residue side chain flexibility in the prepacking step and the later high-resolution stage with full-atom side chains. The sampling of the side-chain *χ*-angles is discrete based on a backbone-dependent rotamer library [31]. Rosetta pHDock augments the sampling by allowing variable residue ionization states to be simultaneously sampled during every side-chain packing step and picking the most favorable residue protonation state based on the residue's local interactions and the solution pH. For neutral His, both possible tautomers (with proton on either N*_δ_*_1_ or N*_ε_*_2_ atoms) are sampled. The conformational degeneracy in the protonated variants of Asp and Glu (with H atoms on either of the terminal O*_δ_* and O*_ε_* atoms, respectively) is also explicitly incorporated by accommodating both possible protonated versions for the residues during sampling.

ii) In the second stage, we generated a dataset of structures and evaluated the contributions of the individual score terms (including e_pH) to the total interface score. We first generated 1000 models (for each complex) using the standard RosettaDock local docking routine [28] on a subset of 60 randomly-selected bound complexes (∼1/3 of the total docking benchmark set). We then repacked each model (sampling both side chains and protonation states) at the crystal pH of the bound complex and calculated the interface contribution of each score term 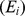 as
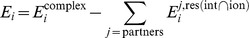
where 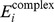 is the contribution of the score term *i* in the repacked complex, and 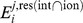
is the score term contribution in each separate binding partner *j* after repacking the ionizable interface residues at the crystal pH of the bound complex. Repacking the ionizable residues is required for accurate score term estimation, as separation of the binding partners exposes the previously-buried interface residues to the solvent affecting their preferential protonation state.

iii) In the third stage, we parameterized the pHDock score function. Reweighting is mandatory since the original RosettaDock score function had a minimal weight on electrostatics, and the new electrostatic weight and pH reference term must be rebalanced against the hydrogen bonding and solvation contributions. Similar to prior parameterization of the RosettaDock score function [27], we sought to maximize the free energy gap between ‘near-native’ and ‘non-native’ models. Models in the top 5% based on CAPRI rating [33] (high, medium and acceptable-quality in that order) with repulsive van der Waals scores lower than the 80^th^ percentile are classified as near-native models. Models with the same CAPRI rating are ordered based on the *f*_nat_ values (higher *f*_nat_ is better). We classified the remaining models as non-native models. We then derived the score term weights using a generalized linear regression to maximize the free energy gap between the near-native and non-native model clusters. The free energy gap (Δ*E*) is
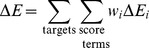
where *w_i_* is the weight for score term *E_i_* and 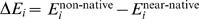. The score terms include an attractive van der Waals score (*E*_atr_), a repulsive van der Waals score (*E*_rep_), an implicit solvation score (*E*_sol_) [66], a hydrogen bonding score (*E*_hb_) [67], rotamer probability term (*E*_dun_) [31], a statistical residue pair term for ion-ion interactions (*E*_pair_) [68], a Coulomb electrostatic term (*E*_elec_), and a term for the pH effects (*E*_pH_) [25].

Table S1 compares the optimized pHDock weights to the RosettaDock weights. The new pHDock weights for the dominant score terms *E*_atr_, *E*_sol_, and *E*_hb_ show small deviations compared to RosettaDock (0.377, 0.225, and 0.249 *versus* 0.338, 0.242, and 0.245). Besides the new addition of pH-sensitive score term *E*_pH_ (weight 0.21), the major changes in the score function are in the score term weights for *E*_pair_, *E*_elec_, *E*_dun_, and *E*_rep_. The *E*_pair_ term is completely absent and is balanced by the increased *E*_elec_ weight (0.319 compared to 0.026 in RosettaDock). While the *E*_dun_ weight also increases (0.036 to 0.080), the *E*_rep_ weight drastically drops from 0.044 to 0.005 demonstrating that the repulsive van der Waals score does not aid in docking model discrimination. The exceptionally small *E*_rep_ weight however creates two issues. First, the algorithm produces structures with steric clashes during the rigid-body minimization step in the docking high-resolution stage (Fig. 1). RosettaDock [27] addresses this issue by increasing the *E*_rep_ weight during minimization using a multiplier. We followed the same strategy and raised the *E*_rep_ weight to match the RosettaDock weight during minimization. Second, some structures with unfavorable sterics are ranked higher during the final model discrimination. To address this, we eliminated the worst 5% percent of the pHDock structures sorted by their *E*_rep_ scores. For a balanced comparison, we also omitted the worst 5% of the RosettaDock structures sorted by their interface scores.

### Docking starting conformation generation

In local docking, the input complex consists of unbound partners (orientation determined by superimposing on the coordinates of the bound complex) and the starting positions are generated by randomly perturbing the ligand relative to the receptor by up to 3 Å translation and 8° rotation around the axis joining the centers of the two partners. Both pHDock and RosettaDock use local docking to generate a diverse set of models sampling both near-native (Irmsd <4 Å) and non-native (Irmsd>4 Å) conformations around the binding site.

### Docking metrics

The CAPRI structural quality rating [33] classifies docking predictions as incorrect, acceptable-, medium-, or high-quality based on a combination of the metrics Lrmsd, Irmsd, and *f*_nat_. L_rmsd is defined as the root-mean-square deviation (RMSD) of the ligand C_α_ atoms after superposition of the receptor chains of the predicted and the native bound complexes. Irmsd is the C_α_-atom RMSD after superposition of the interface residues (residues <4.0 Å from the binding partner) with coordinates from the bound complex. *f*_nat_ is the fraction of the residue-residue contacts (<5.0 Å all-atom distance) in the native bound complex that are recovered in the predicted complex. CAPRI ratings depend on multiple criteria, but models are considered to be at least acceptable quality if they are within 4 Å from the native interface and recover at least 30% of the native contacts (*f*_nat_) [33].

### Docking funnel metrics

A ‘docking funnel’ derives its name from the funnel-like appearance of the target score vs RMSD plots where the near-native models have better scores than non-native models. It is often used as a measure to determine the success of a docking simulation. We used two different metrics to quantify docking funnels.

i) N_5_: As defined by Chaudhury *et al*. [28], *N*_5_ is the number of models with an Irmsd of at most 4.0 Å among the five top-scoring structures based on interface score. A docking result is considered a success if *N*_5_≥3. We performed bootstrap case resampling (1000 models per target with replacement) to compare correlation between the mean µ(*N*_5_) and calculated *N*_5_, and to quantify the inherent noise within set of models using the standard deviation σ(*N*_5_) (S7 Figure).

ii) Discrimination score (D): Applying the formulation by Conway *et al.* [34] to docking, we first normalize the model interface scores (*Î*) using the 5^th^ and 95^th^ percentile scores as the reference by assigning them values of 0 and 1, respectively. The models are then divided into clusters based on Irmsd with cut-offs from 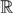 =  {1.0, 1.5, 2.0, 2.5, 3.0, 4.0, 6.0} in Ångstroms. Discrimination score (*D*) is defined as the normalized interface score difference of the lowest-energy model below and above each cut-off *r*∈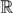, averaged over the number of cut-offs (*N_r_*):
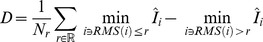



A docking result is considered a success if *D*≤0. We performed bootstrap case resampling (1000 models per target with replacement) to quantify the inherent noise within the set of models using the standard deviation σ(*D*) (S2 Figure).

### Algorithm availability

pHDock is part of the Rosetta biomolecular modeling suite (www.rosettacommons.org) which is freely available for academic and non-profit use. The Supporting Information includes the complete list of structures from the docking benchmark dataset with the corresponding pH values and the command-line syntax for using pHDock method in Rosetta. Component methods and objects are also available in the PyRosetta libraries (www.pyrosetta.org) [69].

## Supporting Information

S1 Figure **Distribution of ionizable residues at docking interfaces.** Frequency histogram of the number of dataset complexes with various fractions of ionizable interface residues (Asp, Glu, His, Tyr, Lys).
(TIF)

S2 Figure **Discrimination score (** ***D*** **) distributions for RosettaDock and pHDock algorithms.** Mean *D* (µ(*D*)) values obtained from bootstrap case resampling of the docking models (1000 models per target with replacement) for pHDock (orange) and RosettaDock (grey). Standard deviations (σ(*D*)) are represented as error margins. The average µ(*D*) value for pHDock (−0.05) is lower than RosettaDock (−0.02) over the complete dataset. The average σ(*D*) values for pHDock (0.07) and RosettaDock (0.07) are similar, approximately 4% of the observed µ(*D*) value range. The distribution curves are generated after independent sorting of the pHDock and RosettaDock targets based on increasing *D* values.
(TIF)

S3 Figure **pHDock models containing nonstandard residue protonation states.** Number of near-native (Irmsd <4 Å) (black) and non-native (Irmsd>4 Å) (grey) pHDock models containing nonstandard residue protonation states (protonated Asp, Glu, His; deprotonated Tyr, Lys) for each target complex in the curated docking benchmark dataset. For almost all pHDock target complexes (160/161), at least one non-native model exhibits a nonstandard protonation state, while for approximately 4/5 of the complexes (127/161), at least one near-native model has nonstandard residue protonation states. The complexes are sorted based on the crystallization pH.
(TIF)

S4 Figure **Nonstandard residue protonation states in pHDock models.** (A) Number of ionizable residues exhibiting nonstandard protonation states in pHDock models for each target complex. The number of recovered nonstandard residue protonation states (compared to the protonation state in the native bound complex) in (B) near-native and (C) non-native pHDock models are also shown. The complexes are sorted based on the crystallization pH. A majority of the nonstandard residue protonation states are observed in complexes with docking pH within one pH unit of the residue intrinsic p*K*_a_ values (Asp 50%, Glu 78%, His 59%, Tyr 53%, Lys 70%). Only a small fraction of all the pHDock-generated nonstandard protonation states (Asp 17%, Glu 30%, His 70%, Tyr 34%, Lys 66%) are recovered nonstandard residue protonation states that are also observed in the native bound complex.
(TIF)

S5 Figure **Summary of pHDock performance highlighting cases with nonstandard protonation states.** Correlation plot comparing discrimination scores of pHDock and RosettaDock docking predictions for each target in the complete benchmark dataset. This plot is the same as Fig. 3 in main manuscript. However, here, grey, orange and red points represent complexes where top-ranked pHDock models contain no nonstandard protonation states, recovered nonstandard protonation states found in the native bound complex, and nonstandard protonation states not observed in the bound complex, respectively. Complexes docked at acidic pH (pH≤7.0) and basic pH (pH>7.0) are represented as circles and squares, respectively. The discrimination score cutoffs for a successful prediction (*D*<0) are marked using broken lines. Corner numbers indicate the total predictions in each plot section (edges defined by the broken lines and the solid line at 45°). Overall, pHDock outperforms RosettaDock in 67% (20/30) of the cases where the top-ranked pHDock model recovers a nonstandard protonation state observed in the native bound complex. pHDock also performs better than RosettaDock in 64% (7/11) of the cases where the top-ranked pHDock produces a nonstandard protonation state different from the one observed in the native bound complex illustrating the importance of dynamic protonation states.
(TIF)

S6 Figure **Quality of models sampled during docking.** Kernel density estimate curves of the number of high-, medium-, and acceptable-quality models sampled by pHDock and RosettaDock during a docking run generating 1000 models. Numbers in the parentheses in the legends are the average number of the various quality models sampled by the docking algorithms.
(TIF)

S7 Figure ***N*** **_5_ distributions for RosettaDock and pHDock algorithms.** Mean *N*_5_ (µ(*N*_5_)) values obtained from bootstrap case resampling of the docking models (1000 models per target with replacement) for pHDock (orange) and RosettaDock (grey). Standard deviations (σ(*N*_5_)) are represented as error margins. The average µ(*N*_5_) values for pHDock (2.60) and RosettaDock (2.55) are similar over the complete dataset. The average σ(*N*_5_) values are high for both pHDock (0.65) and RosettaDock (0.65), approximately 13% of the observed µ(*N*_5_) range, indicating significant inherent noise using the *N*_5_ metric for the set of models. The distribution curves are generated after independent sorting of the pHDock and RosettaDock targets based on decreasing *N*_5_ values.
(TIF)

S8 Figure **Docking plots for pHDock and RosettaDock.** Grey, orange, red, and blue points represent incorrect, acceptable-, medium-, and high-quality predictions, respectively. Discrimination scores are shown in the bottom right corner of the plots.
(PDF)

S9 Figure **Docking plots for pHDock and RosettaDock highlighting models with nonstandard residue protonation states.** Grey, orange and red points represent models containing no nonstandard protonation states, recovered nonstandard protonation states found in the bound complex, and nonstandard protonation states not observed in the bound complex, respectively. Discrimination scores are shown in the bottom right corner of the plots.
(PDF)

S10 Figure **Docking plots comparing ensemble pHDock to pHDock and RosettaDock.** Grey, orange, and red points represent incorrect, acceptable-, and medium- quality predictions, respectively. Discrimination scores are shown in the bottom right corner of the plots.
(PDF)

S11 Figure **Docking plots comparing RosettaDock, pHDock at crystallization pH, pHDock at pH 7.0 and FixpHDock.** Grey, orange and red points represent models containing no nonstandard residue protonation states, recovered nonstandard residue protonation states found in the bound complex, and nonstandard residue protonation states not observed in the bound complex, respectively. In FixpHDock, the protonation states found in the starting unbound complex are held constant during docking. Discrimination scores are shown in the bottom right corner of the plots.
(PDF)

S1 Table **Score functions used for the study.** Weights for the score terms used during residue side-chain and protonation state sampling, receptor-ligand minimization, and for ranking final docking models.
(PDF)

S2 Table **Docking performance summary.** PDB IDs and pH values of the benchmark dataset used for the study. Discrimination scores, *N*_5_ values, Irmsd and *f*_nat_ of the lowest-Irmsd and top-ranked models generated using pHDock and RosettaDock are also listed.
(PDF)

S3 Table **Ensemble pHDock performance summary.** PDB IDs and pH values of the benchmark subset used for ensemble pHDock. Discrimination scores, Irmsd, *f*_nat_, and number of recovered native interface hydrogen bonds in the top-ranked models generated using ensemble pHDock are compared to pHDock and RosettaDock.
(PDF)

S1 Text **Command lines for pHDock, RosettaDock and ensemble pHDock.**
(PDF)
