# Supplementary figures and images for: Protein-Protein Docking with Dynamic Residue Protonation States
Source: PLoS Comput Biol. 2014 Dec 11;10(12):e1004018. doi: 10.1371/journal.pcbi.1004018 (PMC4263365; doi:10.1371/journal.pcbi.1004018)

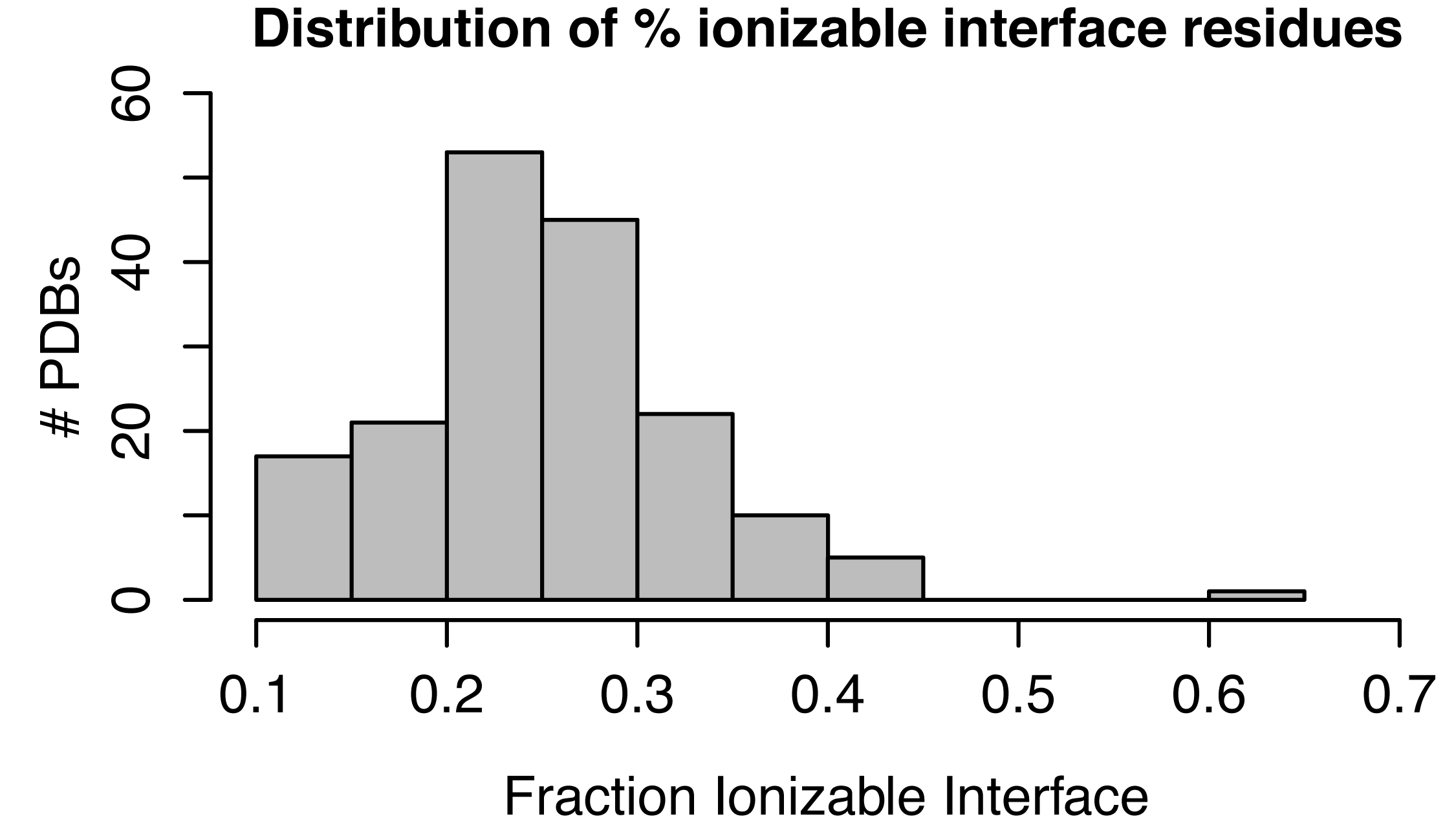

Supplement: S1 Figure — Distribution of ionizable residues at docking interfaces. Frequency histogram of the number of dataset complexes with various fractions of ionizable interface residues (Asp, Glu, His, Tyr, Lys). (TIF) [file pcbi.1004018.s001.tif]

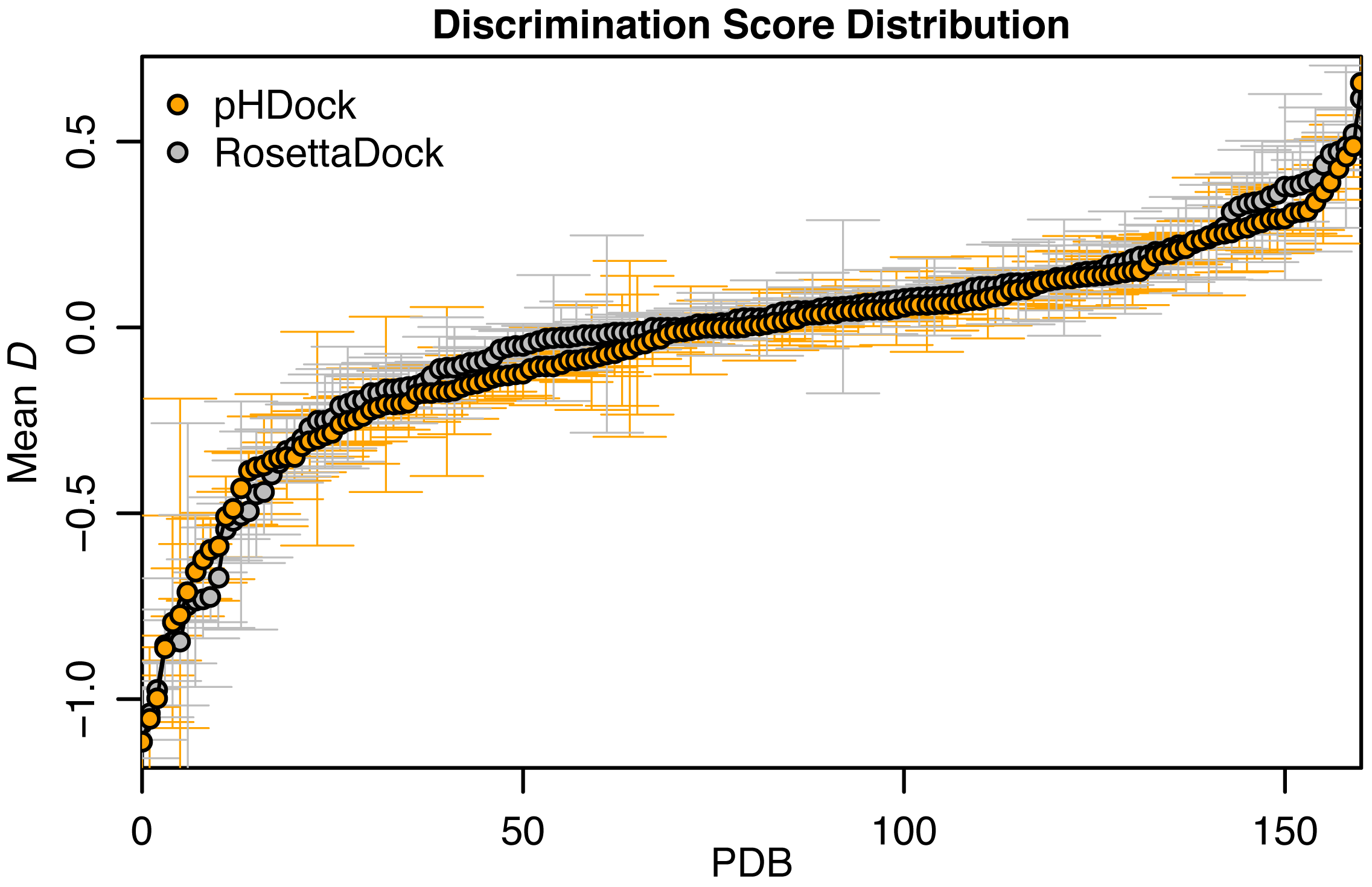

Supplement: S2 Figure — Discrimination score ( D ) distributions for RosettaDock and pHDock algorithms. Mean D (µ(D)) values obtained from bootstrap case resampling of the docking models (1000 models per target with replacement) for pHDock (orange) and RosettaDock (grey). Standard deviations (σ(D)) are represented as error margins. The average µ(D) value for pHDock (−0.05) is lower than RosettaDock (−0.02) over the complete dataset. The average σ(D) values for pHDock (0.07) and RosettaDock (0.07) are similar, approximately 4% of the observed µ(D) value range. The distribution curves are generated after independent sorting of the pHDock and RosettaDock targets based on increasing D values. (TIF) [file pcbi.1004018.s002.tif]

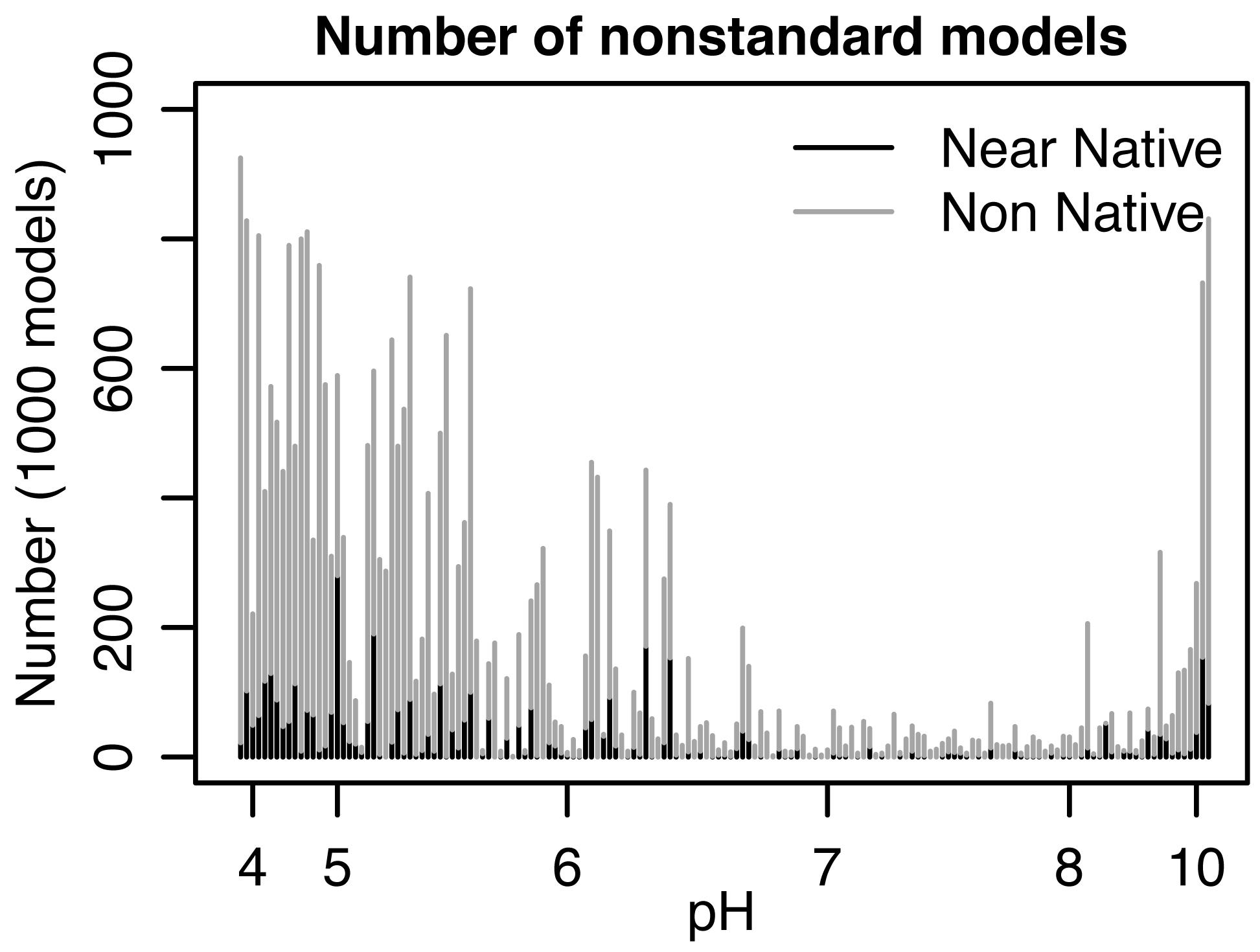

Supplement: S3 Figure — pHDock models containing nonstandard residue protonation states. Number of near-native (Irmsd <4 Å) (black) and non-native (Irmsd>4 Å) (grey) pHDock models containing nonstandard residue protonation states (protonated Asp, Glu, His; deprotonated Tyr, Lys) for each target complex in the curated docking benchmark dataset. For almost all pHDock target complexes (160/161), at least one non-native model exhibits a nonstandard protonation state, while for approximately 4/5 of the complexes (127/161), at least one near-native model has nonstandard residue protonation states. The complexes are sorted based on the crystallization pH. (TIF) [file pcbi.1004018.s003.tif]

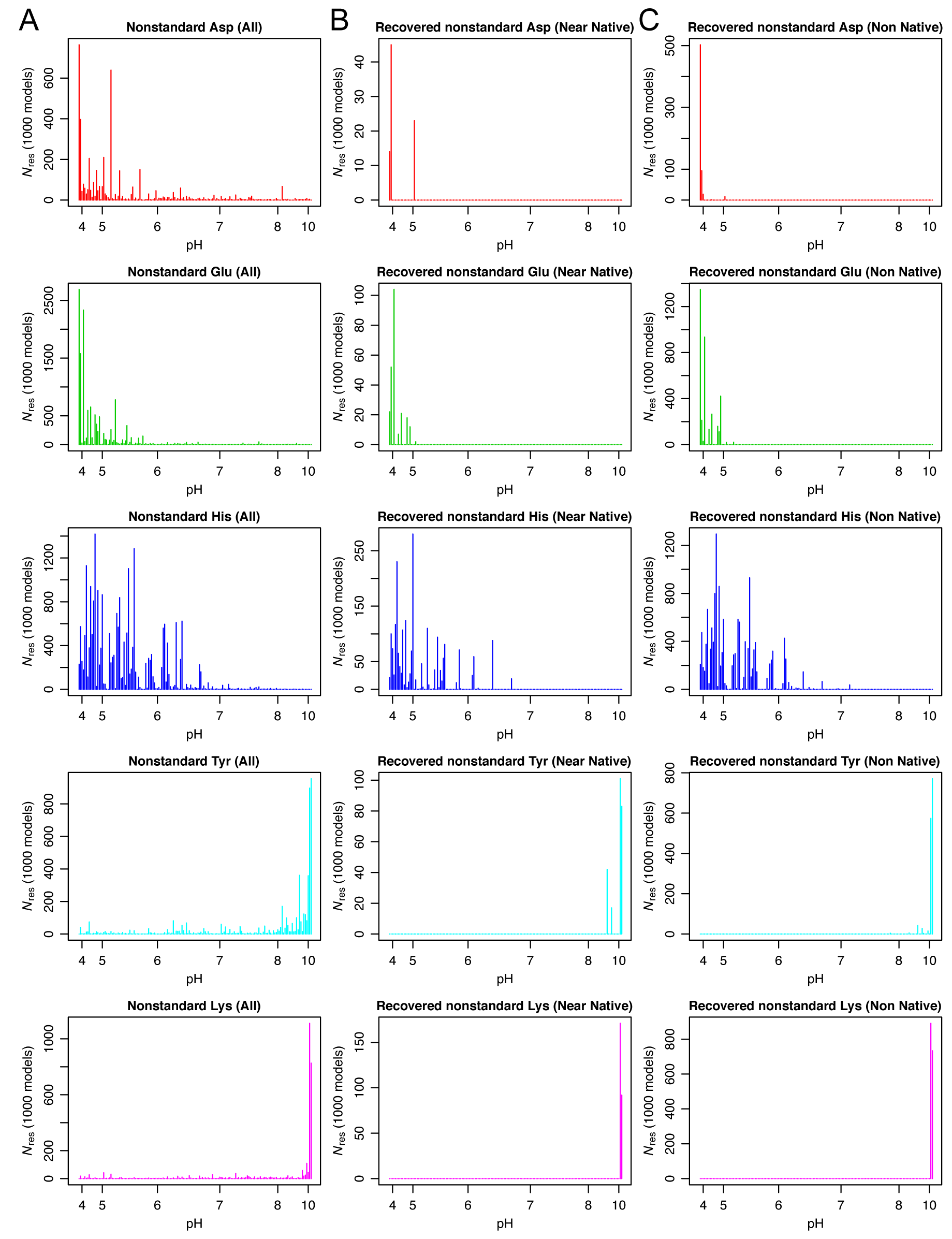

Supplement: S4 Figure — Nonstandard residue protonation states in pHDock models. (A) Number of ionizable residues exhibiting nonstandard protonation states in pHDock models for each target complex. The number of recovered nonstandard residue protonation states (compared to the protonation state in the native bound complex) in (B) near-native and (C) non-native pHDock models are also shown. The complexes are sorted based on the crystallization pH. A majority of the nonstandard residue protonation states are observed in complexes with docking pH within one pH unit of the residue intrinsic pK a values (Asp 50%, Glu 78%, His 59%, Tyr 53%, Lys 70%). Only a small fraction of all the pHDock-generated nonstandard protonation states (Asp 17%, Glu 30%, His 70%, Tyr 34%, Lys 66%) are recovered nonstandard residue protonation states that are also observed in the native bound complex. (TIF) [file pcbi.1004018.s004.tif]

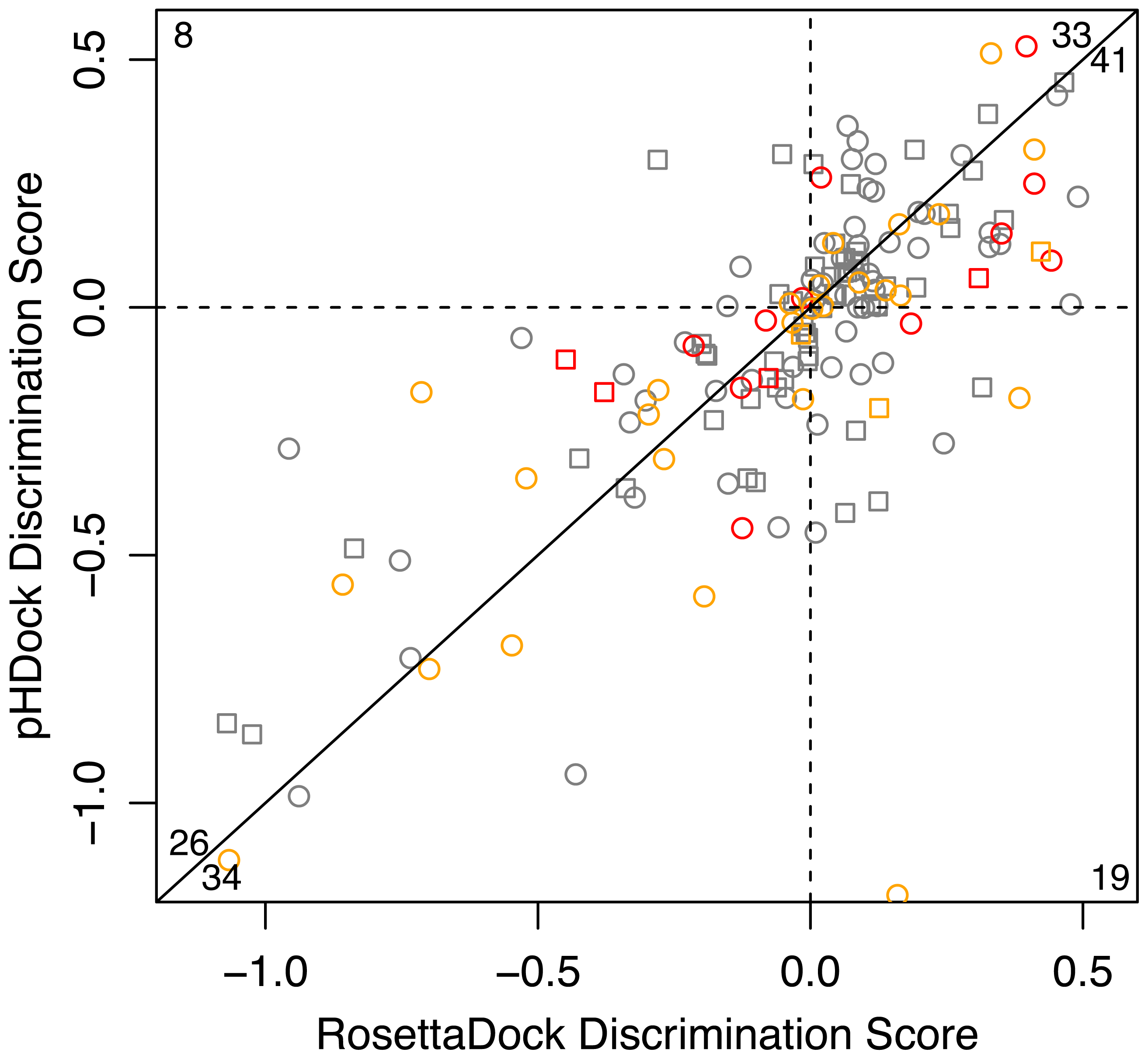

Supplement: S5 Figure — Summary of pHDock performance highlighting cases with nonstandard protonation states. Correlation plot comparing discrimination scores of pHDock and RosettaDock docking predictions for each target in the complete benchmark dataset. This plot is the same as Fig. 3 in main manuscript. However, here, grey, orange and red points represent complexes where top-ranked pHDock models contain no nonstandard protonation states, recovered nonstandard protonation states found in the native bound complex, and nonstandard protonation states not observed in the bound complex, respectively. Complexes docked at acidic pH (pH≤7.0) and basic pH (pH>7.0) are represented as circles and squares, respectively. The discrimination score cutoffs for a successful prediction (D<0) are marked using broken lines. Corner numbers indicate the total predictions in each plot section (edges defined by the broken lines and the solid line at 45°). Overall, pHDock outperforms RosettaDock in 67% (20/30) of the cases where the top-ranked pHDock model recovers a nonstandard protonation state observed in the native bound complex. pHDock also performs better than RosettaDock in 64% (7/11) of the cases where the top-ranked pHDock produces a nonstandard protonation state different from the one observed in the native bound complex illustrating the importance of dynamic protonation states. (TIF) [file pcbi.1004018.s005.tif]

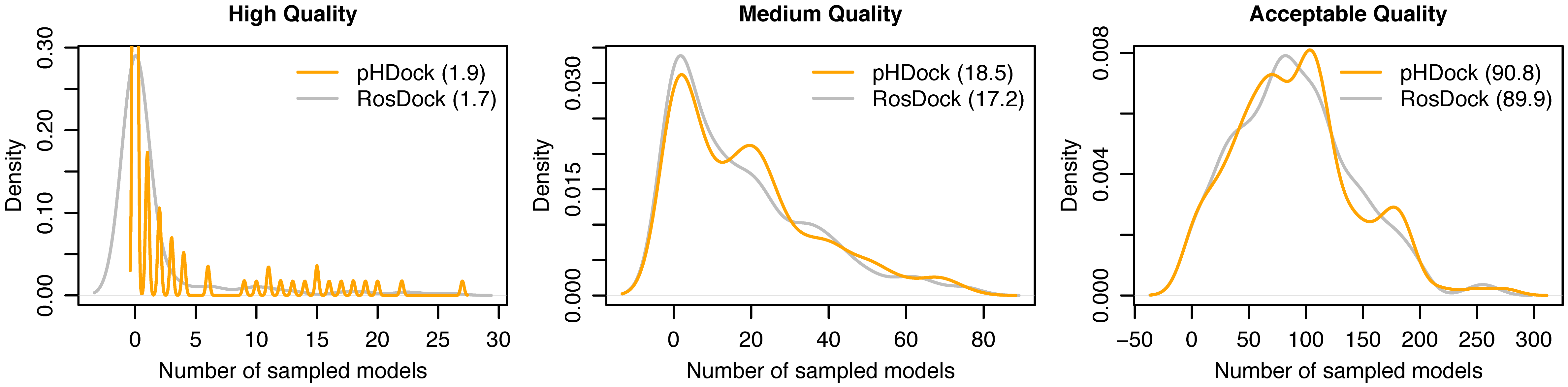

Supplement: S6 Figure — Quality of models sampled during docking. Kernel density estimate curves of the number of high-, medium-, and acceptable-quality models sampled by pHDock and RosettaDock during a docking run generating 1000 models. Numbers in the parentheses in the legends are the average number of the various quality models sampled by the docking algorithms. (TIF) [file pcbi.1004018.s006.tif]

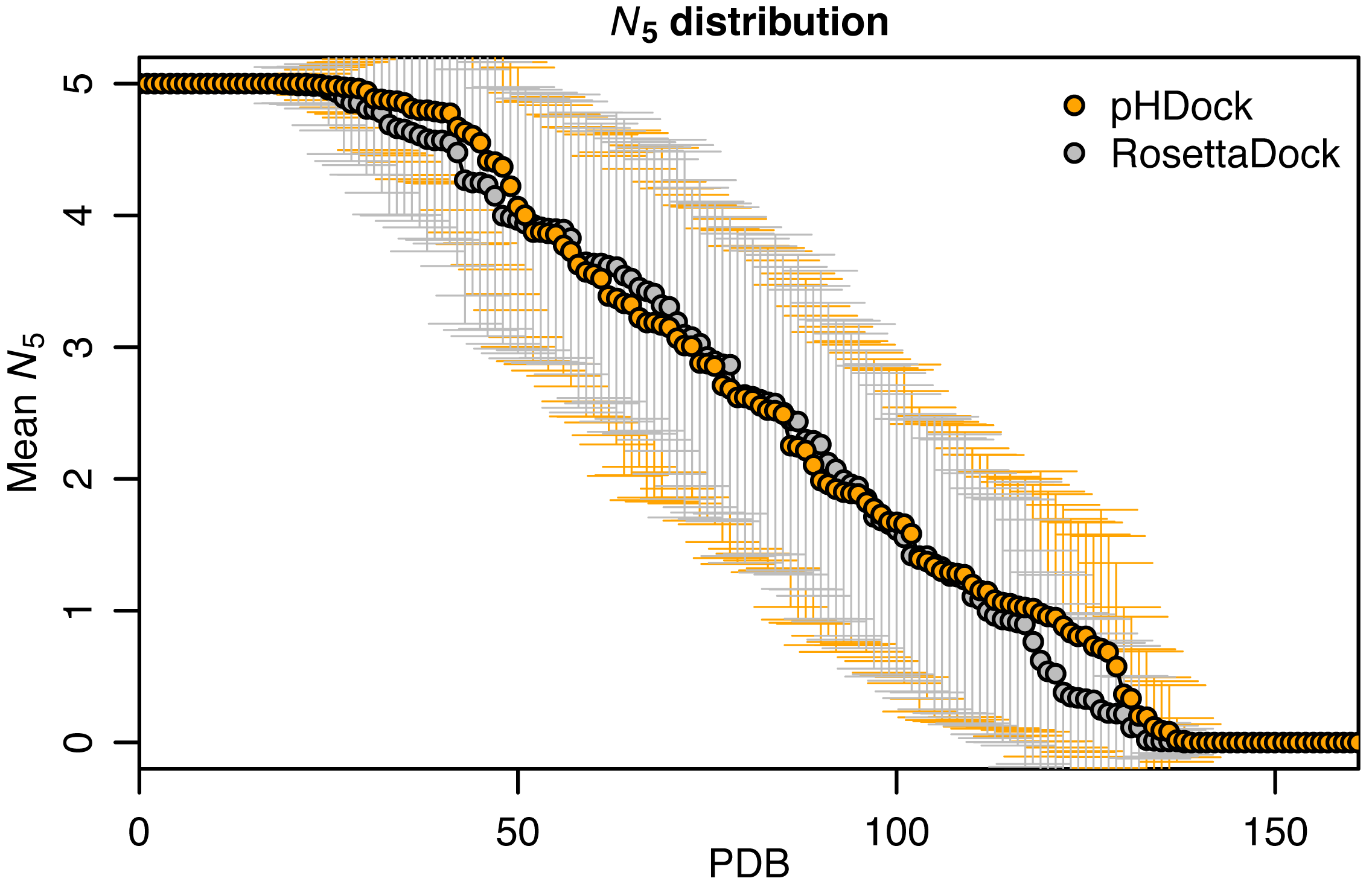

Supplement: S7 Figure — N 5 distributions for RosettaDock and pHDock algorithms. Mean N 5 (µ(N 5)) values obtained from bootstrap case resampling of the docking models (1000 models per target with replacement) for pHDock (orange) and RosettaDock (grey). Standard deviations (σ(N 5)) are represented as error margins. The average µ(N 5) values for pHDock (2.60) and RosettaDock (2.55) are similar over the complete dataset. The average σ(N 5) values are high for both pHDock (0.65) and RosettaDock (0.65), approximately 13% of the observed µ(N 5) range, indicating significant inherent noise using the N 5 metric for the set of models. The distribution curves are generated after independent sorting of the pHDock and RosettaDock targets based on decreasing N 5 values. (TIF) [file pcbi.1004018.s007.tif]

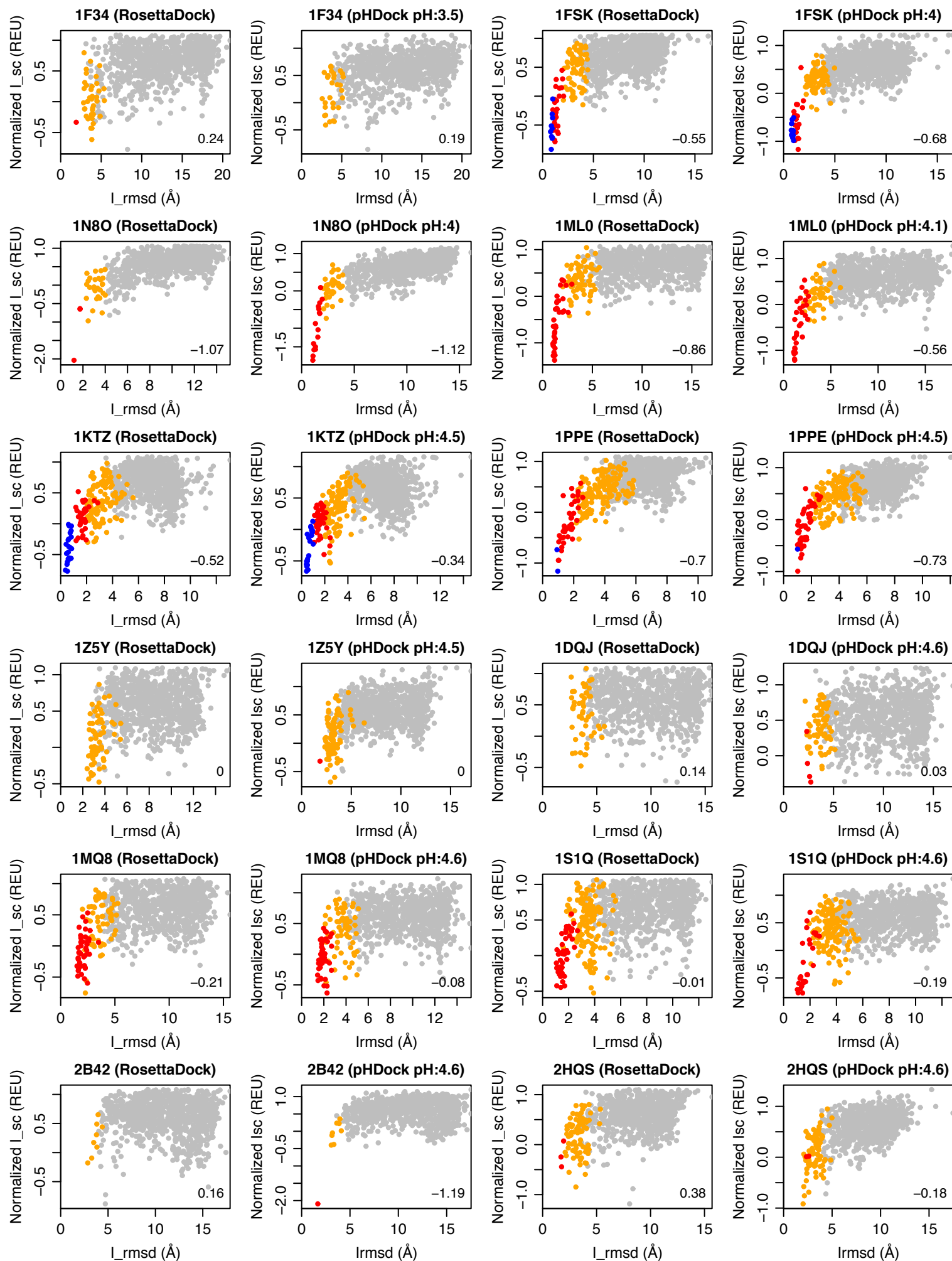

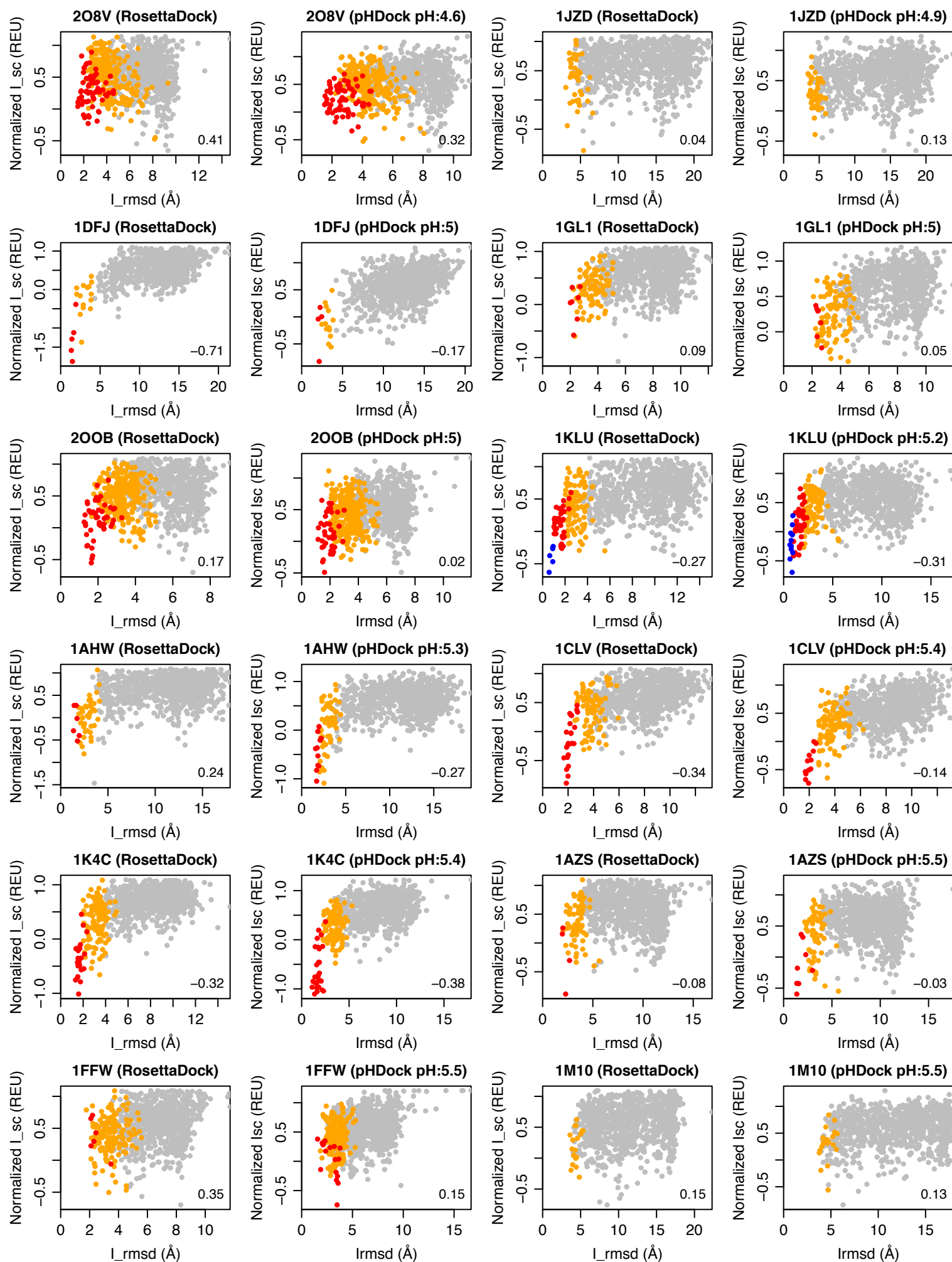

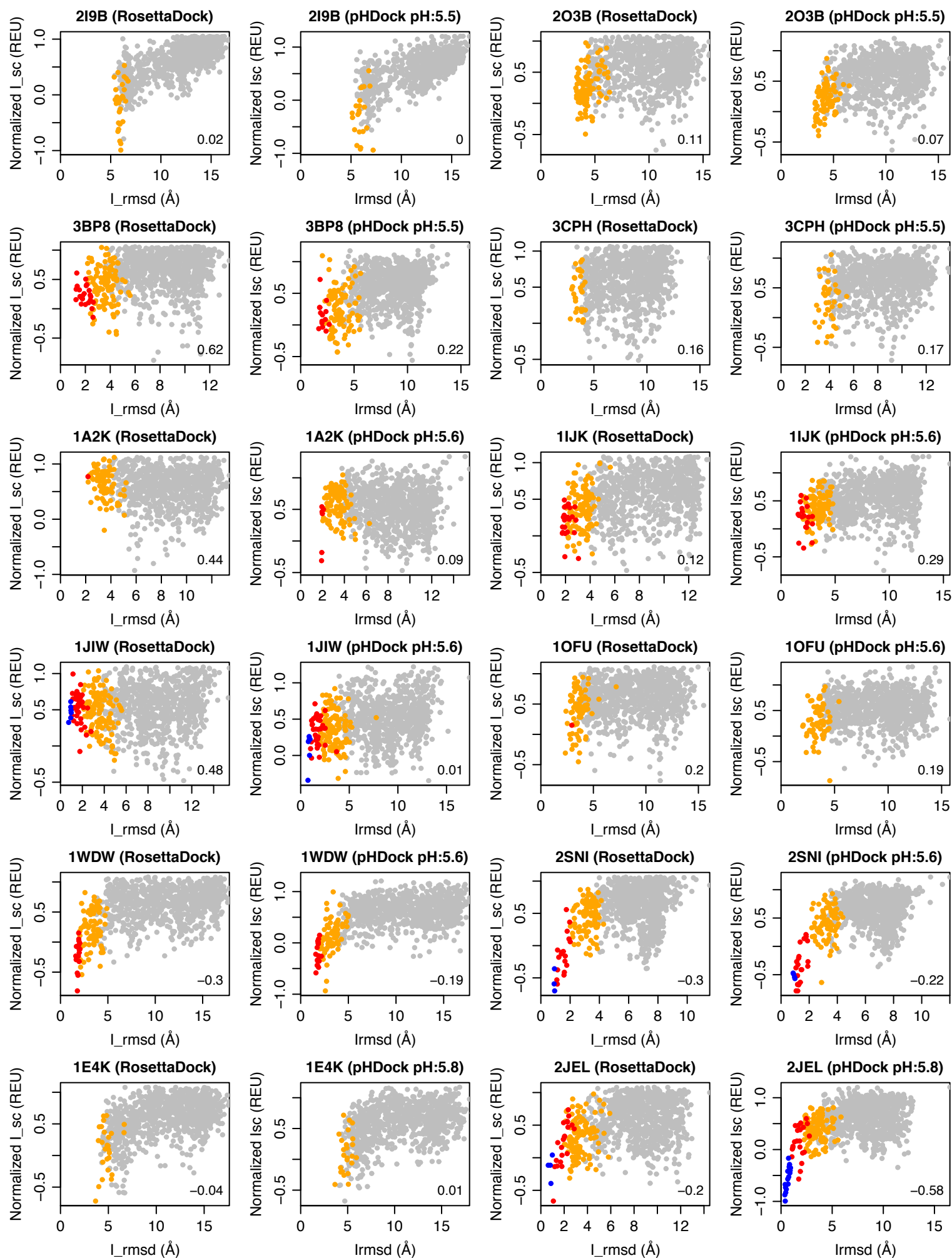

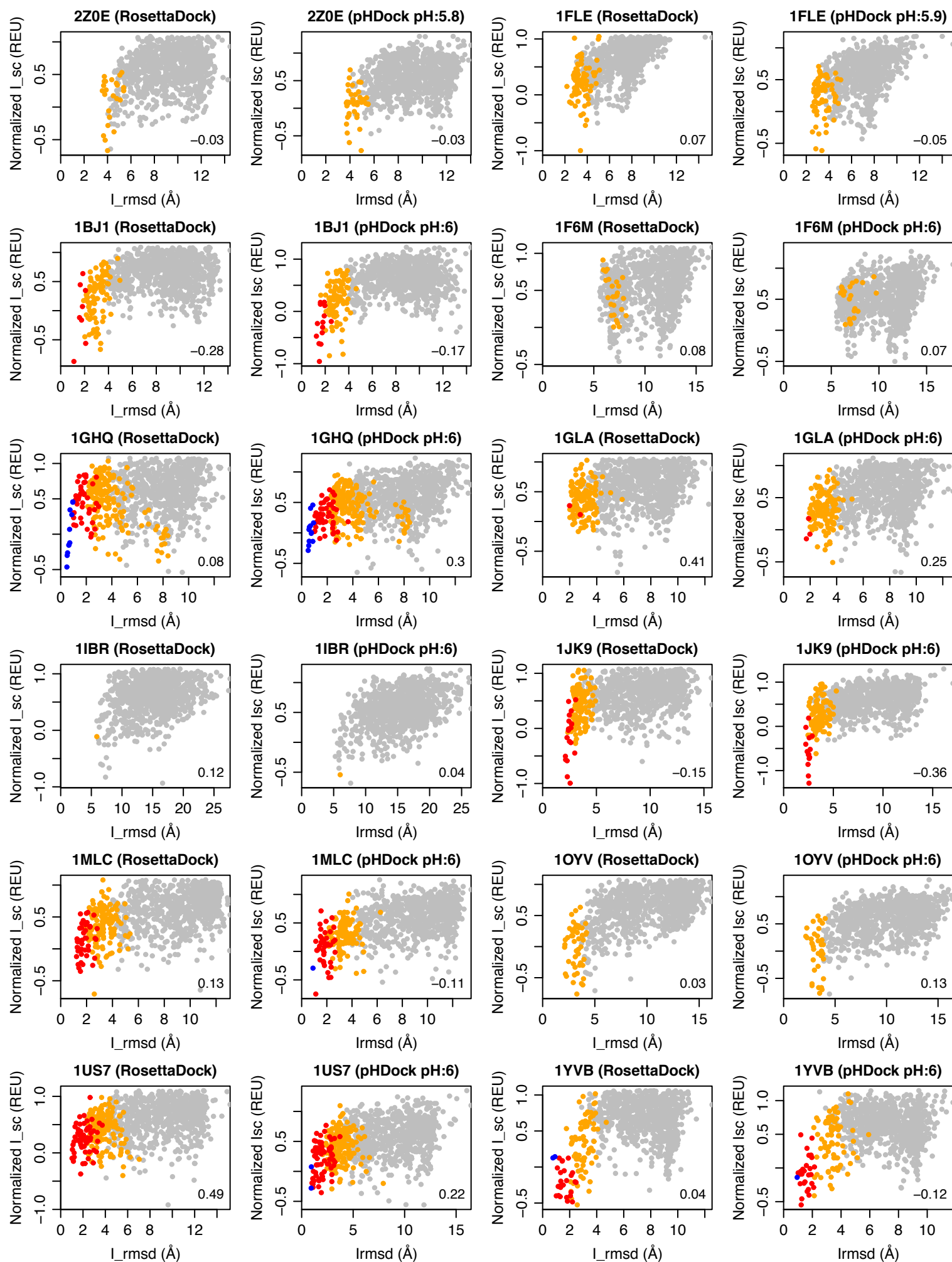

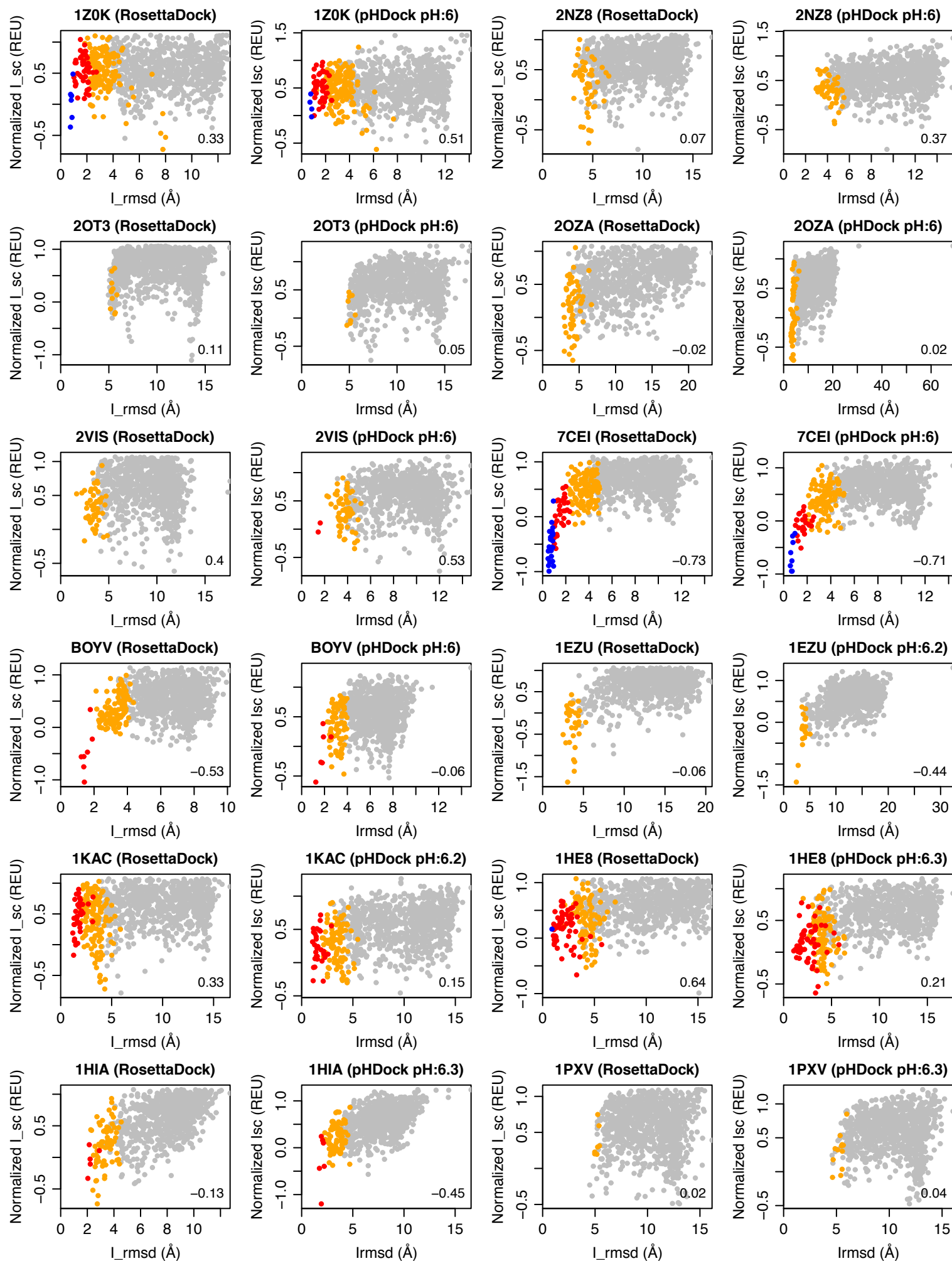

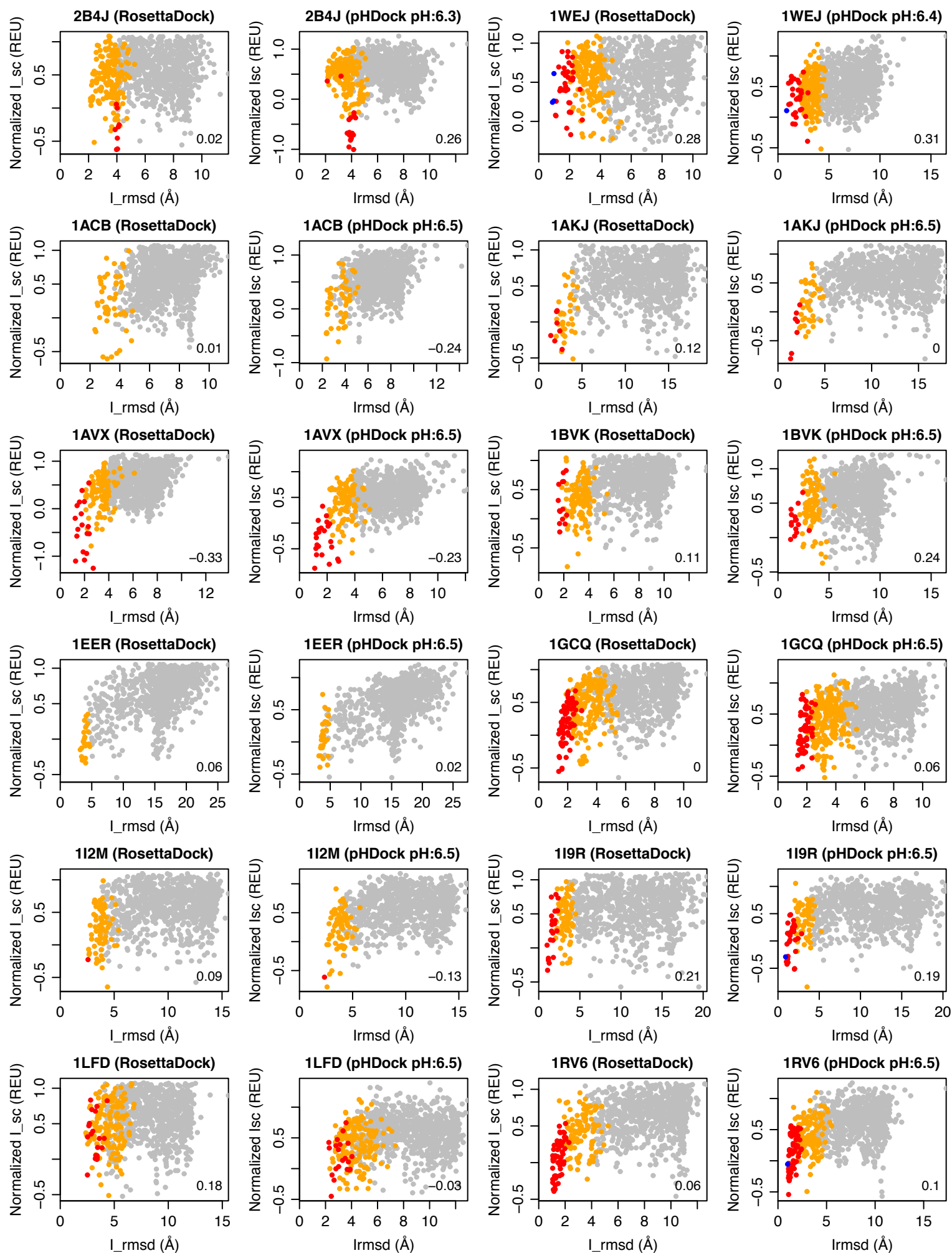

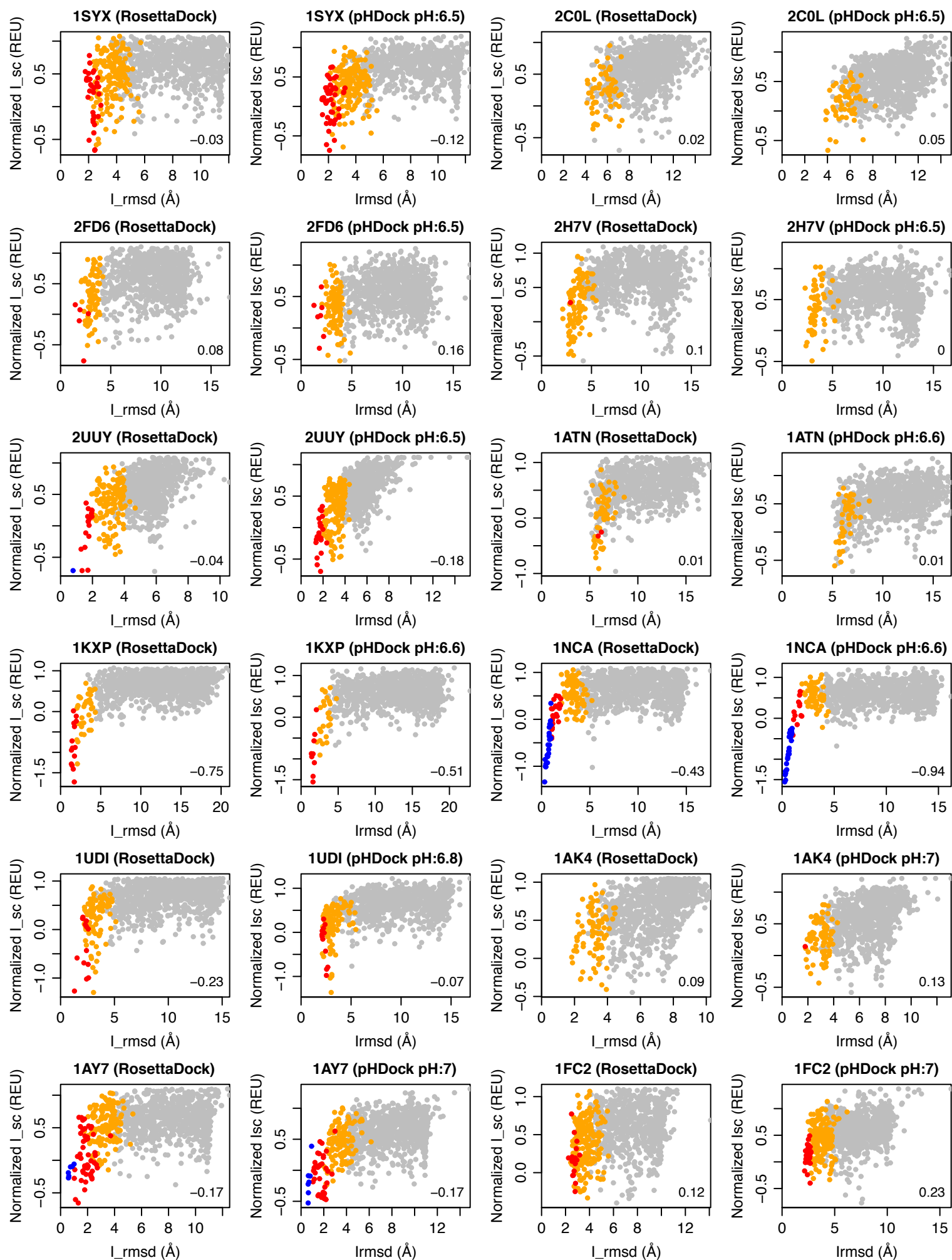

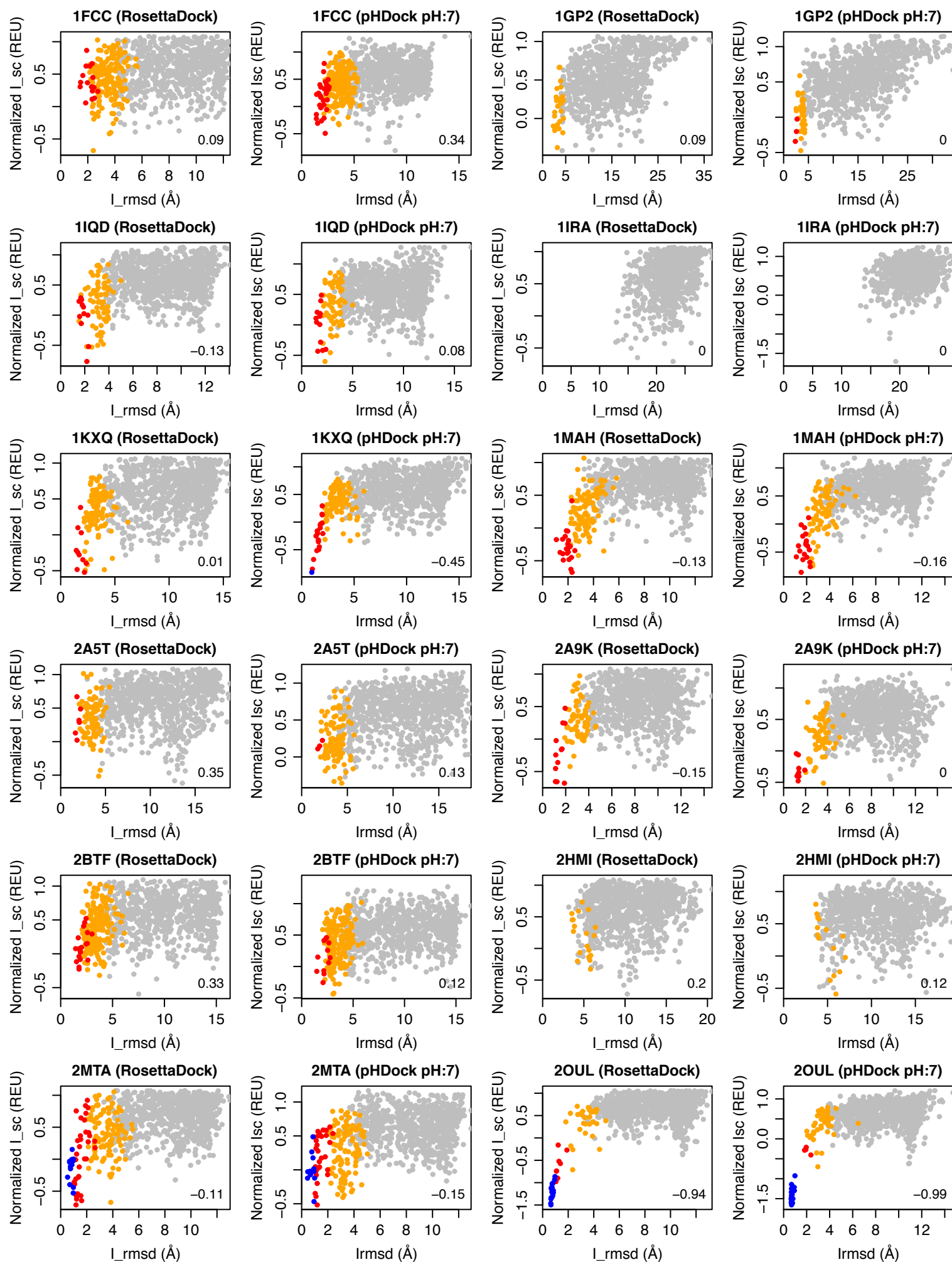

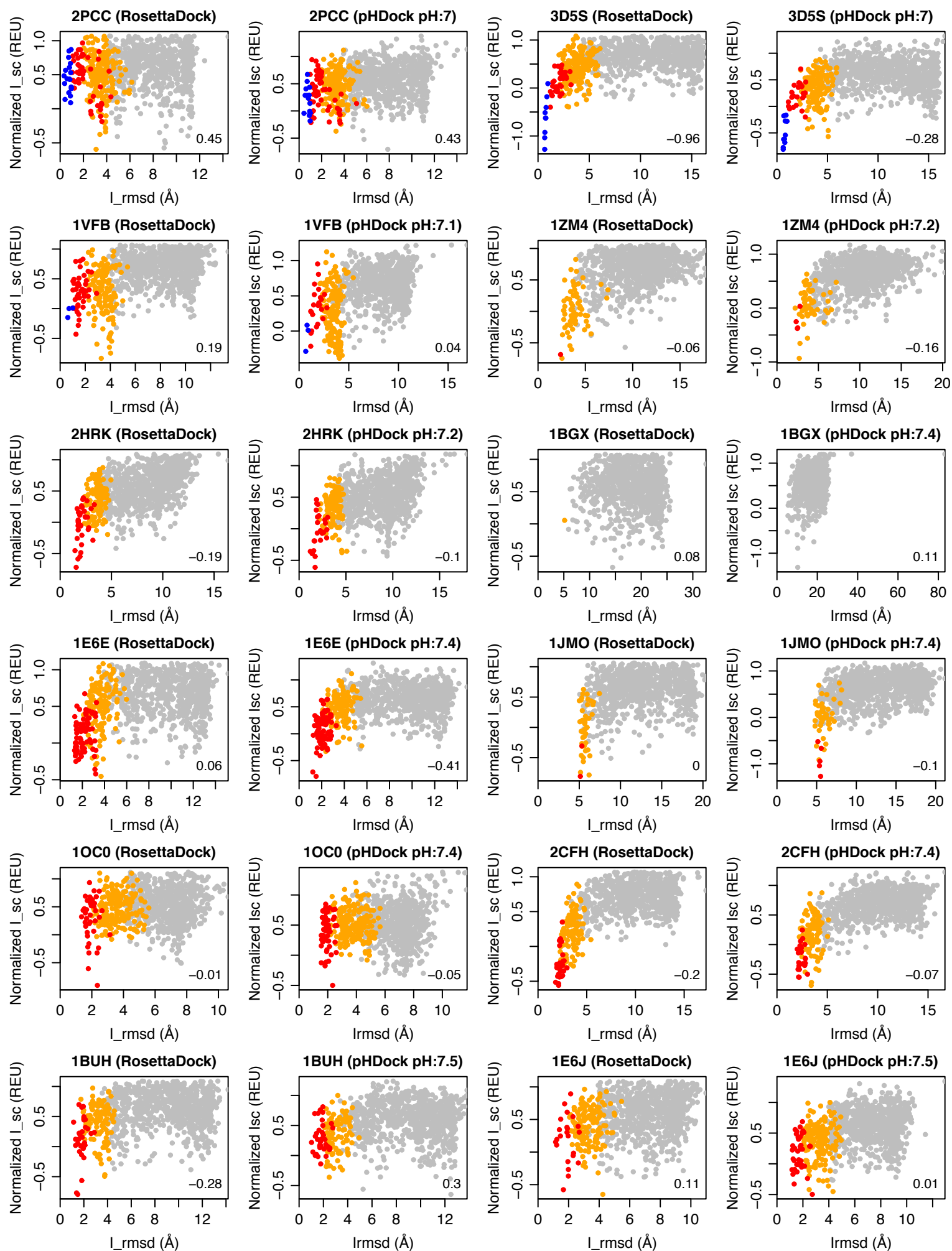

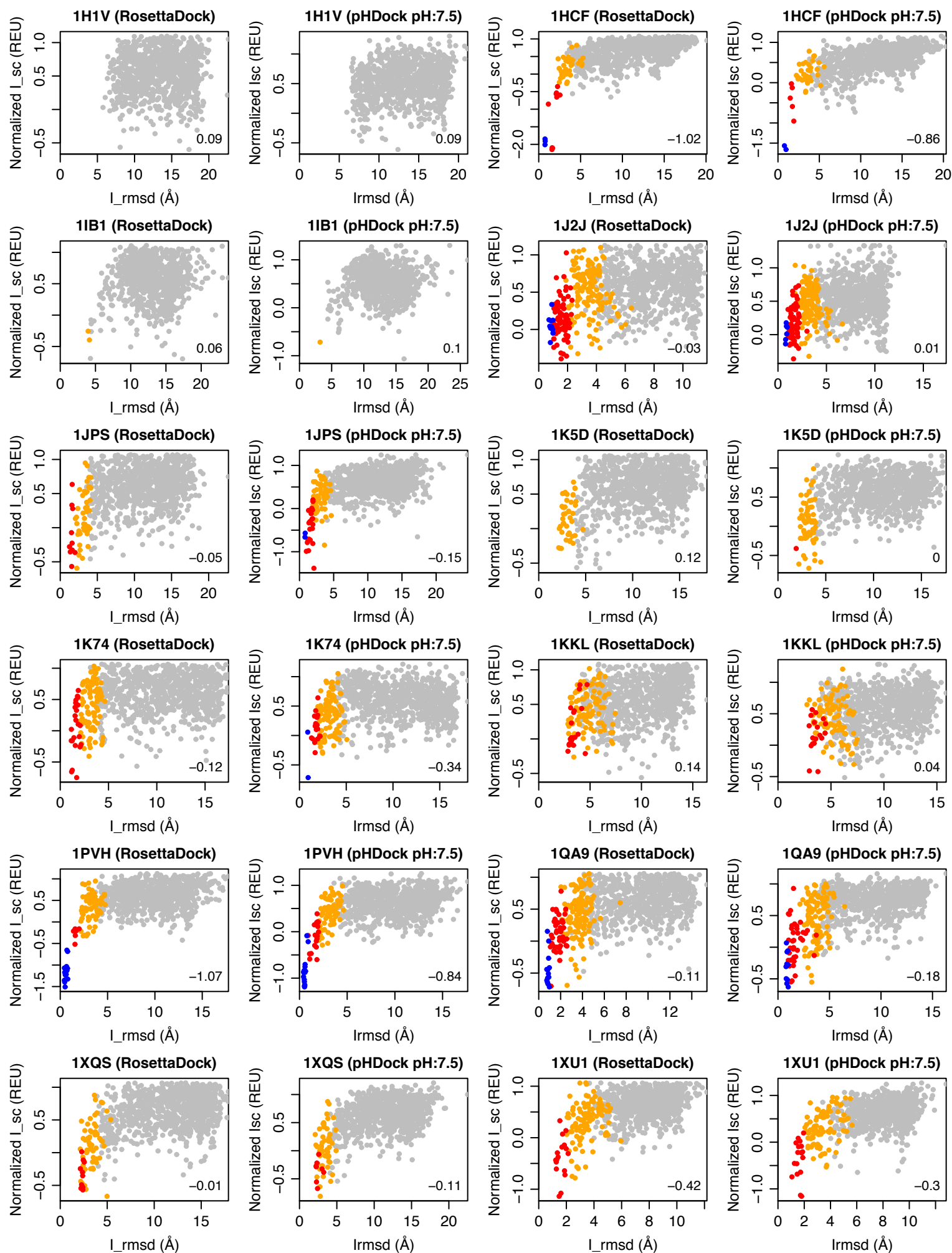

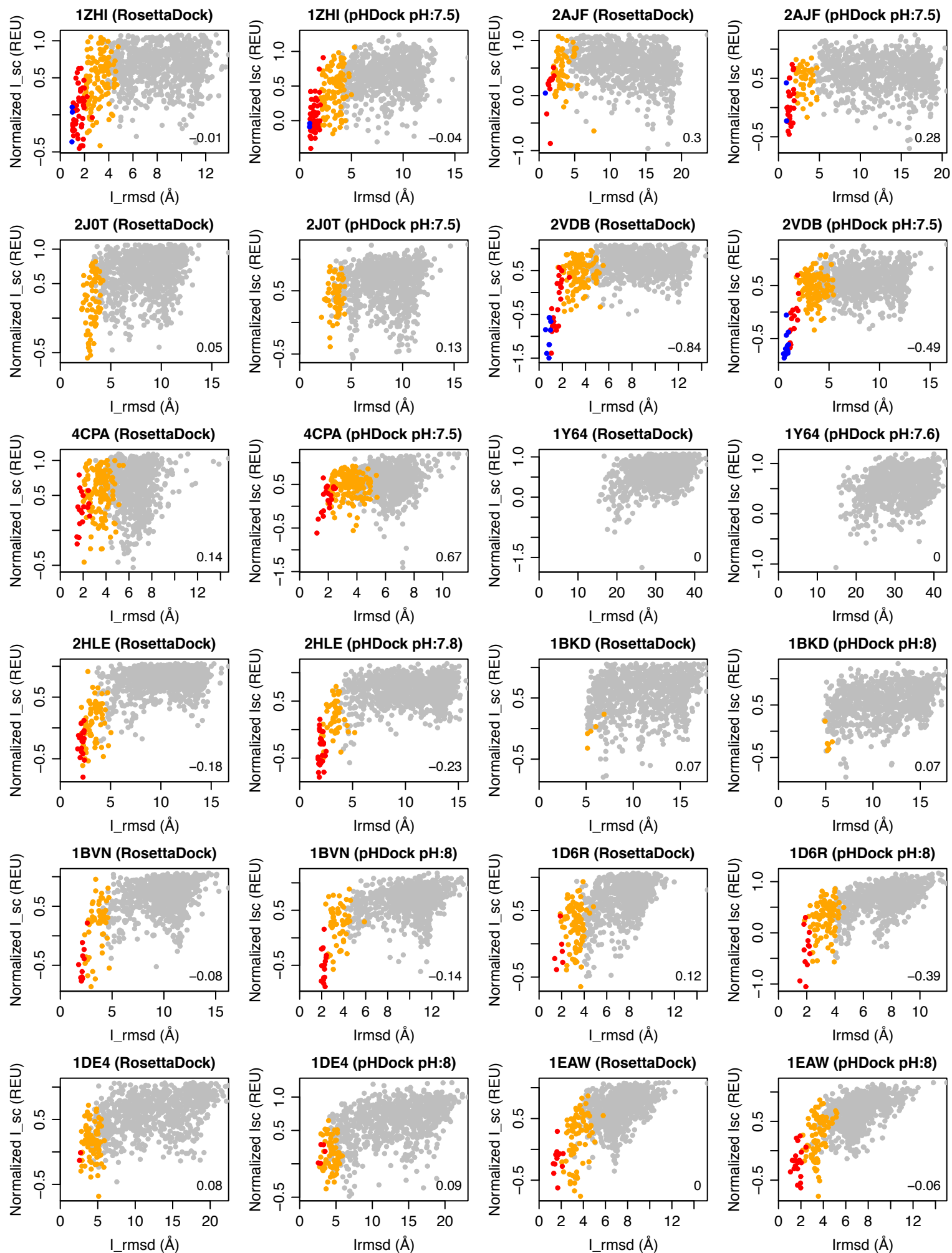

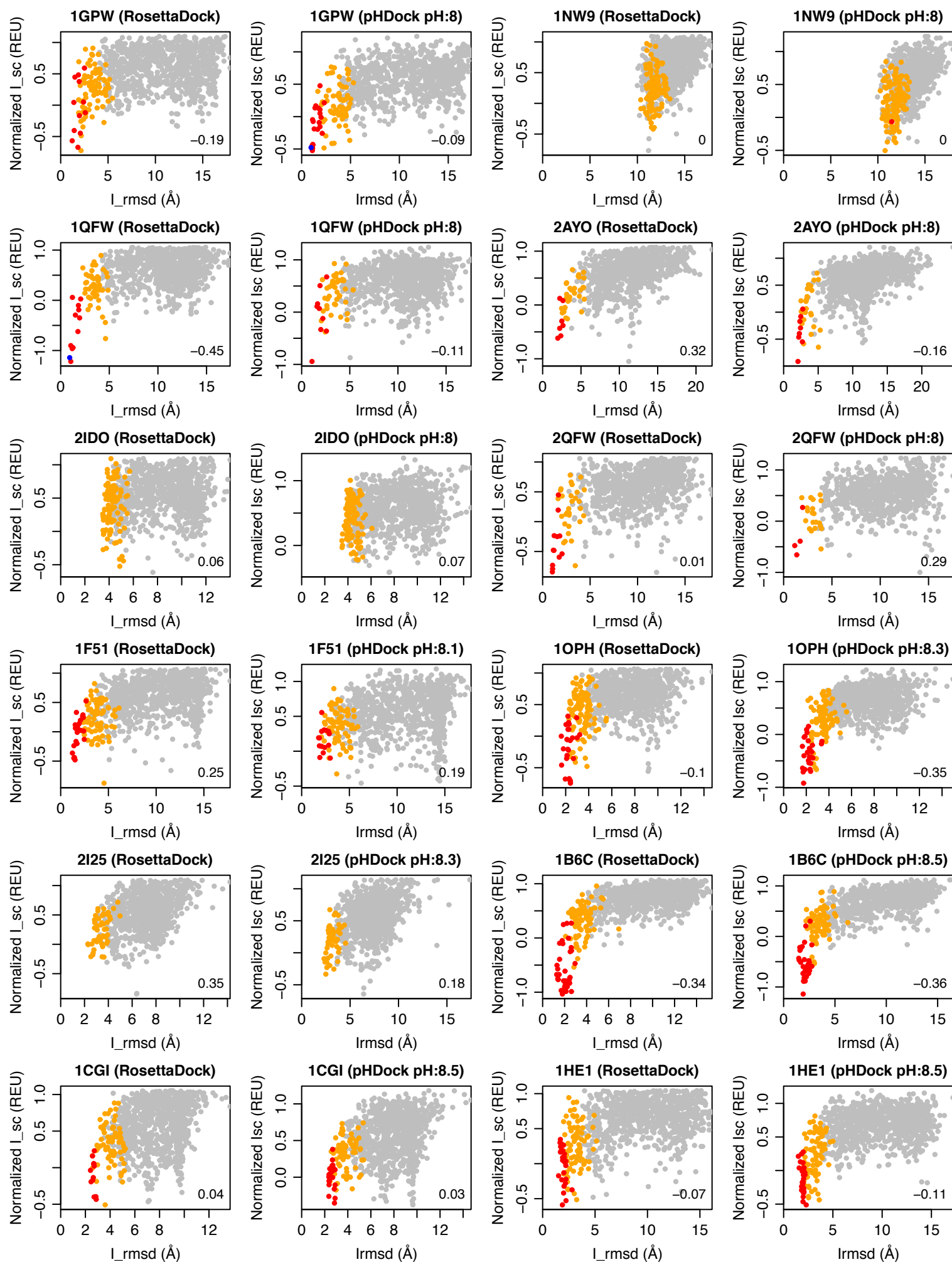

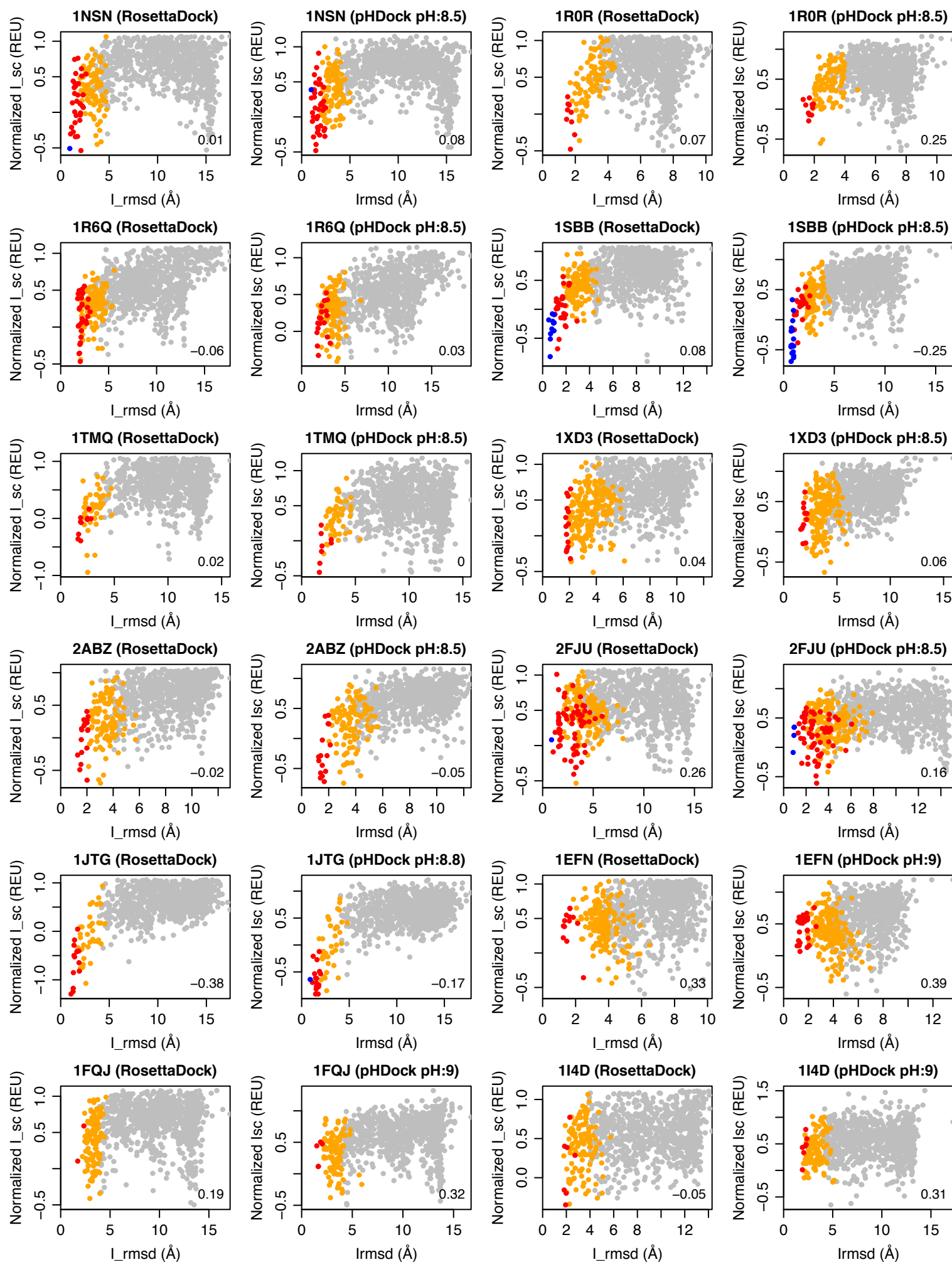

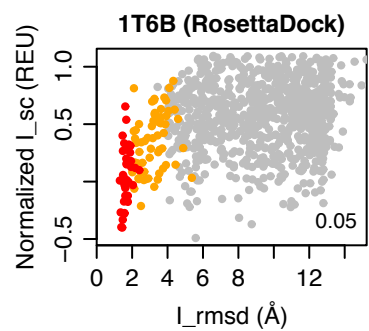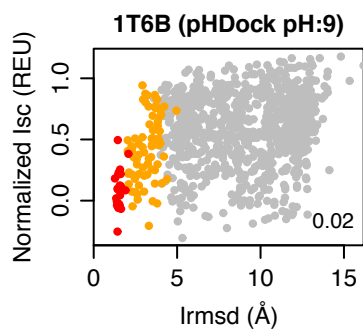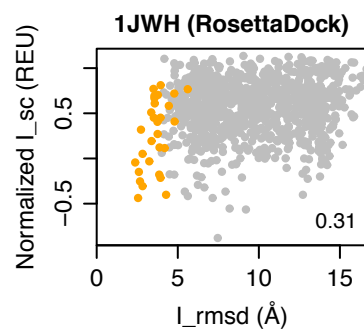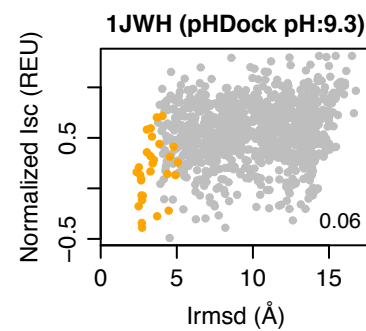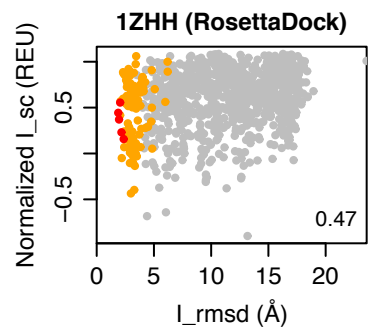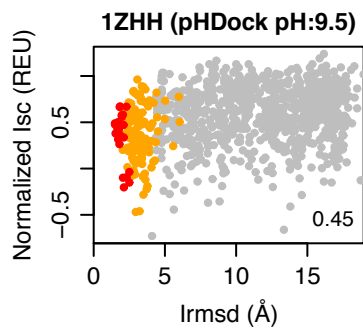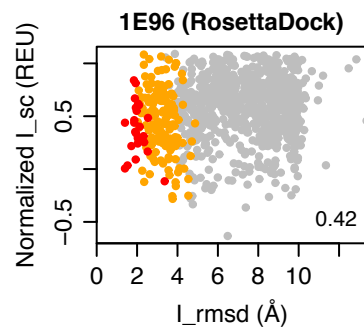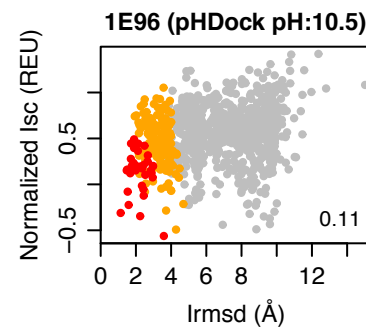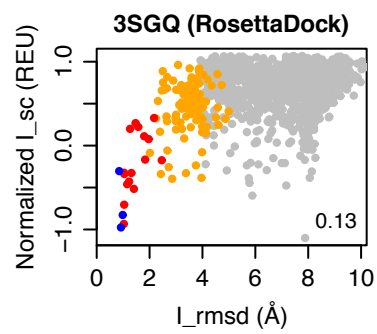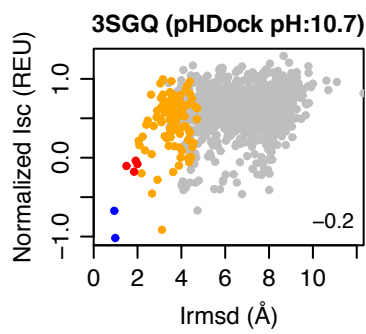

Supplement: S8 Figure — Docking plots for pHDock and RosettaDock. Grey, orange, red, and blue points represent incorrect, acceptable-, medium-, and high-quality predictions, respectively. Discrimination scores are shown in the bottom right corner of the plots. (PDF) [file pcbi.1004018.s008.pdf]

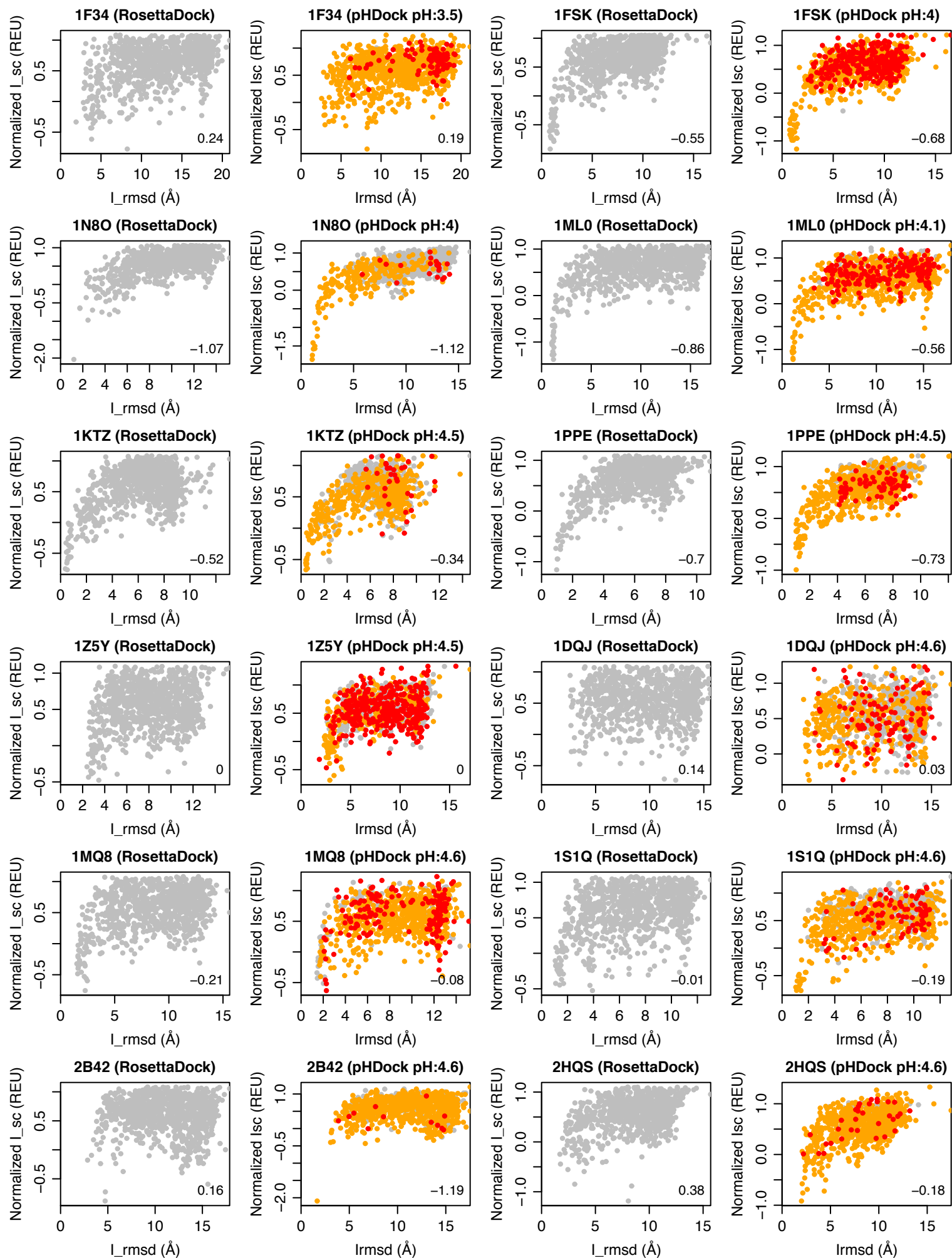

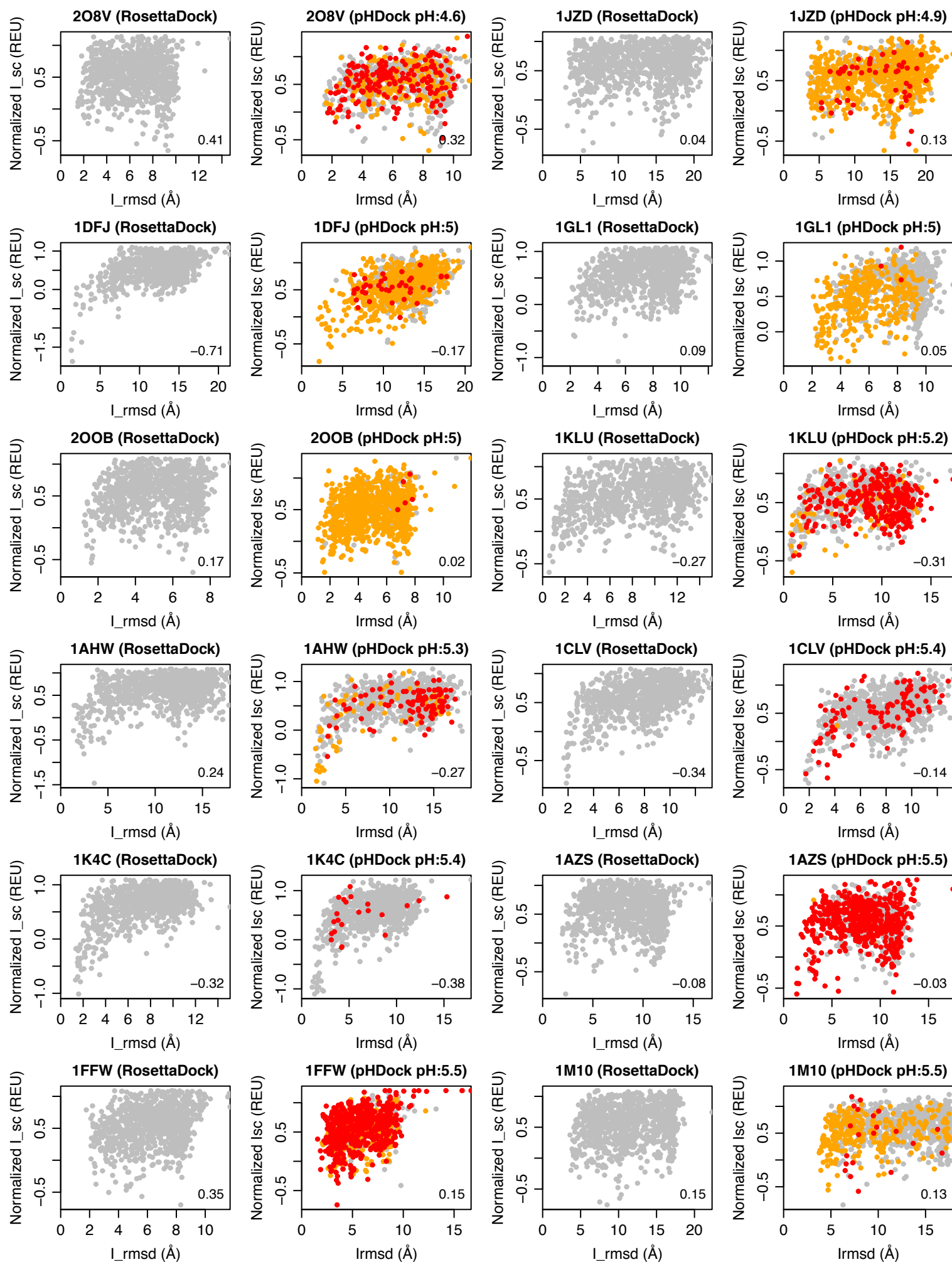

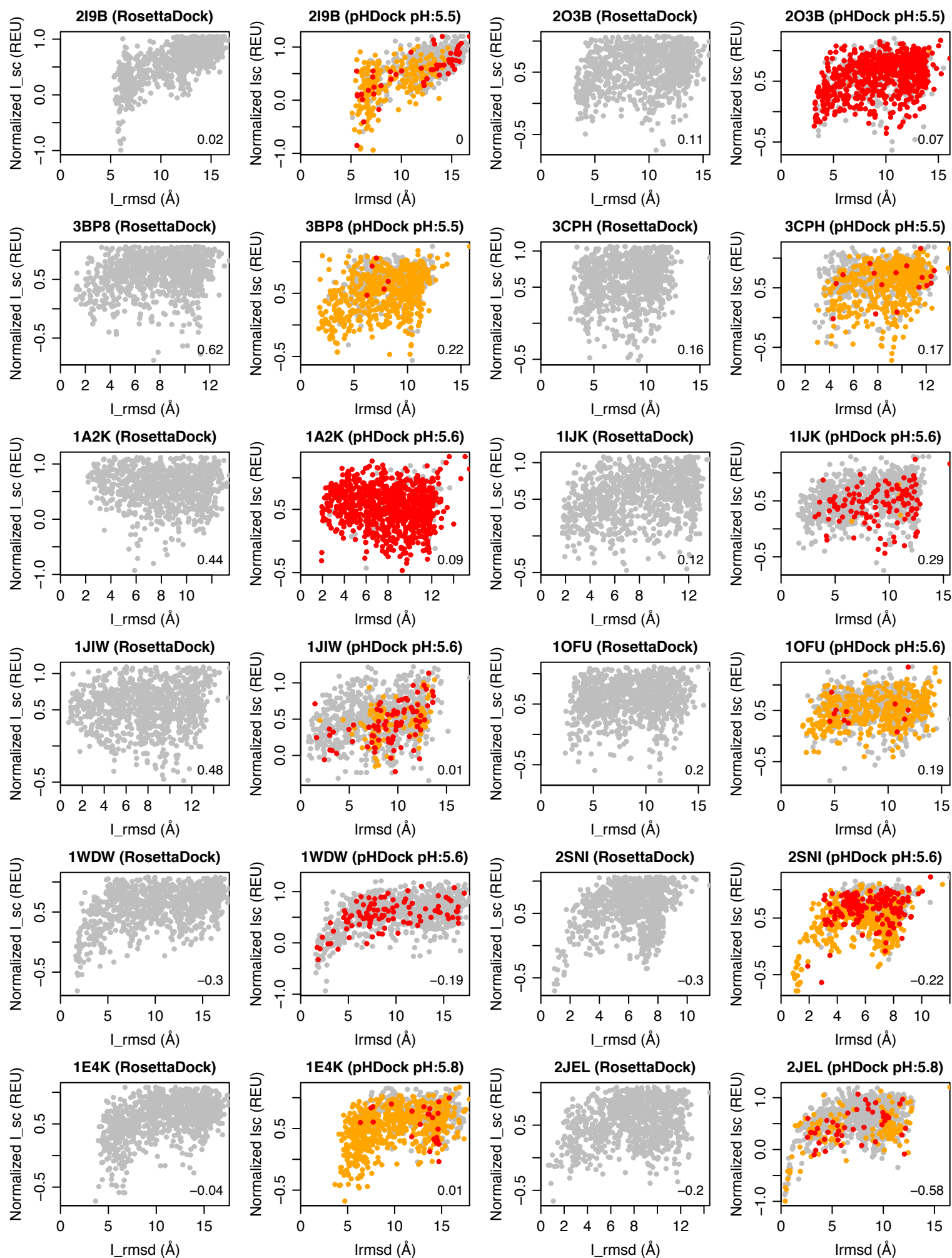

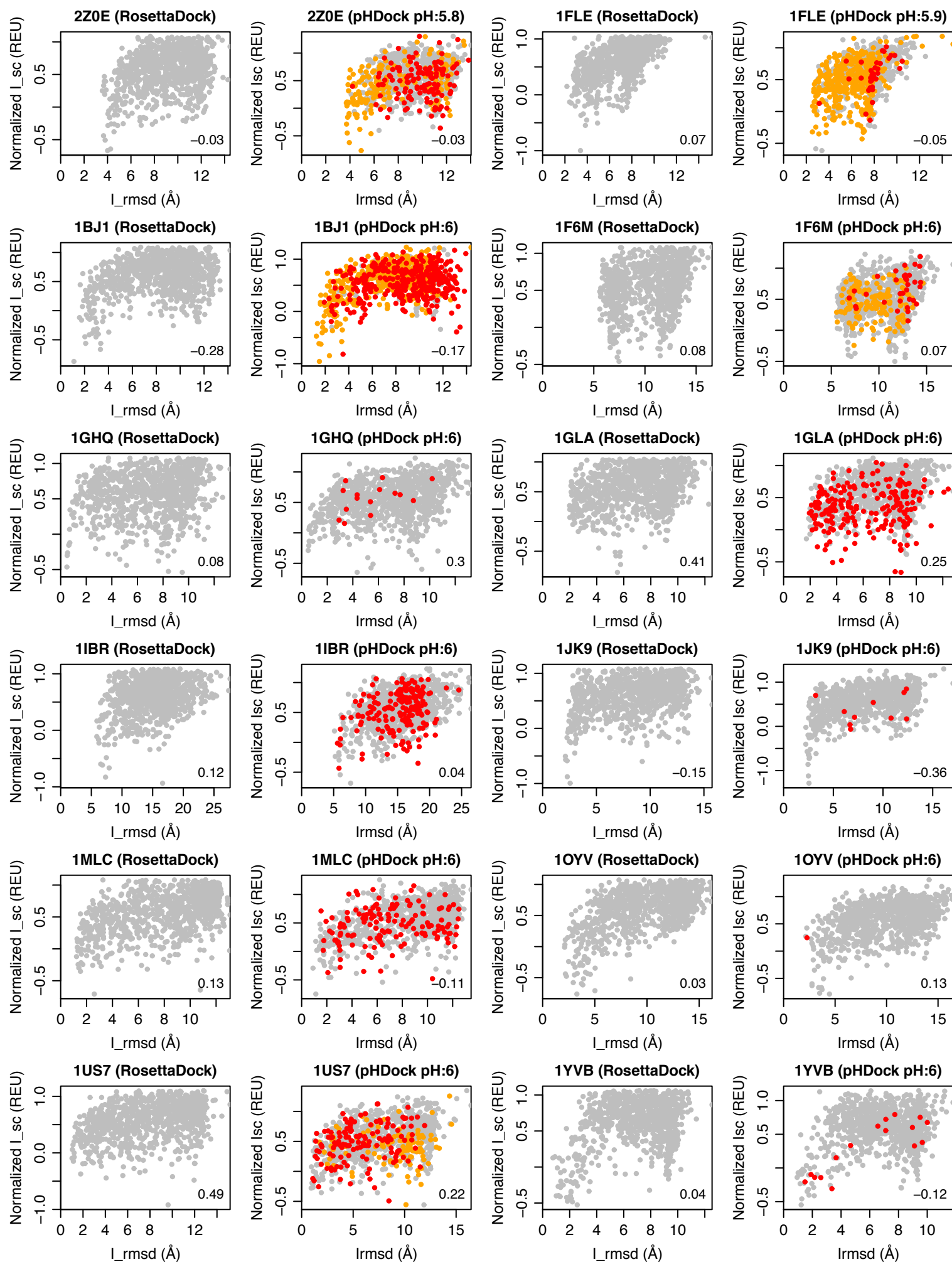

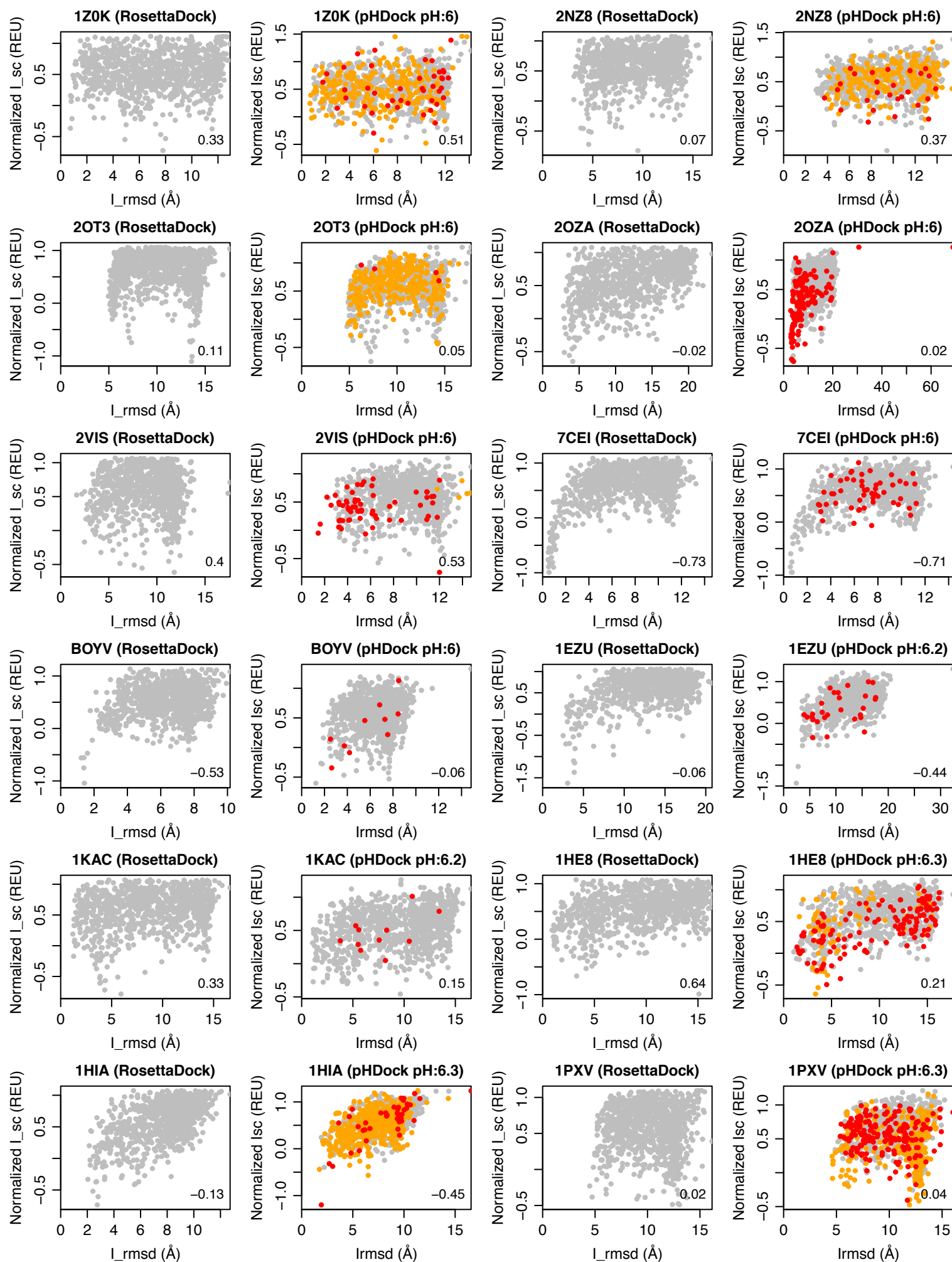

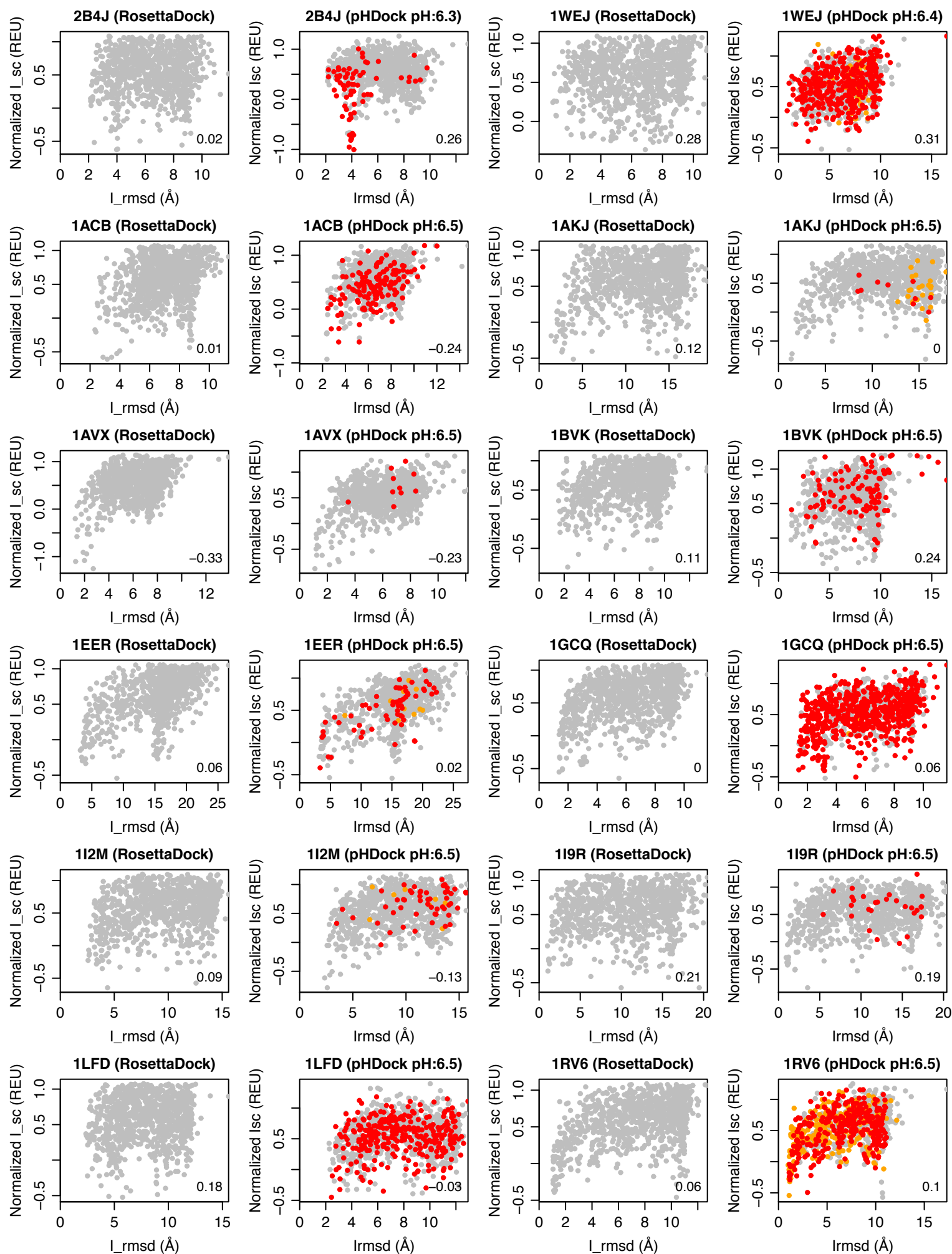

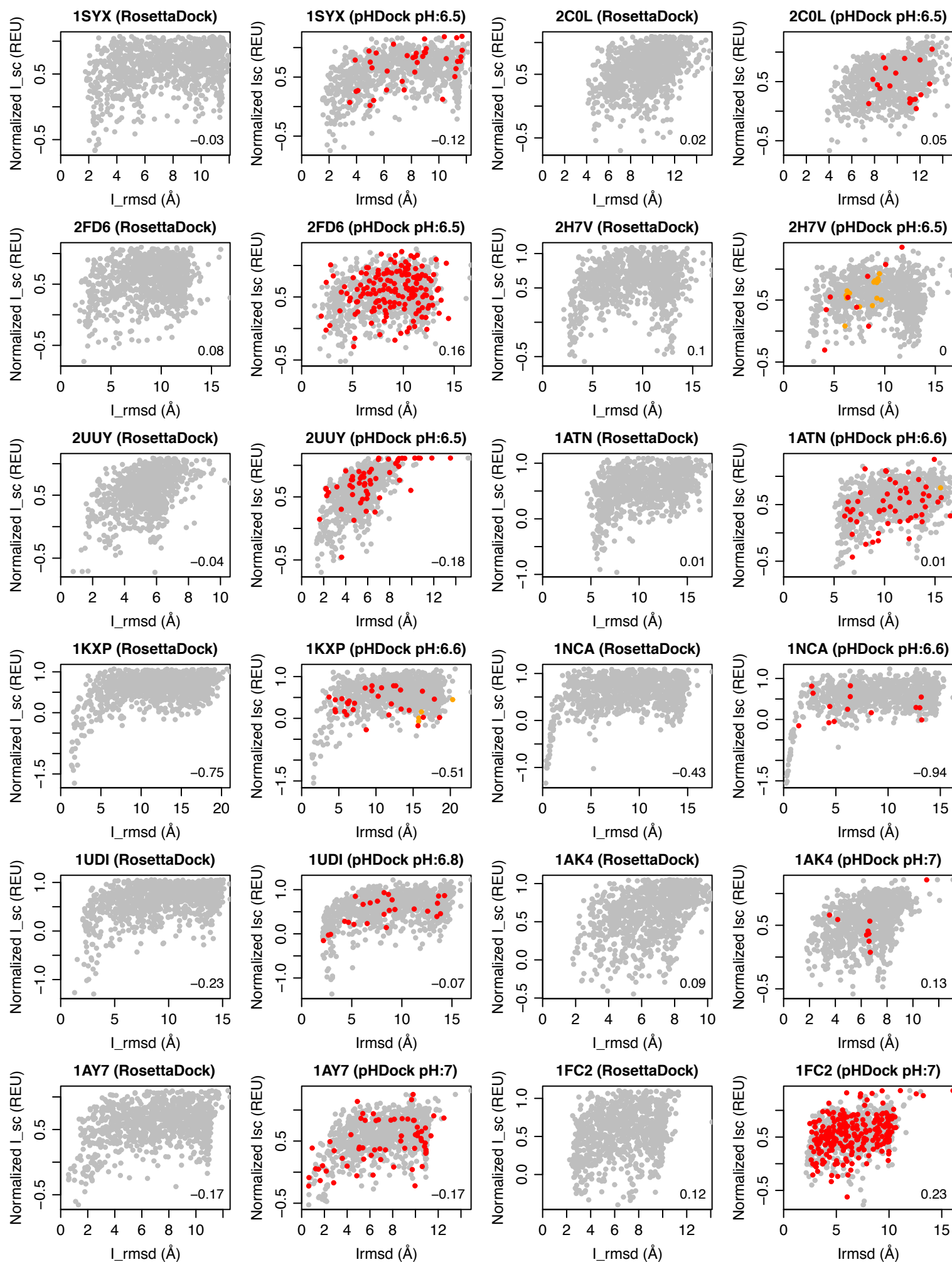

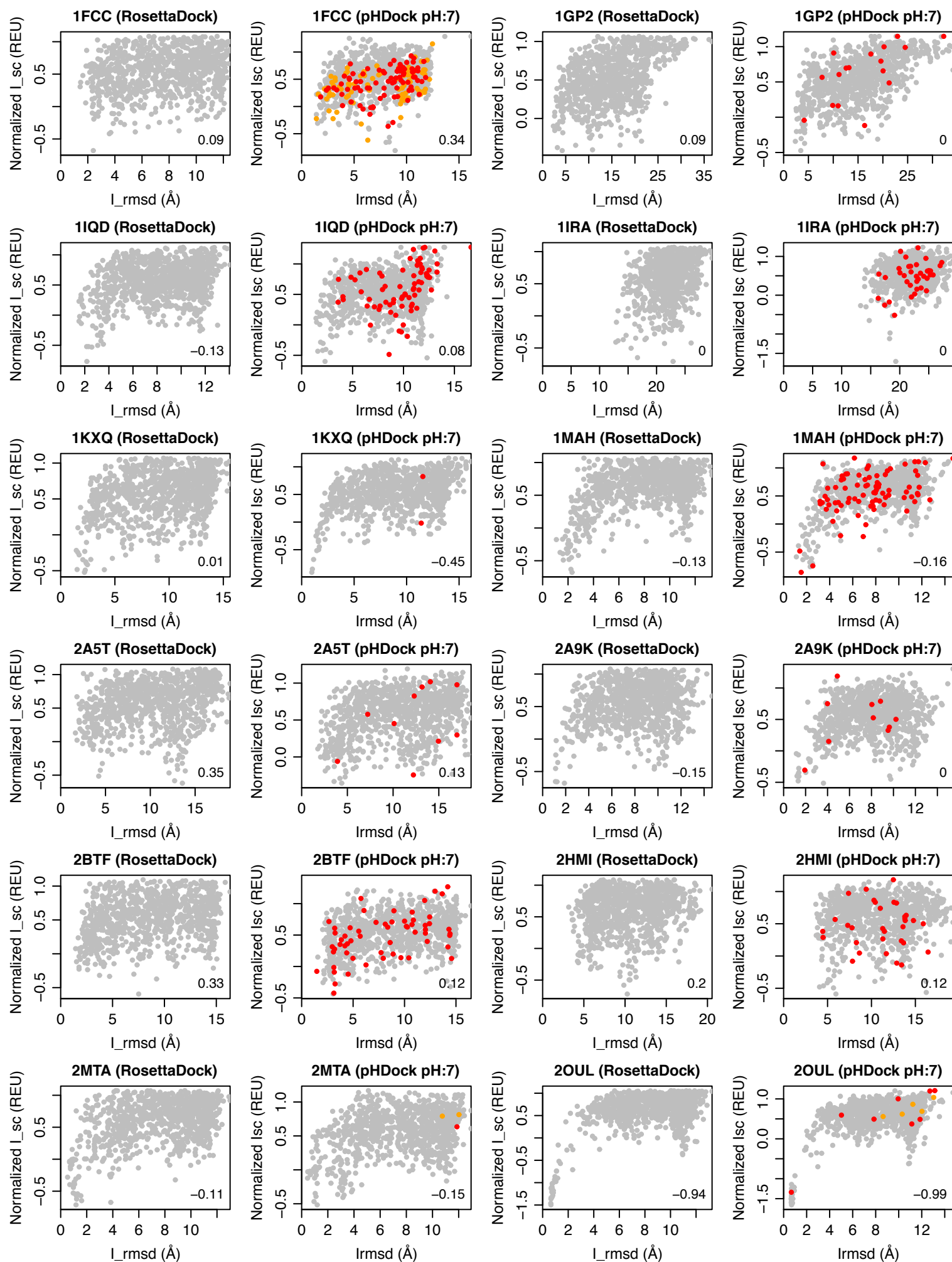

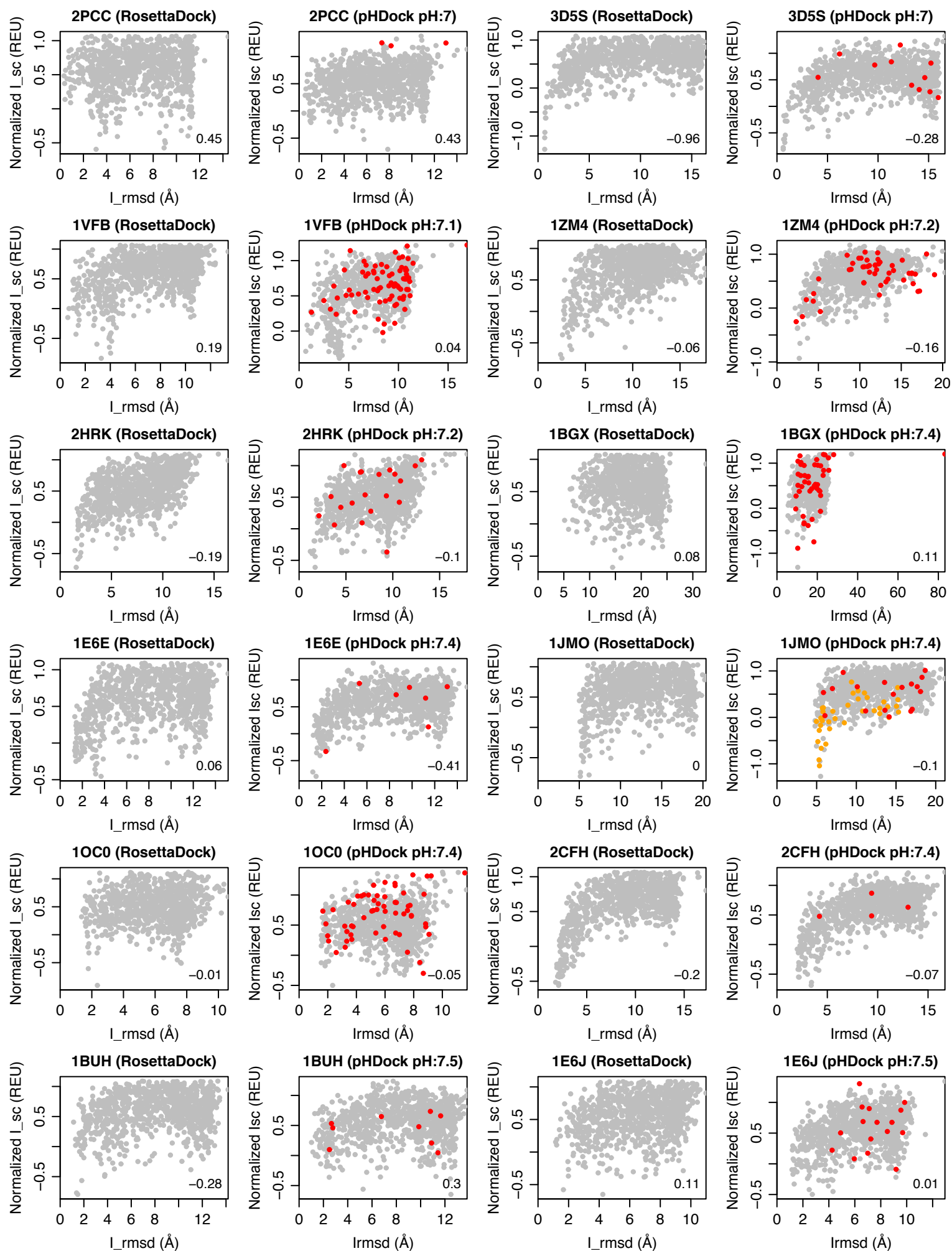

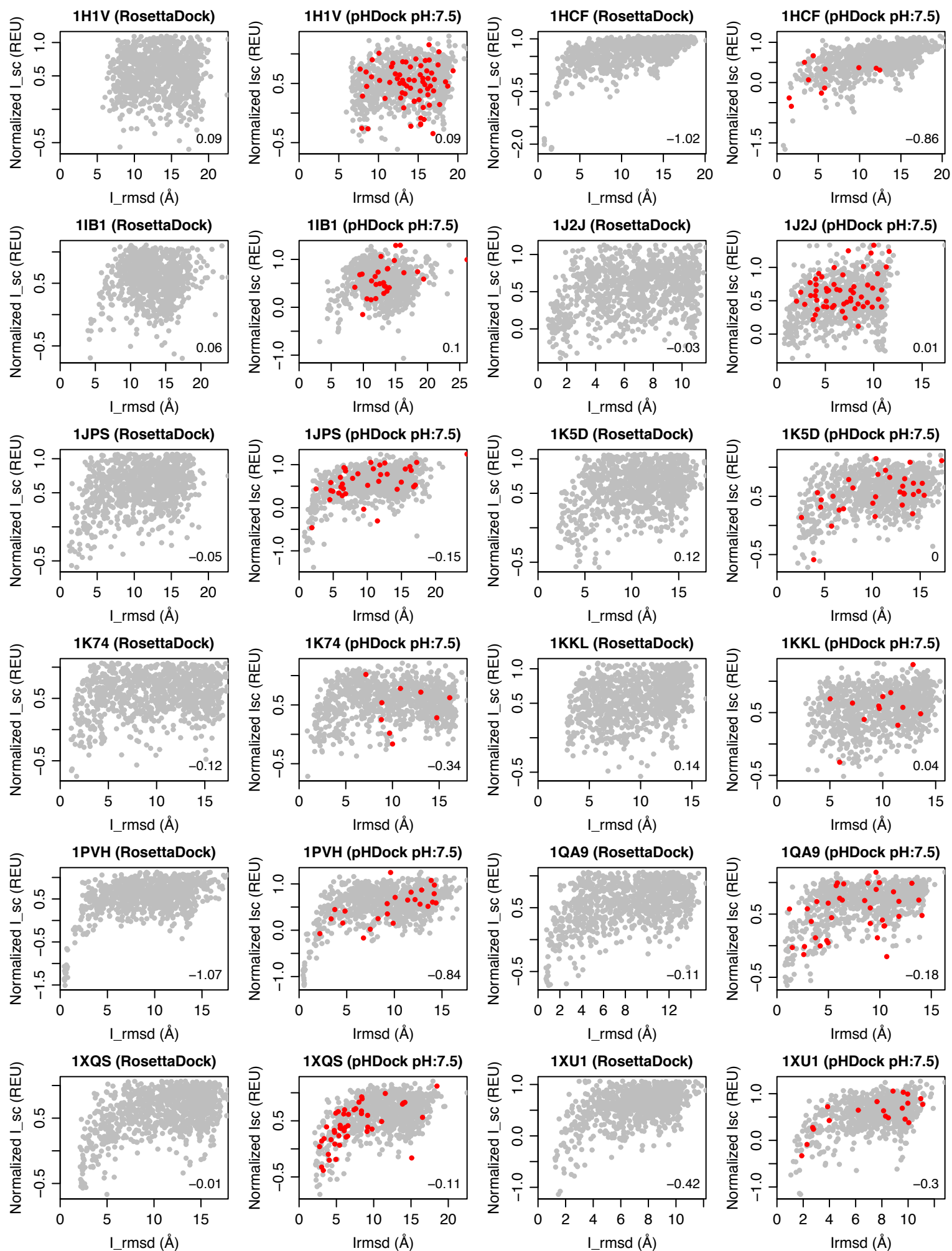

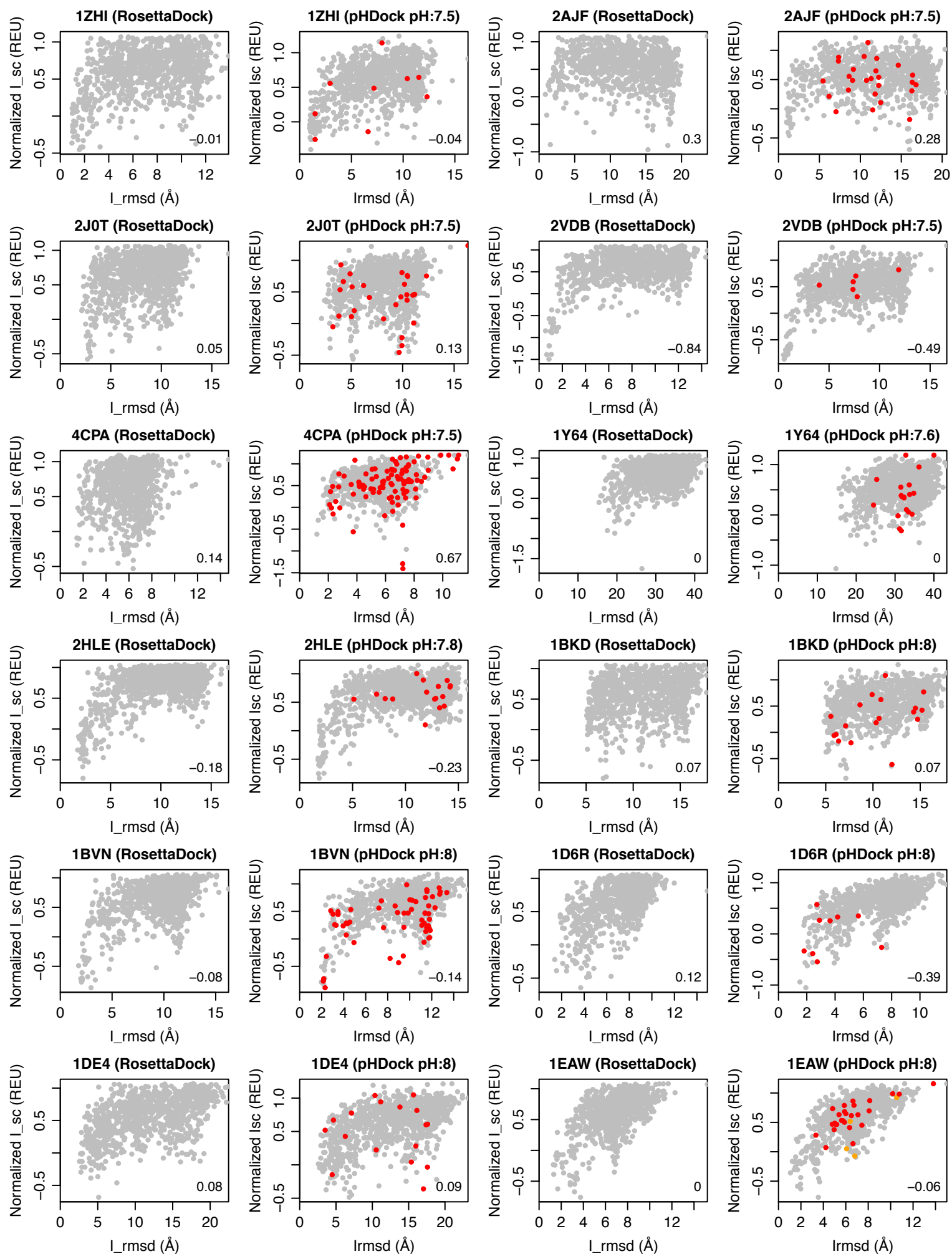

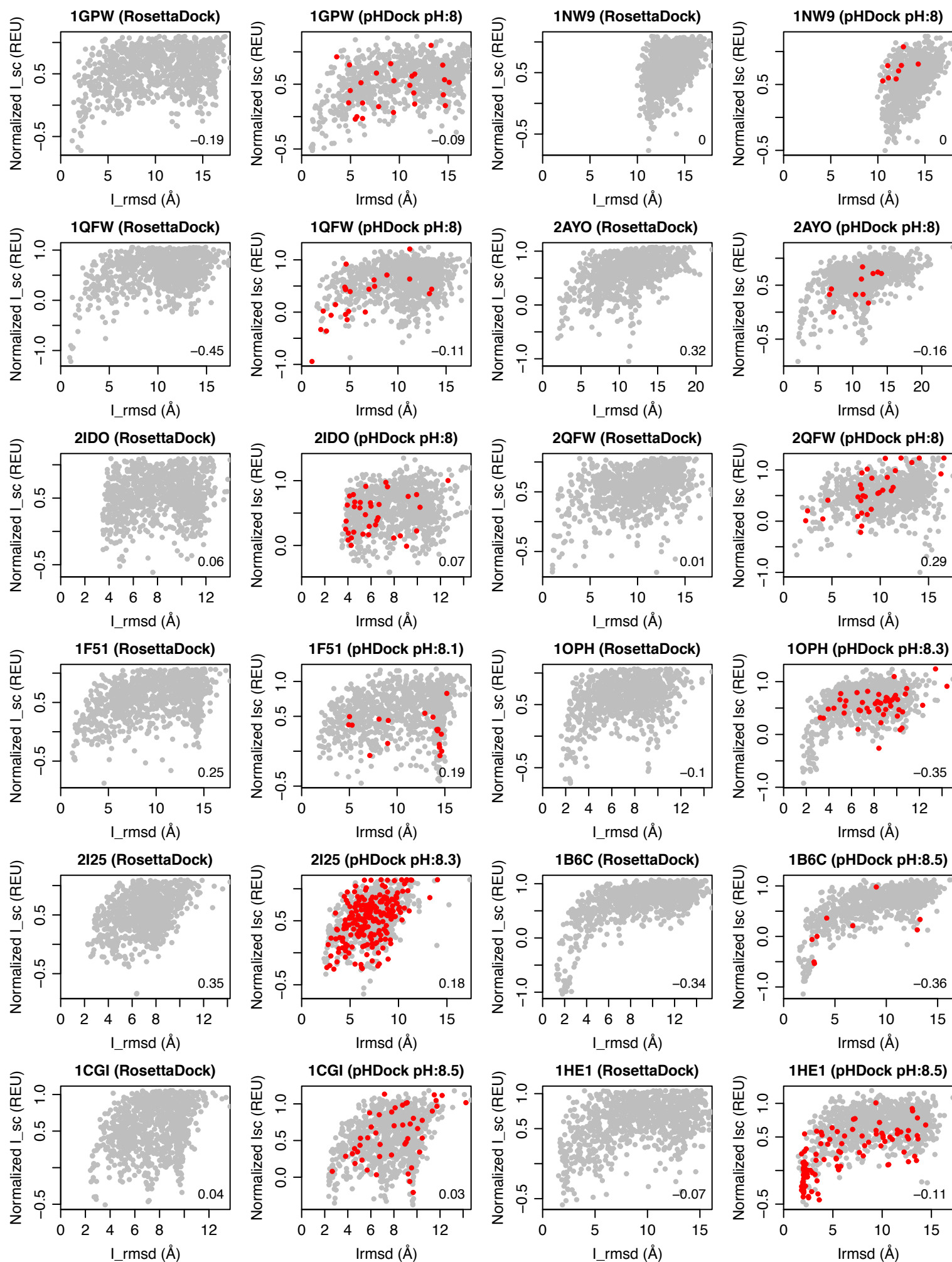

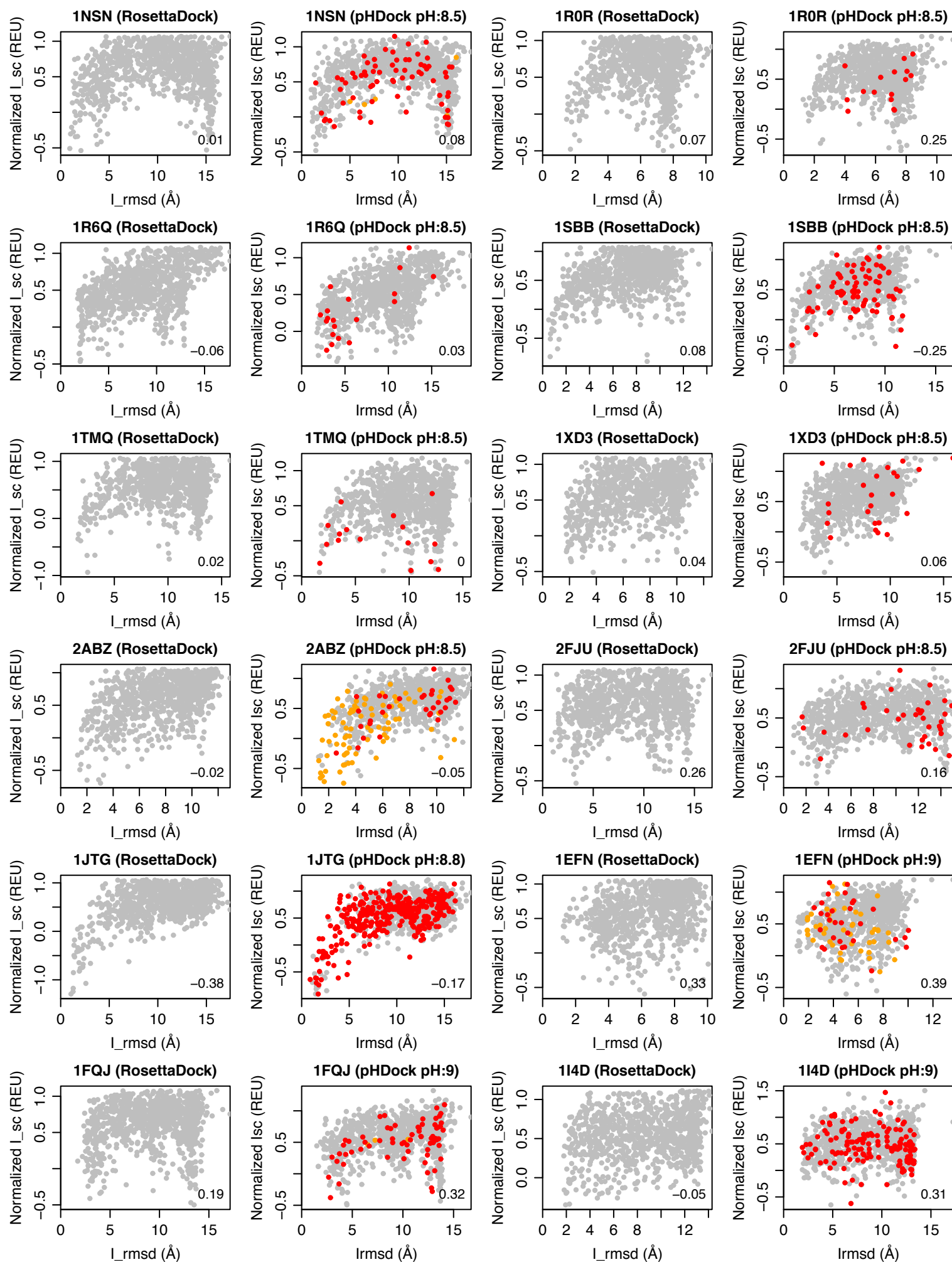

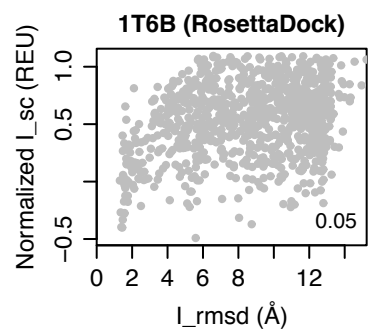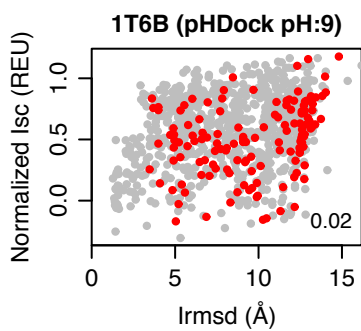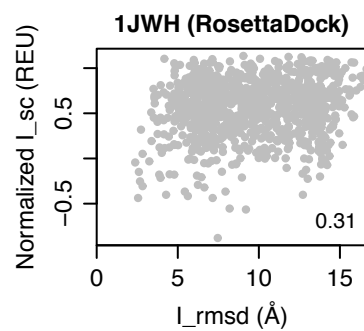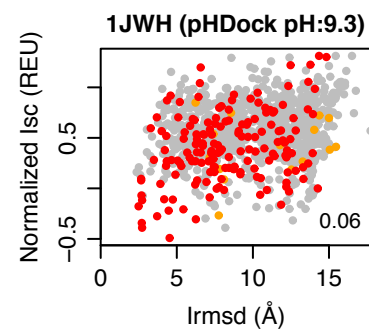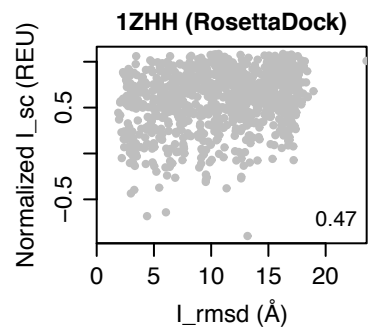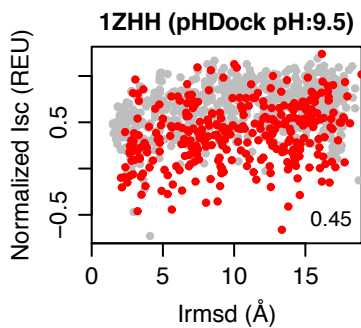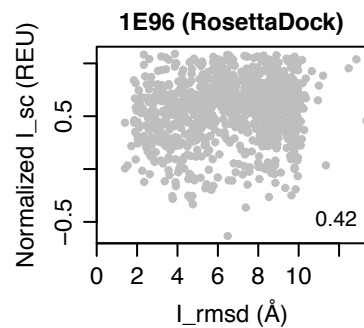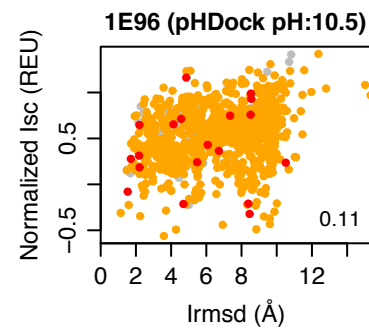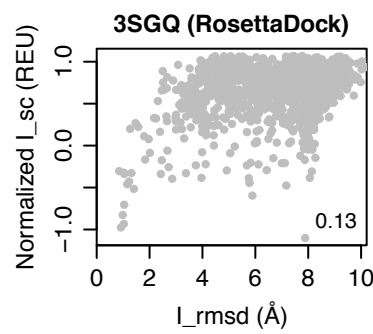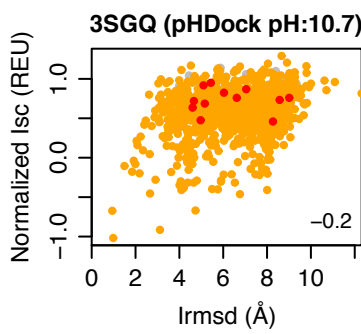

Supplement: S9 Figure — Docking plots for pHDock and RosettaDock highlighting models with nonstandard residue protonation states. Grey, orange and red points represent models containing no nonstandard protonation states, recovered nonstandard protonation states found in the bound complex, and nonstandard protonation states not observed in the bound complex, respectively. Discrimination scores are shown in the bottom right corner of the plots. (PDF) [file pcbi.1004018.s009.pdf]

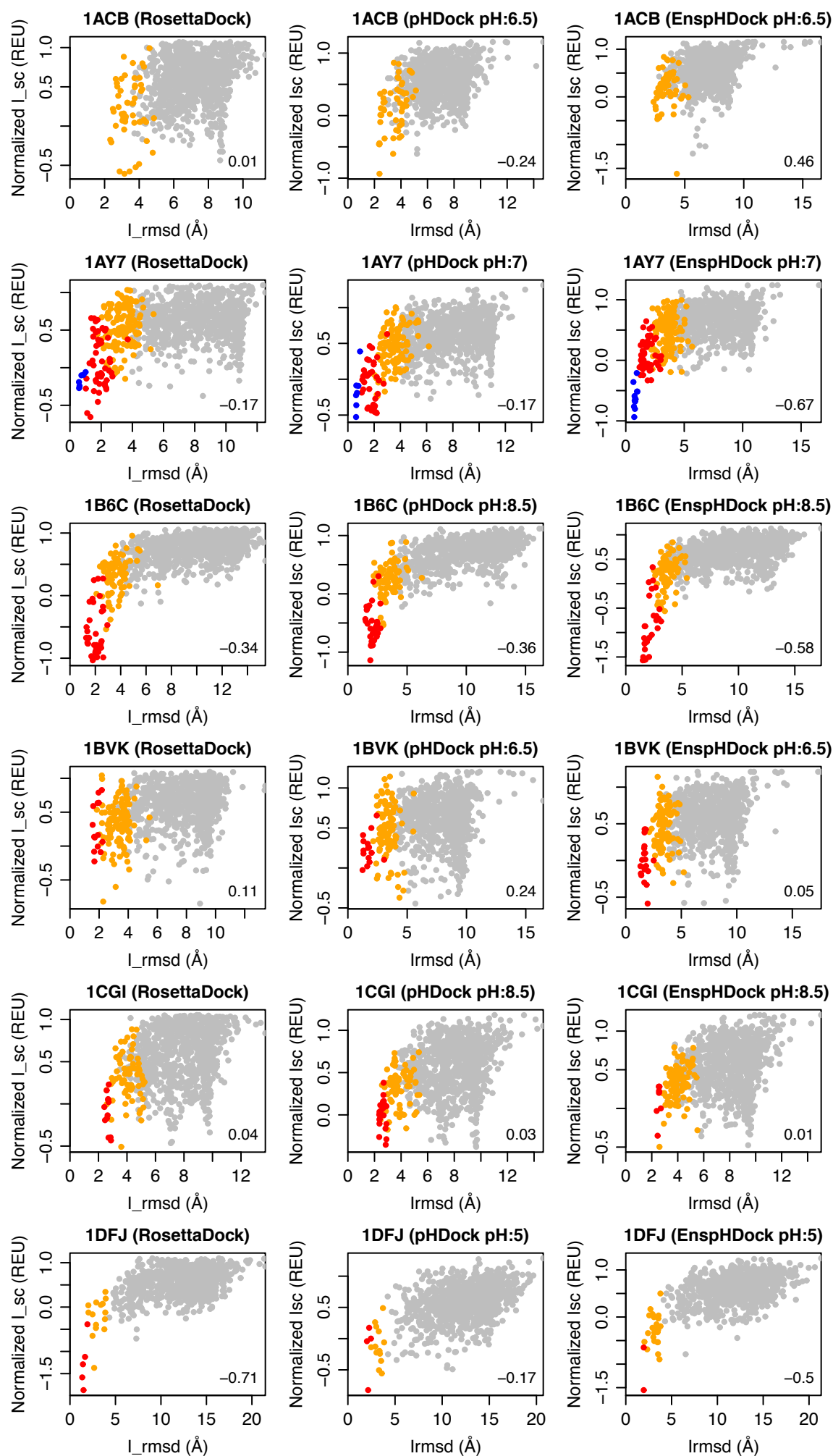

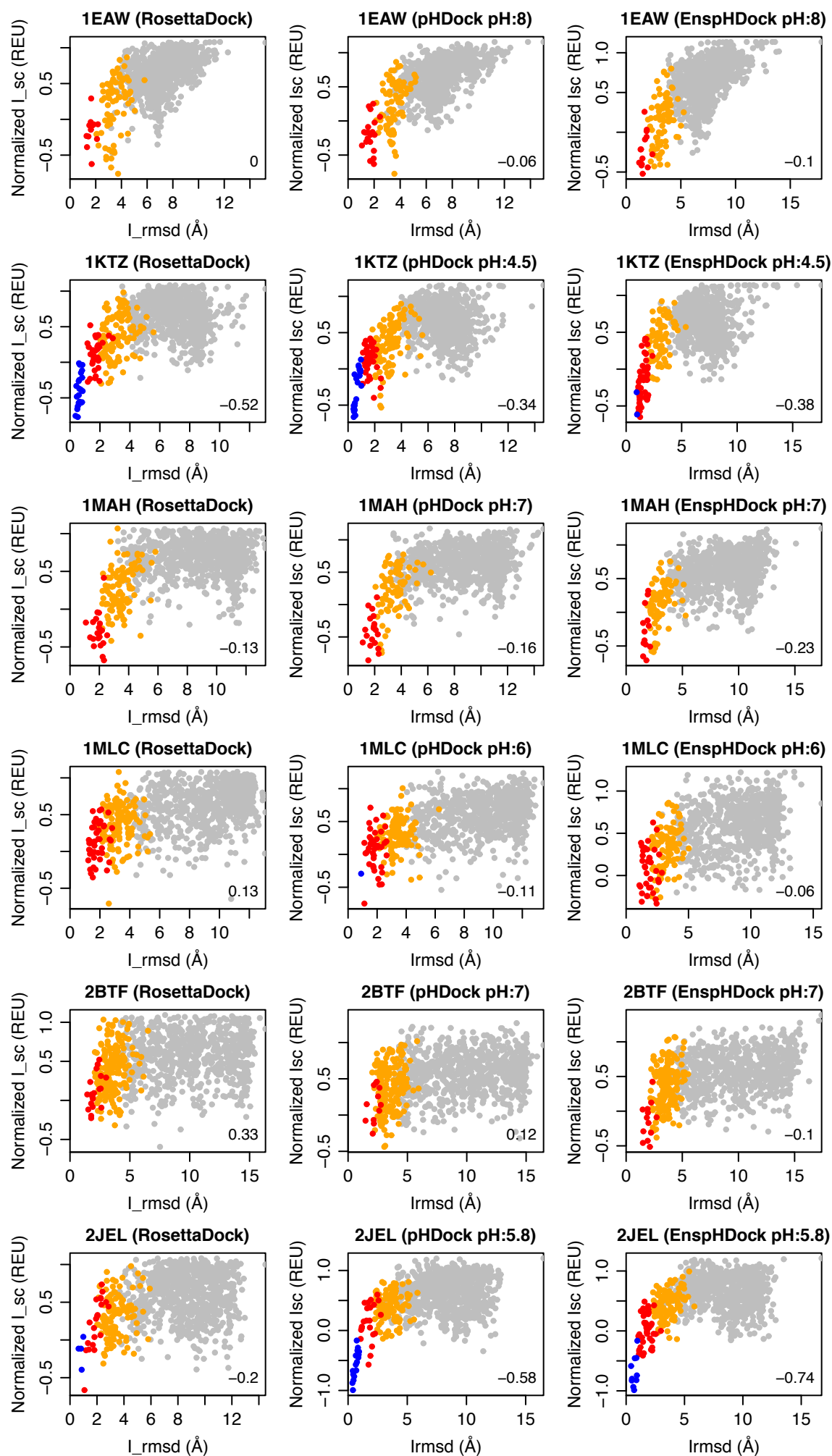

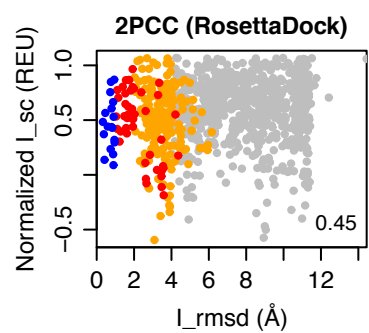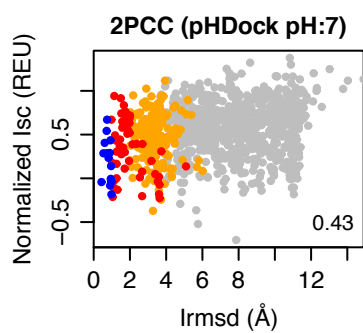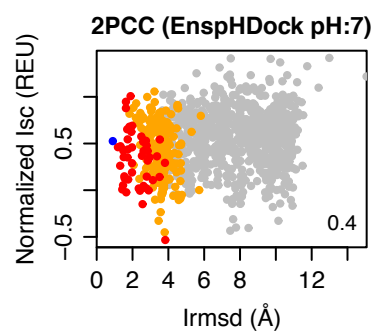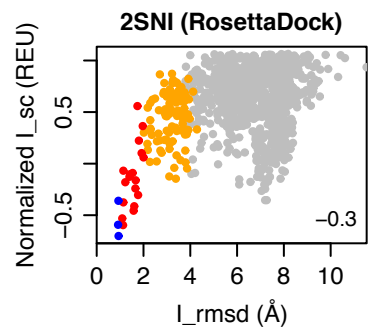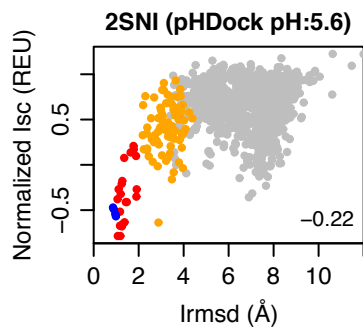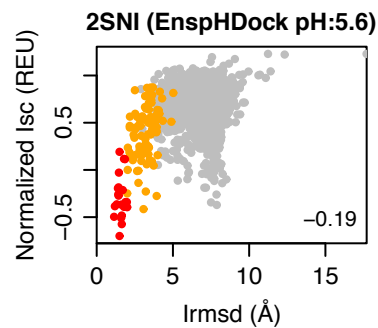

Supplement: S10 Figure — Docking plots comparing ensemble pHDock to pHDock and RosettaDock. Grey, orange, and red points represent incorrect, acceptable-, and medium- quality predictions, respectively. Discrimination scores are shown in the bottom right corner of the plots. (PDF) [file pcbi.1004018.s010.pdf]

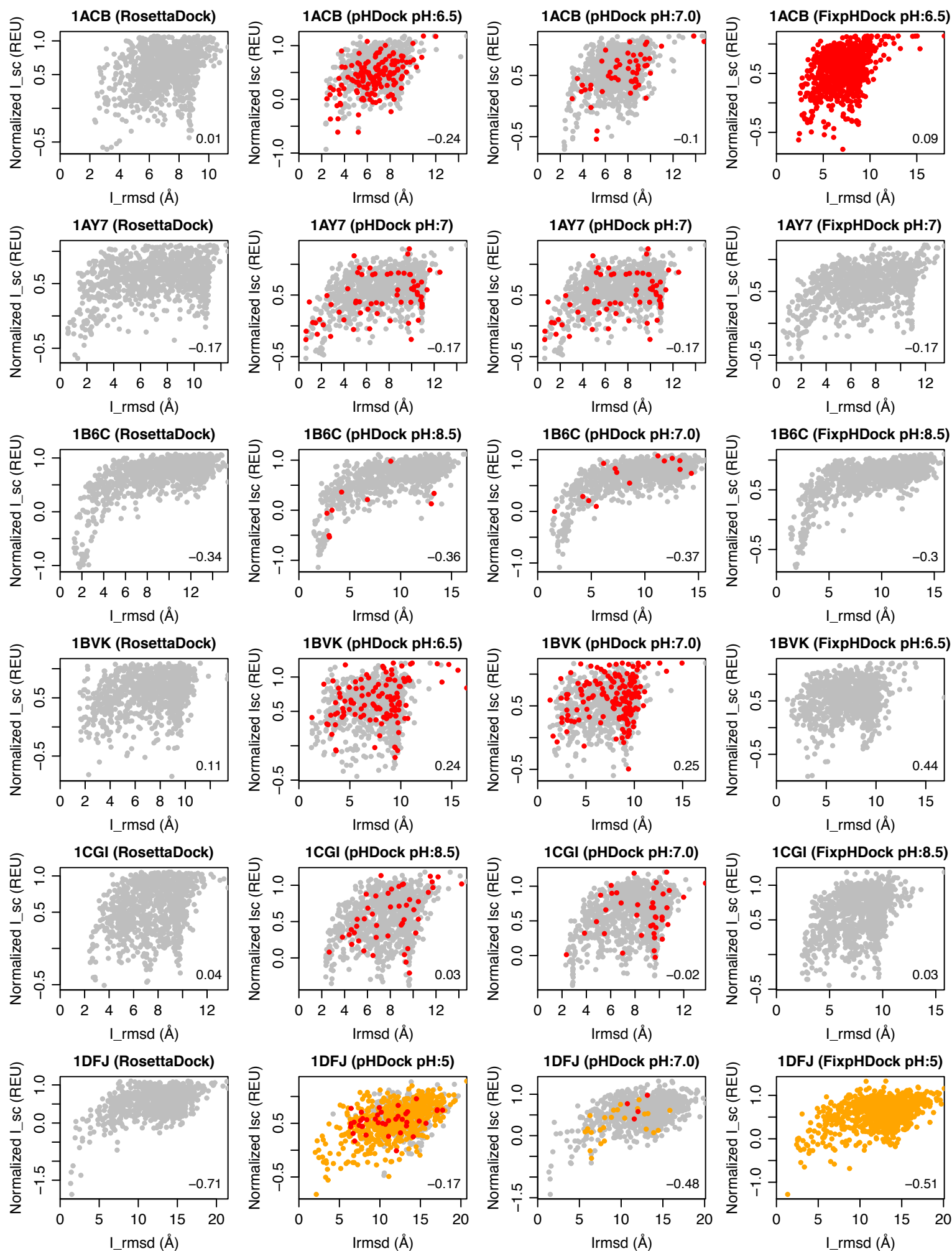

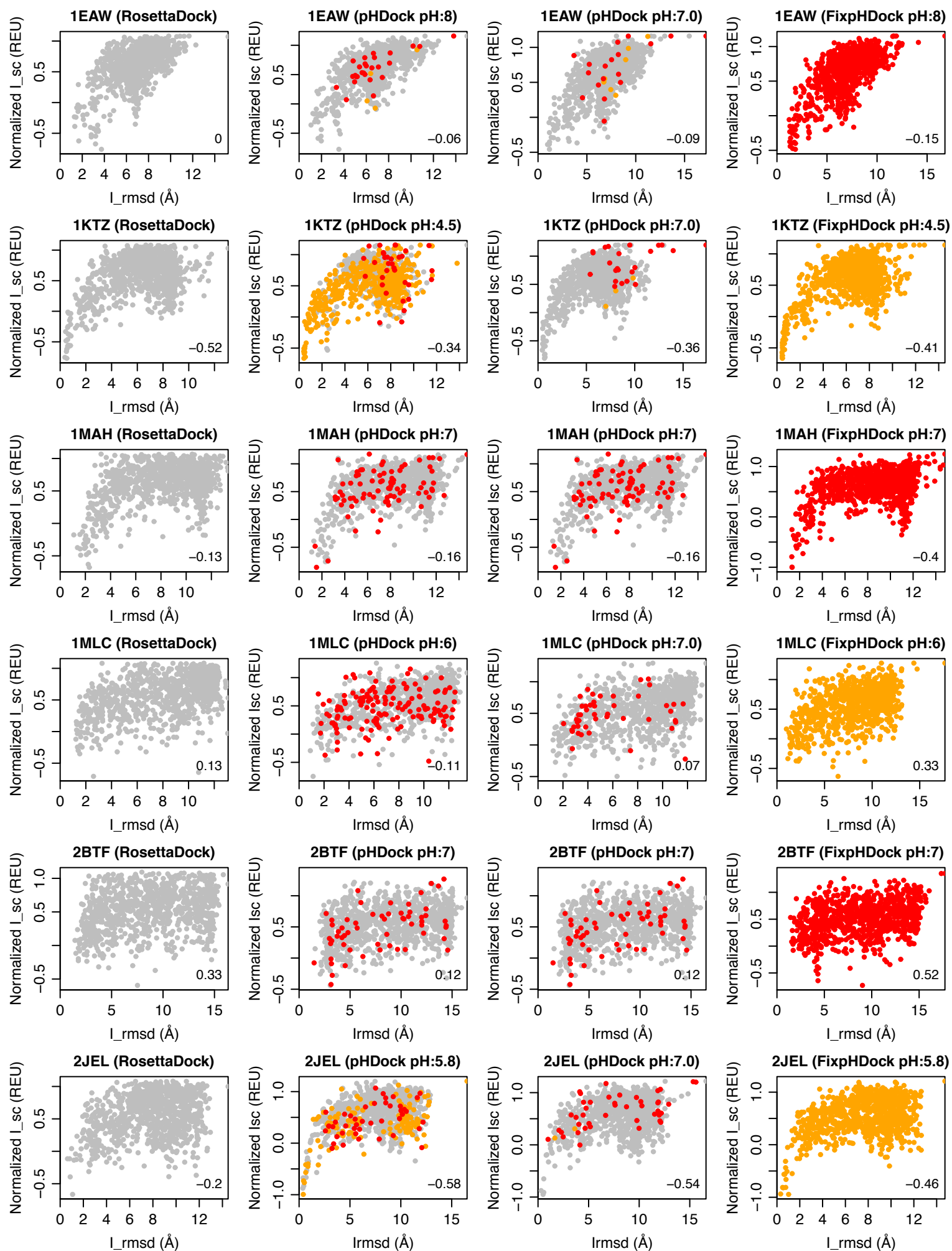

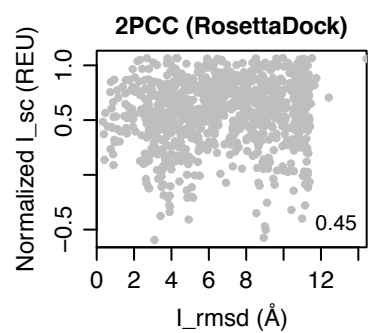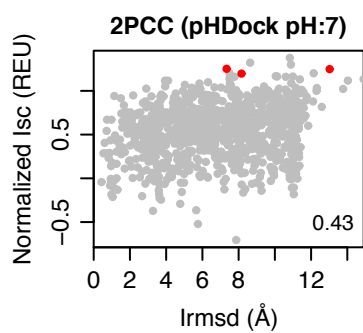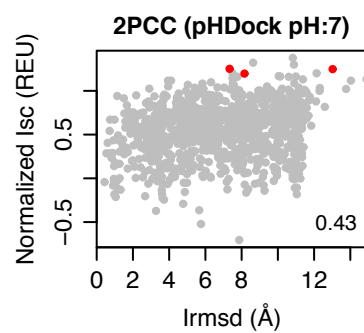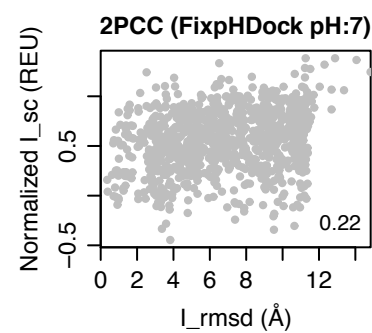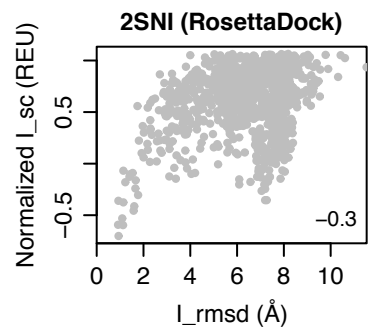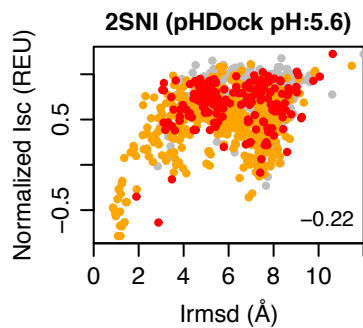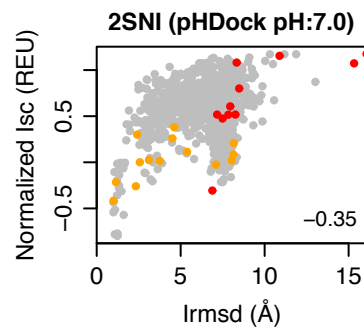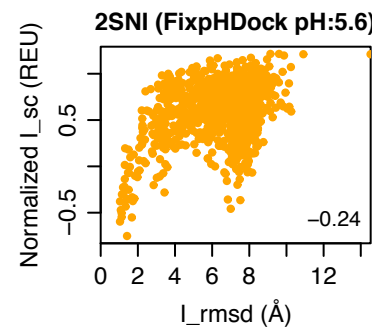

Supplement: S11 Figure — Docking plots comparing RosettaDock, pHDock at crystallization pH, pHDock at pH 7.0 and FixpHDock. Grey, orange and red points represent models containing no nonstandard residue protonation states, recovered nonstandard residue protonation states found in the bound complex, and nonstandard residue protonation states not observed in the bound complex, respectively. In FixpHDock, the protonation states found in the starting unbound complex are held constant during docking. Discrimination scores are shown in the bottom right corner of the plots. (PDF) [file pcbi.1004018.s011.pdf]
