# Supplementary material for: Protein-Protein Docking with Dynamic Residue Protonation States
Source: PLoS Comput Biol. 2014 Dec 11;10(12):e1004018. doi: 10.1371/journal.pcbi.1004018 (PMC4263365; doi:10.1371/journal.pcbi.1004018)
Supplement: S1 Table — Score functions used for the study. Weights for the score terms used during residue side-chain and protonation state sampling, receptor-ligand minimization, and for ranking final docking models. (PDF) [file pcbi.1004018.s012.pdf]

Table S1. Score functions used for the study. Weights for the score terms used during residue side-chain and protonation state sampling, receptor-ligand minimization, and for ranking final docking models.

Score Type*	Packing weights		Docking weights	
	pHDock	RosettaDock	pHDock	RosettaDock
fa_atr	0.80	0.80	0.377	0.338
fa_rep	0.44	0.44	0.005†	0.044‡
fa_sol	0.75	0.75	0.225	0.242
fa_elec	1.00	0.70	0.319	0.026
fa_dun	0.56	0.56	0.080	0.036
hbond_lr_bb	1.17	1.17	0.249	0.245
hbond_sr_bb	1.17	1.17	0.249	0.245
hbond_bb_sc	1.17	1.17	0.249	0.245
hbond_sc	1.10	1.10	0.245	0.245
e_pH	1.00	-	0.210	-
pro_close	1.00	1.00	-	-
rama	0.20	0.20	-	-
omega	0.50	0.50	-	-
p_aa_pp	0.32	0.32	-	-
fa_intra_rep	0.004	0.004	-	-
fa_pair	-	-	-	0.164

* The disulfide score terms (dslf_ss_dst, dslf_cs_ang, dslf_ss_dih, dslf_ca_dst) which have non-zero weights only in RosettaDock docking weights are not listed as they do not aid in the final model discrimination.

†,‡ During the minimization stage, the weights are multiplied by †414 and ‡4.22 respectively.
