# Supplementary material for: Protein-Protein Docking with Dynamic Residue Protonation States
Source: PLoS Comput Biol. 2014 Dec 11;10(12):e1004018. doi: 10.1371/journal.pcbi.1004018 (PMC4263365; doi:10.1371/journal.pcbi.1004018)
Supplement: S2 Table — Docking performance summary. PDB IDs and pH values of the benchmark dataset used for the study. Discrimination scores, N 5 values, Irmsd and f nat of the lowest-Irmsd and top-ranked models generated using pHDock and RosettaDock are also listed. (PDF) [file pcbi.1004018.s013.pdf]

**Table S2. Docking performance summary.** PDB IDs and pH values of the benchmark dataset used for the study. Discrimination scores, $N_5$ values, Irmsd and $f_{nat}$ of the lowest-Irmsd and top-ranked models generated using pHDock and RosettaDock are also listed.

| | | Discrimination Score | | N5 (Mean) | | N5 (SD) | | Best Irmsd | | Best scored Irmsd | | Best scored $f_{nat}$ | |
|---|---|---|---|---|---|---|---|---|---|---|---|---|---|
| **PDB** | **pH** | **pHDock** | **RsDock** | **pHDock** | **RsDock** | **pHDock** | **RsDock** | **pHDock** | **RsDock** | **pHDock** | **RsDock** | **pHDock** | **RsDock** |
| 1A2K | 5.6 | 0.09 | 0.44 | 0.73 | 0.00 | 0.84 | 0.00 | 1.91 | 2.18 | 9.30 | 5.87 | 0.06 | 0.08 |
| 1ACB | 6.5[1] | -0.24 | 0.01 | 3.22 | 4.23 | 1.13 | 1.14 | 2.35 | 2.33 | 2.37 | 3.15 | 0.21 | 0.22 |
| 1AHW | 5.3 | -0.27 | 0.24 | 5.00 | 3.90 | 0.04 | 1.02 | 1.58 | 1.36 | 2.53 | 3.56 | 0.40 | 0.08 |
| 1AK4 | 7 | 0.13 | 0.09 | 0.80 | 2.58 | 0.88 | 1.15 | 1.79 | 1.83 | 5.37 | 5.50 | 0.06 | 0.09 |
| 1AKJ | 6.5 | 0.00 | 0.12 | 2.56 | 2.07 | 1.18 | 1.14 | 1.34 | 1.38 | 15.69 | 3.96 | 0.04 | 0.34 |
| 1ATN | 6.6[2] | 0.01 | 0.01 | 0.00 | 0.00 | 0.00 | 0.00 | 5.08 | 4.64 | 7.00 | 7.68 | 0.05 | 0.07 |
| 1AVX | 6.5 | -0.23 | -0.33 | 4.89 | 5.00 | 0.39 | 0.02 | 1.10 | 1.23 | 1.10 | 2.71 | 0.77 | 0.53 |
| 1AY7 | 7 | -0.17 | -0.17 | 5.00 | 4.25 | 0.01 | 0.86 | 0.63 | 0.58 | 0.63 | 1.32 | 0.74 | 0.74 |
| 1AZS | 5.5 | -0.03 | -0.08 | 1.96 | 1.26 | 1.15 | 1.04 | 1.35 | 1.97 | 1.35 | 2.28 | 0.60 | 0.57 |
| 1B6C | 8.5 | -0.37 | -0.34 | 5.00 | 5.00 | 0.00 | 0.00 | 1.48 | 1.28 | 1.90 | 1.80 | 0.71 | 0.61 |
| 1BGX | 7.4 | 0.11 | 0.08 | 0.00 | 0.00 | 0.00 | 0.00 | 5.18 | 5.15 | 10.31 | 14.44 | 0.08 | 0.00 |
| 1BJ1 | 6 | -0.17 | -0.28 | 4.94 | 4.98 | 0.28 | 0.17 | 1.25 | 1.11 | 1.50 | 1.11 | 0.56 | 0.71 |
| 1BKD | 8 | 0.07 | 0.07 | 0.00 | 0.00 | 0.00 | 0.00 | 4.83 | 5.06 | 7.12 | 6.82 | 0.11 | 0.12 |
| 1BUH | 7.5 | 0.30 | -0.28 | 0.88 | 4.27 | 0.92 | 1.09 | 1.02 | 1.13 | 12.49 | 1.49 | 0.07 | 0.58 |
| 1BVK | 6.5 | 0.24 | 0.11 | 0.00 | 1.95 | 0.00 | 1.23 | 1.25 | 1.56 | 5.70 | 8.92 | 0.18 | 0.06 |
| 1BVN | 8 | -0.14 | -0.08 | 4.79 | 4.96 | 0.53 | 0.27 | 1.96 | 1.77 | 2.31 | 2.97 | 0.42 | 0.30 |
| 1CGI | 8.5[3] | 0.03 | 0.04 | 1.06 | 2.62 | 0.95 | 1.20 | 2.36 | 2.41 | 9.65 | 3.59 | 0.06 | 0.24 |
| 1CLV | 5.4 | -0.14 | -0.34 | 4.96 | 4.85 | 0.22 | 0.47 | 1.66 | 1.67 | 1.96 | 1.86 | 0.46 | 0.62 |
| 1D6R | 8 | -0.39 | 0.13 | 4.81 | 1.71 | 0.54 | 1.15 | 1.51 | 1.38 | 1.93 | 3.69 | 0.53 | 0.12 |
| 1DE4 | 8 | 0.09 | 0.08 | 0.80 | 0.63 | 0.87 | 0.83 | 2.57 | 2.57 | 6.82 | 5.13 | 0.24 | 0.31 |
| 1DFJ | 5[4] | -0.17 | -0.71 | 3.19 | 4.55 | 1.33 | 0.76 | 1.64 | 1.35 | 2.14 | 1.48 | 0.40 | 0.62 |
| 1DQJ | 4.6 | 0.03 | 0.14 | 1.99 | 1.00 | 1.25 | 0.93 | 2.16 | 2.63 | 2.73 | 12.40 | 0.53 | 0.00 |
| 1E4K | 5.8 | 0.01 | -0.04 | 0.19 | 1.56 | 0.50 | 1.09 | 3.21 | 3.66 | 4.69 | 3.66 | 0.21 | 0.18 |
| 1E6E | 7.4 | -0.42 | 0.06 | 4.78 | 4.65 | 0.53 | 0.65 | 1.21 | 1.29 | 1.50 | 3.69 | 0.71 | 0.40 |
| 1E6J | 7.5 | 0.01 | 0.11 | 3.87 | 1.42 | 1.00 | 1.03 | 1.15 | 1.15 | 2.72 | 4.24 | 0.51 | 0.31 |
| 1E96 | 10.5 | 0.11 | 0.42 | 1.20 | 0.91 | 1.03 | 0.93 | 1.13 | 1.39 | 3.59 | 6.49 | 0.58 | 0.06 |

| | | Discrimination Score | | N5 (Mean) | | N5 (SD) | | Best Irmsd | | Best scored Irmsd | | Best scored $f_{nat}$ | |
|---|---|---|---|---|---|---|---|---|---|---|---|---|---|
| **PDB** | **pH** | **pHDock** | **RsDock** | **pHDock** | **RsDock** | **pHDock** | **RsDock** | **pHDock** | **RsDock** | **pHDock** | **RsDock** | **pHDock** | **RsDock** |
| 1EAW | 8 | -0.06 | 0.00 | 5.00 | 5.00 | 0.00 | 0.04 | 1.06 | 1.28 | 3.55 | 3.73 | 0.15 | 0.15 |
| 1EER | 6.5 | 0.02 | 0.06 | 1.02 | 1.61 | 0.94 | 1.10 | 3.16 | 3.17 | 15.03 | 8.96 | 0.00 | 0.00 |
| 1EFN | 9 | 0.39 | 0.33 | 0.83 | 0.38 | 0.87 | 0.67 | 1.08 | 1.25 | 5.02 | 6.18 | 0.22 | 0.13 |
| 1EZU | 6.2 | -0.44 | -0.06 | 3.33 | 4.15 | 1.30 | 1.01 | 2.42 | 2.51 | 2.42 | 3.06 | 0.36 | 0.29 |
| 1F34 | 3.5 | 0.19 | 0.24 | 0.96 | 2.89 | 0.95 | 1.20 | 2.56 | 1.92 | 8.26 | 8.25 | 0.04 | 0.05 |
| 1F51 | 8.1 | 0.19 | 0.25 | 0.33 | 0.90 | 0.62 | 1.08 | 1.77 | 1.22 | 6.23 | 4.54 | 0.04 | 0.19 |
| 1F6M | 6 | 0.07 | 0.08 | 0.00 | 0.00 | 0.00 | 0.00 | 5.56 | 5.58 | 11.67 | 7.47 | 0.00 | 0.06 |
| 1FC2 | 7* | 0.23 | 0.12 | 0.58 | 1.27 | 0.79 | 1.04 | 2.13 | 2.24 | 7.55 | 6.26 | 0.13 | 0.25 |
| 1FCC | 7[5] | 0.34 | 0.09 | 0.36 | 3.10 | 0.66 | 1.39 | 1.41 | 1.43 | 8.91 | 2.41 | 0.05 | 0.37 |
| 1FFW | 5.5 | 0.15 | 0.35 | 3.01 | 1.09 | 1.15 | 0.96 | 1.57 | 1.77 | 3.49 | 8.30 | 0.55 | 0.14 |
| 1FLE | 5.9 | -0.05 | 0.07 | 3.52 | 3.65 | 1.09 | 1.11 | 2.13 | 2.18 | 3.38 | 3.39 | 0.25 | 0.27 |
| 1FQJ | 9 | 0.32 | 0.19 | 1.27 | 1.66 | 1.08 | 1.14 | 1.50 | 1.72 | 4.46 | 13.50 | 0.18 | 0.02 |
| 1FSK | 4 | -0.68 | -0.55 | 5.00 | 5.00 | 0.00 | 0.00 | 0.74 | 0.78 | 1.42 | 0.85 | 0.74 | 0.82 |
| 1GCQ | 6.5 | 0.06 | 0.00 | 3.39 | 3.96 | 1.06 | 1.01 | 1.37 | 1.38 | 3.22 | 5.51 | 0.33 | 0.13 |
| 1GHQ | 6 | 0.30 | 0.08 | 0.02 | 1.11 | 0.17 | 0.96 | 0.51 | 0.48 | 4.34 | 9.50 | 0.27 | 0.14 |
| 1GL1 | 5 | 0.05 | 0.09 | 1.90 | 2.30 | 1.14 | 1.14 | 2.09 | 2.00 | 4.54 | 5.48 | 0.20 | 0.03 |
| 1GLA | 6[6] | 0.25 | 0.41 | 1.04 | 0.00 | 0.94 | 0.00 | 1.72 | 1.99 | 8.85 | 5.53 | 0.13 | 0.21 |
| 1GP2 | 7 | 0.00 | 0.09 | 2.52 | 0.92 | 1.21 | 0.92 | 2.38 | 2.32 | 3.46 | 4.23 | 0.34 | 0.22 |
| 1GPW | 8 | -0.09 | -0.19 | 4.98 | 4.78 | 0.16 | 0.61 | 0.96 | 1.21 | 1.10 | 2.15 | 0.60 | 0.38 |
| 1H1V | 7.5 | 0.09 | 0.09 | 0.00 | 0.00 | 0.00 | 0.00 | 5.96 | 5.76 | 12.39 | 17.33 | 0.00 | 0.00 |
| 1HCF | 7.5 | -0.86 | -1.03 | 3.19 | 4.94 | 1.35 | 0.29 | 0.79 | 0.75 | 0.97 | 1.58 | 0.77 | 0.74 |
| 1HE1 | 8.5 | -0.11 | -0.07 | 5.00 | 3.64 | 0.00 | 1.14 | 1.50 | 1.52 | 2.29 | 1.89 | 0.50 | 0.38 |
| 1HE8 | 6.3 | 0.21 | 0.64 | 2.25 | 1.41 | 1.31 | 1.08 | 1.06 | 0.90 | 3.33 | 15.09 | 0.73 | 0.00 |
| 1HIA | 6.3 | -0.45 | -0.13 | 3.15 | 3.54 | 1.10 | 1.19 | 1.73 | 2.04 | 1.92 | 2.75 | 0.36 | 0.38 |
| 1I2M | 6.5 | -0.14 | 0.09 | 4.37 | 0.76 | 0.97 | 0.93 | 2.35 | 2.58 | 2.60 | 4.38 | 0.46 | 0.19 |
| 1I4D | 9 | 0.31 | -0.05 | 0.00 | 2.64 | 0.00 | 1.22 | 1.91 | 1.79 | 4.81 | 1.94 | 0.16 | 0.49 |
| 1I9R | 6.5 | 0.19 | 0.21 | 3.07 | 0.11 | 1.14 | 0.39 | 0.93 | 1.01 | 3.52 | 19.47 | 0.24 | 0.00 |
| 1IB1 | 7.5 | 0.10 | 0.06 | 1.03 | 0.01 | 0.98 | 0.09 | 3.21 | 3.96 | 16.24 | 17.35 | 0.00 | 0.00 |

| | | Discrimination Score | | N5 (Mean) | | N5 (SD) | | Best Irmsd | | Best scored Irmsd | | Best scored $f_{nat}$ | |
|---|---|---|---|---|---|---|---|---|---|---|---|---|---|
| **PDB** | **pH** | **pHDock** | **RsDock** | **pHDock** | **RsDock** | **pHDock** | **RsDock** | **pHDock** | **RsDock** | **pHDock** | **RsDock** | **pHDock** | **RsDock** |
| 1IBR | 6 | 0.04 | 0.12 | 0.00 | 0.00 | 0.00 | 0.00 | 5.13 | 5.89 | 7.66 | 16.61 | 0.04 | 0.00 |
| 1IJK | 5.6 | 0.29 | 0.12 | 0.20 | 0.25 | 0.51 | 0.58 | 1.57 | 1.74 | 9.50 | 6.96 | 0.04 | 0.15 |
| 1IQD | 7 | 0.08 | -0.13 | 2.88 | 4.99 | 1.19 | 0.14 | 1.41 | 1.53 | 2.29 | 2.17 | 0.34 | 0.43 |
| 1IRA | 7 | 0.00 | 0.00 | 0.00 | 0.00 | 0.00 | 0.00 | 13.27 | 12.85 | 19.31 | 22.89 | 0.00 | 0.00 |
| 1J2J | 7.5[7] | 0.01 | -0.03 | 2.21 | 4.61 | 1.31 | 0.70 | 0.78 | 0.71 | 1.60 | 1.56 | 0.52 | 0.60 |
| 1JIW | 5.6 | 0.01 | 0.48 | 1.92 | 0.00 | 1.24 | 0.00 | 0.75 | 0.75 | 13.16 | 11.26 | 0.09 | 0.13 |
| 1JK9 | 6 | -0.36 | -0.15 | 4.99 | 3.90 | 0.12 | 0.98 | 2.24 | 2.11 | 2.55 | 2.55 | 0.72 | 0.68 |
| 1JMO | 7.4 | -0.10 | 0.00 | 0.00 | 0.00 | 0.00 | 0.00 | 4.79 | 5.01 | 5.50 | 5.13 | 0.31 | 0.34 |
| 1JPS | 7.5 | -0.15 | -0.05 | 4.98 | 3.52 | 0.14 | 1.06 | 0.79 | 1.24 | 2.12 | 2.24 | 0.54 | 0.33 |
| 1JTG | 8.8 | -0.17 | -0.38 | 5.00 | 5.00 | 0.01 | 0.07 | 0.90 | 1.06 | 1.74 | 1.06 | 0.49 | 0.63 |
| 1JWH | 9.3 | 0.06 | 0.31 | 1.73 | 0.54 | 1.11 | 0.75 | 2.36 | 2.38 | 4.52 | 7.46 | 0.14 | 0.14 |
| 1JZD | 4.9 | 0.13 | 0.04 | 0.00 | 0.33 | 0.00 | 0.62 | 3.42 | 3.06 | 18.57 | 5.40 | 0.00 | 0.11 |
| 1K4C | 5.4 | -0.38 | -0.32 | 5.00 | 5.00 | 0.01 | 0.00 | 1.12 | 1.32 | 1.37 | 1.60 | 0.67 | 0.67 |
| 1K5D | 7.5 | 0.00 | 0.12 | 4.00 | 0.01 | 0.93 | 0.14 | 1.90 | 2.14 | 3.23 | 6.49 | 0.25 | 0.01 |
| 1K74 | 7.5 | -0.35 | -0.12 | 2.71 | 3.19 | 1.19 | 1.34 | 0.85 | 1.15 | 0.93 | 1.69 | 0.76 | 0.64 |
| 1KAC | 6.2 | 0.15 | 0.33 | 1.29 | 2.13 | 1.13 | 1.21 | 1.06 | 1.20 | 9.64 | 5.92 | 0.07 | 0.20 |
| 1KKL | 7.5 | 0.04 | 0.14 | 1.67 | 0.21 | 1.16 | 0.52 | 2.74 | 2.84 | 6.29 | 10.00 | 0.10 | 0.10 |
| 1KLU | 5.2 | -0.31 | -0.27 | 4.41 | 3.31 | 0.82 | 1.27 | 0.65 | 0.58 | 0.86 | 0.58 | 0.69 | 0.84 |
| 1KTZ | 4.5[8] | -0.35 | -0.52 | 5.00 | 5.00 | 0.00 | 0.00 | 0.41 | 0.37 | 0.42 | 0.51 | 0.93 | 0.93 |
| 1KXP | 6.6 | -0.51 | -0.75 | 4.87 | 5.00 | 0.41 | 0.00 | 1.38 | 1.35 | 1.61 | 1.71 | 0.55 | 0.52 |
| 1KXQ | 7 | -0.46 | 0.01 | 4.63 | 3.93 | 0.76 | 0.96 | 0.96 | 1.44 | 0.96 | 2.20 | 0.66 | 0.51 |
| 1LFD | 6.5 | -0.03 | 0.19 | 1.89 | 0.22 | 1.15 | 0.55 | 2.26 | 2.37 | 2.44 | 5.76 | 0.52 | 0.09 |
| 1M10 | 5.5 | 0.13 | 0.15 | 0.00 | 0.00 | 0.04 | 0.00 | 3.76 | 3.60 | 6.28 | 8.49 | 0.08 | 0.00 |
| 1MAH | 7[9] | -0.16 | -0.13 | 5.00 | 4.99 | 0.04 | 0.13 | 1.10 | 1.09 | 1.52 | 2.32 | 0.52 | 0.38 |
| 1ML0 | 4.1 | -0.56 | -0.86 | 5.00 | 5.00 | 0.05 | 0.00 | 1.07 | 1.03 | 1.16 | 1.20 | 0.80 | 0.77 |
| 1MLC | 6[10] | -0.11 | 0.13 | 2.49 | 2.60 | 1.17 | 1.17 | 0.90 | 1.22 | 1.12 | 2.59 | 0.71 | 0.37 |
| 1MQ8 | 4.6 | -0.08 | -0.22 | 4.88 | 5.00 | 0.40 | 0.07 | 1.43 | 1.50 | 2.29 | 2.27 | 0.52 | 0.49 |
| 1N8O | 4 | -1.12 | -1.07 | 5.00 | 3.32 | 0.00 | 1.11 | 1.05 | 1.20 | 1.05 | 1.20 | 0.78 | 0.71 |

| | | Discrimination Score | | N5 (Mean) | | N5 (SD) | | Best Irmsd | | Best scored Irmsd | | Best scored $f_{nat}$ | |
|---|---|---|---|---|---|---|---|---|---|---|---|---|---|
| **PDB** | **pH** | **pHDock** | **RsDock** | **pHDock** | **RsDock** | **pHDock** | **RsDock** | **pHDock** | **RsDock** | **pHDock** | **RsDock** | **pHDock** | **RsDock** |
| 1NCA | 6.6[11] | -0.94 | -0.43 | 5.00 | 4.00 | 0.00 | 0.97 | 0.26 | 0.32 | 0.26 | 0.32 | 0.88 | 0.85 |
| 1NSN | 8.5[12] | 0.08 | 0.01 | 2.88 | 3.08 | 1.20 | 1.13 | 0.99 | 0.93 | 1.47 | 2.09 | 0.47 | 0.50 |
| 1NW9 | 8 | 0.00 | 0.00 | 0.00 | 0.00 | 0.00 | 0.00 | 9.96 | 9.99 | 10.77 | 11.18 | 0.10 | 0.08 |
| 1OC0 | 7.4 | -0.05 | -0.01 | 1.05 | 2.26 | 1.01 | 1.32 | 1.55 | 1.35 | 2.34 | 2.35 | 0.64 | 0.64 |
| 1OFU | 5.6 | 0.19 | 0.20 | 0.09 | 0.52 | 0.37 | 0.75 | 2.03 | 2.33 | 4.56 | 11.36 | 0.18 | 0.13 |
| 1OPH | 8.3 | -0.35 | -0.10 | 5.00 | 4.69 | 0.00 | 0.68 | 1.66 | 1.62 | 1.77 | 2.41 | 0.79 | 0.55 |
| 1OYV | 6 | 0.13 | 0.03 | 3.86 | 3.61 | 1.04 | 1.03 | 2.05 | 2.11 | 4.46 | 3.28 | 0.15 | 0.27 |
| 1PPE | 4.5[13] | -0.73 | -0.70 | 5.00 | 5.00 | 0.00 | 0.00 | 1.00 | 0.92 | 1.02 | 0.97 | 0.72 | 0.69 |
| 1PVH | 7.5 | -0.84 | -1.07 | 5.00 | 5.00 | 0.00 | 0.00 | 0.42 | 0.44 | 0.54 | 0.52 | 0.83 | 0.83 |
| 1PXV | 6.3 | 0.04 | 0.02 | 0.00 | 0.00 | 0.00 | 0.00 | 4.58 | 4.49 | 11.91 | 13.05 | 0.04 | 0.04 |
| 1QA9 | 7.5 | -0.19 | -0.11 | 5.00 | 4.98 | 0.00 | 0.15 | 0.78 | 0.73 | 0.99 | 0.97 | 0.73 | 0.77 |
| 1QFW | 8 | -0.11 | -0.45 | 1.88 | 4.48 | 1.14 | 0.86 | 0.84 | 0.93 | 1.08 | 1.04 | 0.66 | 0.87 |
| 1R0R | 8.5 | 0.25 | 0.07 | 0.69 | 1.41 | 0.88 | 1.04 | 1.28 | 1.47 | 7.65 | 7.40 | 0.12 | 0.09 |
| 1R6Q | 8.5[14] | 0.03 | -0.06 | 1.39 | 3.64 | 1.15 | 1.06 | 1.76 | 1.77 | 4.05 | 2.04 | 0.23 | 0.37 |
| 1RV6 | 6.5 | 0.10 | 0.06 | 2.52 | 3.83 | 1.23 | 1.04 | 0.99 | 1.05 | 10.73 | 10.38 | 0.05 | 0.08 |
| 1S1Q | 4.6 | -0.19 | -0.01 | 4.97 | 4.58 | 0.19 | 0.85 | 1.04 | 1.05 | 1.43 | 3.92 | 0.61 | 0.32 |
| 1SBB | 8.5 | -0.25 | 0.08 | 4.78 | 3.03 | 0.53 | 1.24 | 0.73 | 0.53 | 0.73 | 8.92 | 0.74 | 0.07 |
| 1SYX | 6.5 | -0.12 | -0.03 | 4.67 | 4.80 | 0.63 | 0.50 | 1.52 | 1.76 | 2.08 | 2.43 | 0.55 | 0.45 |
| 1T6B | 9 | 0.03 | 0.05 | 1.78 | 2.86 | 1.13 | 1.13 | 1.27 | 1.30 | 5.33 | 5.59 | 0.24 | 0.05 |
| 1TMQ | 8.5[15] | 0.00 | 0.02 | 1.15 | 2.87 | 1.03 | 1.19 | 1.66 | 1.62 | 1.66 | 2.51 | 0.42 | 0.30 |
| 1UDI | 6.8[16] | -0.07 | -0.23 | 4.88 | 5.00 | 0.39 | 0.02 | 1.12 | 1.29 | 3.01 | 3.05 | 0.27 | 0.23 |
| 1US7 | 6 | 0.22 | 0.49 | 0.71 | 0.35 | 1.06 | 0.64 | 0.93 | 1.02 | 11.88 | 9.62 | 0.07 | 0.04 |
| 1VFB | 7.1[17] | 0.04 | 0.19 | 2.86 | 3.43 | 1.20 | 1.06 | 0.67 | 0.61 | 4.05 | 3.28 | 0.27 | 0.18 |
| 1WDW | 5.6 | -0.19 | -0.30 | 5.00 | 4.57 | 0.04 | 0.75 | 1.58 | 1.62 | 2.58 | 1.79 | 0.38 | 0.46 |
| 1WEJ | 6.4 | 0.31 | 0.28 | 1.37 | 0.01 | 1.04 | 0.13 | 0.85 | 0.89 | 6.91 | 6.88 | 0.19 | 0.11 |
| 1XD3 | 8.5 | 0.06 | 0.04 | 3.88 | 2.93 | 0.98 | 1.18 | 1.61 | 1.73 | 3.84 | 3.77 | 0.29 | 0.34 |
| 1XQS | 7.5 | -0.11 | -0.01 | 4.06 | 3.89 | 1.06 | 1.02 | 2.11 | 2.12 | 2.78 | 4.98 | 0.33 | 0.35 |
| 1XU1 | 7.5 | -0.31 | -0.42 | 4.22 | 5.00 | 0.94 | 0.02 | 1.06 | 1.23 | 1.80 | 1.47 | 0.51 | 0.53 |

| | | Discrimination Score | | N5 (Mean) | | N5 (SD) | | Best Irmsd | | Best scored Irmsd | | Best scored $f_{nat}$ | |
|---|---|---|---|---|---|---|---|---|---|---|---|---|---|
| PDB | pH | pHDock | RsDock | pHDock | RsDock | pHDock | RsDock | pHDock | RsDock | pHDock | RsDock | pHDock | RsDock |
| 1Y64 | 7.6 | 0.00 | 0.00 | 0.00 | 0.00 | 0.00 | 0.00 | 14.78 | 15.41 | 14.78 | 26.40 | 0.00 | 0.00 |
| 1YVB | 6 | -0.12 | 0.04 | 4.86 | 5.00 | 0.45 | 0.06 | 0.94 | 0.76 | 1.22 | 2.57 | 0.52 | 0.39 |
| 1Z0K | 6 | 0.51 | 0.33 | 0.00 | 0.34 | 0.00 | 0.63 | 0.69 | 0.75 | 6.24 | 7.79 | 0.35 | 0.31 |
| 1Z5Y | 4.5 | 0.00 | 0.00 | 3.55 | 3.64 | 1.08 | 1.03 | 1.85 | 2.39 | 2.91 | 3.46 | 0.44 | 0.30 |
| 1ZHH | 9.5 | 0.45 | 0.47 | 1.68 | 1.24 | 1.15 | 1.10 | 1.50 | 1.88 | 4.09 | 13.17 | 0.24 | 0.03 |
| 1ZHI | 7.5 | -0.04 | -0.01 | 3.86 | 4.63 | 0.98 | 0.67 | 0.96 | 0.96 | 1.08 | 1.57 | 0.65 | 0.59 |
| 1ZM4 | 7.2 | -0.16 | -0.06 | 4.61 | 3.98 | 0.83 | 0.99 | 2.20 | 2.39 | 2.69 | 2.53 | 0.38 | 0.45 |
| 2A5T | 7 | 0.13 | 0.35 | 1.08 | 0.00 | 0.97 | 0.00 | 1.76 | 1.65 | 4.37 | 13.58 | 0.38 | 0.02 |
| 2A9K | 7 | 0.00 | -0.15 | 3.77 | 3.61 | 0.99 | 1.17 | 1.17 | 1.11 | 3.63 | 1.89 | 0.22 | 0.53 |
| 2ABZ | 8.5 | -0.06 | -0.02 | 4.42 | 4.66 | 0.79 | 0.66 | 1.26 | 1.29 | 3.10 | 3.03 | 0.33 | 0.33 |
| 2AJF | 7.5 | 0.28 | 0.30 | 1.30 | 0.93 | 1.13 | 0.93 | 0.95 | 0.88 | 16.01 | 15.29 | 0.14 | 0.00 |
| 2AYO | 8 | -0.16 | 0.32 | 2.62 | 1.96 | 1.15 | 1.15 | 2.08 | 1.97 | 2.08 | 11.23 | 0.41 | 0.00 |
| 2B42 | 4.6 | -1.19 | 0.16 | 2.69 | 0.00 | 1.29 | 0.00 | 1.66 | 2.86 | 1.66 | 4.71 | 0.49 | 0.14 |
| 2B4J | 6.3 | 0.26 | 0.02 | 3.37 | 1.85 | 1.11 | 1.13 | 2.13 | 2.14 | 4.16 | 3.97 | 0.68 | 0.58 |
| 2BTF | 7* | 0.12 | 0.33 | 3.73 | 1.33 | 1.02 | 1.14 | 1.48 | 1.43 | 3.06 | 7.51 | 0.37 | 0.07 |
| 2C0L | 6.5[18] | 0.05 | 0.02 | 0.00 | 0.00 | 0.02 | 0.00 | 3.86 | 4.13 | 4.04 | 7.07 | 0.21 | 0.07 |
| 2CFH | 7.4[19] | -0.07 | -0.20 | 4.80 | 5.00 | 0.49 | 0.00 | 1.83 | 1.81 | 3.40 | 2.11 | 0.29 | 0.40 |
| 2FD6 | 6.5 | 0.16 | 0.08 | 2.60 | 2.50 | 1.17 | 1.20 | 1.28 | 1.47 | 3.83 | 2.30 | 0.27 | 0.64 |
| 2FJU | 8.5 | 0.16 | 0.26 | 3.17 | 2.43 | 1.34 | 1.27 | 0.84 | 0.88 | 2.94 | 3.30 | 0.59 | 0.41 |
| 2H7V | 6.5 | 0.00 | 0.10 | 1.58 | 2.29 | 1.09 | 1.18 | 2.28 | 2.11 | 2.96 | 4.17 | 0.30 | 0.19 |
| 2HLE | 7.8 | -0.23 | -0.18 | 4.80 | 4.81 | 0.50 | 0.50 | 1.74 | 1.74 | 1.85 | 2.25 | 0.46 | 0.47 |
| 2HMI | 7* | 0.12 | 0.20 | 0.00 | 0.00 | 0.00 | 0.00 | 3.68 | 3.16 | 5.96 | 10.26 | 0.32 | 0.12 |
| 2HQS | 4.6 | -0.18 | 0.38 | 3.63 | 2.57 | 1.04 | 1.28 | 2.01 | 1.70 | 2.01 | 8.05 | 0.46 | 0.00 |
| 2HRK | 7.2 | -0.10 | -0.19 | 2.62 | 4.57 | 1.27 | 0.75 | 1.19 | 1.45 | 1.70 | 1.56 | 0.69 | 0.72 |
| 2I25 | 8.3 | 0.18 | 0.36 | 1.14 | 0.22 | 1.04 | 0.53 | 2.22 | 2.28 | 6.39 | 6.37 | 0.14 | 0.11 |
| 2I9B | 5.5 | 0.00 | 0.02 | 0.00 | 0.00 | 0.00 | 0.00 | 5.11 | 5.34 | 7.19 | 6.00 | 0.15 | 0.32 |
| 2IDO | 8 | 0.07 | 0.06 | 0.00 | 0.01 | 0.00 | 0.12 | 3.48 | 3.47 | 7.56 | 7.61 | 0.11 | 0.08 |
| 2J0T | 7.5 | 0.13 | 0.05 | 0.12 | 3.94 | 0.38 | 1.02 | 2.12 | 2.13 | 5.19 | 2.69 | 0.11 | 0.26 |

| PDB | pH | Discrimination Score | | N5 (Mean) | | N5 (SD) | | Best Irmsd | | Best scored Irmsd | | Best scored $f_{nat}$ | |
|---|---|---|---|---|---|---|---|---|---|---|---|---|---|
| | | pHDock | RsDock | pHDock | RsDock | pHDock | RsDock | pHDock | RsDock | pHDock | RsDock | pHDock | RsDock |
| 2JEL | 5.8 | -0.58 | -0.20 | 5.00 | 2.64 | 0.01 | 1.27 | 0.37 | 0.63 | 0.41 | 1.08 | 0.95 | 0.88 |
| 2MTA | 7* | -0.15 | -0.11 | 5.00 | 5.00 | 0.08 | 0.03 | 0.45 | 0.53 | 1.18 | 1.18 | 0.66 | 0.66 |
| 2NZ8 | 6 | 0.37 | 0.07 | 0.00 | 0.32 | 0.00 | 0.62 | 2.96 | 3.11 | 9.42 | 9.50 | 0.11 | 0.08 |
| 2O3B | 5.5 | 0.07 | 0.11 | 0.95 | 0.01 | 0.94 | 0.10 | 3.16 | 3.23 | 10.58 | 10.65 | 0.07 | 0.07 |
| 2O8V | 4.6 | 0.32 | 0.41 | 0.01 | 0.00 | 0.13 | 0.00 | 1.51 | 1.49 | 8.37 | 9.32 | 0.16 | 0.21 |
| 2OOB | 5 | 0.02 | 0.17 | 2.25 | 2.44 | 1.22 | 1.17 | 1.16 | 1.25 | 7.03 | 7.07 | 0.23 | 0.27 |
| 2OT3 | 6 | 0.05 | 0.12 | 0.00 | 0.00 | 0.00 | 0.00 | 4.75 | 4.95 | 7.26 | 13.57 | 0.09 | 0.03 |
| 2OUL | 7 | -0.99 | -0.94 | 5.00 | 5.00 | 0.00 | 0.00 | 0.65 | 0.64 | 0.69 | 0.65 | 0.78 | 0.82 |
| 2OZA | 6 | 0.02 | -0.02 | 3.57 | 1.99 | 1.10 | 1.21 | 2.89 | 2.83 | 4.06 | 4.12 | 0.15 | 0.26 |
| 2PCC | 7[20] | 0.43 | 0.45 | 1.66 | 1.35 | 1.21 | 1.10 | 0.42 | 0.33 | 7.87 | 3.08 | 0.20 | 0.25 |
| 2QFW | 8 | 0.29 | 0.01 | 1.28 | 3.41 | 1.13 | 1.11 | 1.15 | 1.02 | 14.11 | 11.99 | 0.00 | 0.00 |
| 2SNI | 5.6[21] | -0.22 | -0.30 | 5.00 | 4.89 | 0.00 | 0.43 | 0.86 | 0.91 | 1.19 | 0.94 | 0.66 | 0.66 |
| 2UUY | 6.5[22] | -0.18 | -0.05 | 4.97 | 3.91 | 0.20 | 1.02 | 1.33 | 0.78 | 1.78 | 5.90 | 0.59 | 0.10 |
| 2VDB | 7.5[23] | -0.49 | -0.84 | 5.00 | 5.00 | 0.00 | 0.01 | 0.50 | 0.55 | 0.56 | 0.84 | 0.78 | 0.72 |
| 2VIS | 6[24] | 0.53 | 0.40 | 0.00 | 0.00 | 0.04 | 0.00 | 1.44 | 1.65 | 11.99 | 11.80 | 0.08 | 0.06 |
| 2Z0E | 5.8 | -0.03 | -0.03 | 1.82 | 1.69 | 1.14 | 1.19 | 3.69 | 3.55 | 4.93 | 4.01 | 0.13 | 0.17 |
| 3BP8 | 5.5 | 0.22 | 0.62 | 1.33 | 0.11 | 1.14 | 0.39 | 1.70 | 1.24 | 10.44 | 7.49 | 0.08 | 0.13 |
| 3CPH | 5.5 | 0.17 | 0.16 | 0.08 | 0.00 | 0.35 | 0.00 | 2.80 | 2.79 | 9.15 | 9.21 | 0.07 | 0.02 |
| 3D5S | 7 | -0.29 | -0.96 | 4.55 | 4.99 | 0.76 | 0.14 | 0.62 | 0.64 | 0.62 | 0.64 | 0.82 | 0.74 |
| 3SGQ | 10.7 | -0.20 | 0.13 | 3.34 | 3.45 | 1.31 | 1.11 | 0.72 | 0.85 | 0.98 | 7.89 | 0.76 | 0.11 |
| 4CPA | 7.5[25] | 0.67 | 0.14 | 0.98 | 0.96 | 1.08 | 0.93 | 1.21 | 1.46 | 7.21 | 6.37 | 0.21 | 0.15 |
| 7CEI | 6 | -0.71 | -0.73 | 5.00 | 5.00 | 0.02 | 0.00 | 0.52 | 0.47 | 0.72 | 0.55 | 0.83 | 0.90 |
| BOYV | 6 | -0.06 | -0.53 | 2.11 | 4.25 | 1.21 | 1.12 | 1.24 | 1.24 | 1.24 | 1.42 | 0.59 | 0.63 |

The pH values are extracted from the PDB coordinate files of the bound complexes. For structures missing pH information in the PDB file, we used the pH value from the corresponding original research article if available (citations added). For the remaining structures, we assumed a physiological pH of 7.0 (indicated by *).

**REFERENCES**


1. Frigerio F, Coda A, Pugliese L, Lionetti C, Menegatti E, et al. (1992) Crystal and molecular structure of the bovine α-chymotrypsin-eglin c complex at 2.0 Å resolution. J Mol Biol 225: 107–123. doi:10.1016/0022-2836(92)91029-O.

2. Kabsch W, Mannherz HG, Suck D, Pai EF, Holmes KC (1990) Atomic structure of the actin: DNase I complex. Nature 347: 37–44. doi:10.1038/347037a0.

3. Hecht HJ, Szardenings M, Collins J, Schomburg D (1991) Three-dimensional structure of the complexes between bovine chymotrypsinogen A and two recombinant variants of human pancreatic secretory trypsin inhibitor (Kazal-type). J Mol Biol 220: 711–722. doi:10.1016/0022-2836(91)90112-J.

4. Kobe B, Ma Z, Deisenhofer J (1994) Complex between Bovine Ribonuclease A and Porcine Ribonuclease Inhibitor Crystallizes in a Similar Unit Cell as Free Ribonuclease Inhibitor. J Mol Biol 241: 288–291. doi:10.1006/jmbi.1994.1502.

5. Sauer-Eriksson AE, Kleywegt GJ, Uhlén M, Jones TA (1995) Crystal structure of the C2 fragment of streptococcal protein G in complex with the Fc domain of human IgG. Structure 3: 265–278. doi:10.1016/S0969-2126(01)00157-5.

6. Hurley JH, Faber HR, Worthylake D, Meadow ND, Roseman S, et al. (1993) Structure of the regulatory complex of Escherichia coli IIIGlc with glycerol kinase. Science 259: 673–677. doi:10.1126/science.8430315.

7. Shiba T, Kawasaki M, Takatsu H, Nogi T, Matsugaki N, et al. (2003) Molecular mechanism of membrane recruitment of GGA by ARF in lysosomal protein transport. Nat Struct Mol Biol 10: 386–393. doi:10.1038/nsb920.

8. Hart PJ, Deep S, Taylor AB, Shu Z, Hinck CS, et al. (2002) Crystal structure of the human TβR2 ectodomain–TGF-β3 complex. Nat Struct Mol Biol 9: 203–208. doi:10.1038/nsb766.

9. Marchot P, Ravelli RBG, Raves ML, Bourne Y, Vellom DC, et al. (1996) Soluble monomeric acetylcholinesterase from mouse: Expression, purification, and crystallization in complex with fasciculin. Protein Science 5: 672–679. doi:10.1002/pro.5560050411.

10. Braden BC, Souchon H, Eiselé J-L, Bentley GA, Bhat TN, et al. (1994) Three-dimensional structures of the free and the antigen-complexed Fab from monoclonal anti-lysozyme antibody D44.1. J Mol Biol 243: 767–781. doi:10.1016/0022-2836(94)90046-9.

11. Tulip WR, Varghese JN, Laver WG, Webster RG, Colman PM (1992) Refined crystal structure of the influenza virus N9 neuraminidase-NC41 Fab complex. J Mol Biol 227: 122–148. doi:10.1016/0022-2836(92)90687-F.

12. Bossart-Whitaker P, Chang CY, Novotny J, Benjamin DC, Sheriff S (1995) The Crystal Structure of the Antibody N10-Staphylococcal Nuclease Complex at 2.9 Å Resolution. J Mol Biol 253: 559–575. doi:10.1006/jmbi.1995.0573.

13. Bode W, Greyling HJ, Huber R, Otlewski J, Wilusz T (1989) The refined 2.0 Å X-ray crystal structure of the complex formed between bovine β-trypsin and CMTI-I, a trypsin inhibitor from squash seeds (Cucurbita maxima) Topological similarity of the squash seed inhibitors with the carboxypeptidase A inhibitor from potatoes. FEBS Lett 242: 285–292. doi:10.1016/0014-5793(89)80486-7.

14. Xia D, Esser L, Singh SK, Guo F, Maurizi MR (2004) Crystallographic investigation of peptide binding sites in the N-domain of the ClpA chaperone. J Struct Biol 146: 166–179. doi:10.1016/j.jsb.2003.11.025.

15. Strobl S, Maskos K, Wiegand G, Huber R, Gomis-Rüth FX, et al. (1998) A novel strategy for inhibition of α-amylases: yellow meal worm α-amylase in complex with the Ragi bifunctional inhibitor at 2.5 å resolution. Structure 6: 911–921. doi:10.1016/S0969-2126(98)00092-6.

16. Savva R, Pearl LH (1993) Crystallization and Preliminary X-ray Analysis of the Uracil-DNA Glycosylase DNA Repair Enzyme from Herpes Simplex Virus Type 1. J Mol Biol 234: 910–912. doi:10.1006/jmbi.1993.1642.

17. Bhat TN, Bentley GA, Boulot G, Greene MI, Tello D, et al. (1994) Bound water molecules and conformational stabilization help mediate an antigen-antibody association. Proc Natl Acad Sci USA 91: 1089–1093. doi:10.1073/pnas.91.3.1089.

18. Stanley WA, Filipp FV, Kursula P, Schüller N, Erdmann R, et al. (2006) Recognition of a Functional Peroxisome Type 1 Target by the Dynamic Import Receptor Pex5p. Molecular Cell 24: 653–663. doi:10.1016/j.molcel.2006.10.024.

19. Kümmel D, Müller JJ, Roske Y, Henke N, Heinemann U (2006) Structure of the Bet3–Tpc6B Core of TRAPP: Two Tpc6 Paralogs Form Trimeric Complexes with Bet3 and Mum2. J Mol Biol 361: 22–32. doi:10.1016/j.jmb.2006.06.012.

20. Pelletier H, Kraut J (1992) Crystal structure of a complex between electron transfer partners, cytochrome c peroxidase and cytochrome c. Science 258: 1748–1755. doi:10.1126/science.1334573.

21. McPhalen CA, Schnebli HP, James MNG (1985) Crystal and molecular structure of the inhibitor eglin from leeches in complex with subtilisin Carlsberg. FEBS Lett 188: 55–58. doi:10.1016/0014-5793(85)80873-5.

22. Paesen GC, Siebold C, Harlos K, Peacey MF, Nuttall PA, et al. (2007) A Tick Protein with a Modified Kunitz Fold Inhibits Human Tryptase. J Mol Biol 368: 1172–1186. doi:10.1016/j.jmb.2007.03.011.

23. Lejon S, Cramer JF, Nordberg P (2008) Structural basis for the binding of naproxen to human serum albumin in the presence of fatty acids and the GA module. Acta Cryst 64: 64–69. doi:10.1107/S174430910706770X.

24. Fleury D, Wharton SA, Skehel JJ, Knossow M, Bizebard T (1998) Antigen distortion allows influenza virus to escape neutralization. Nat Struct Mol Biol 5: 119–123. doi:10.1038/nsb0298-119.

25. Rees DC, Lipscomb WN (1982) Refined crystal structure of the potato inhibitor complex of carboxypeptidase A at 2.5 Å resolution. J Mol Biol 160: 475–498. doi:10.1016/0022-2836(82)90309-6.
