# Supplementary material for: Protein-Protein Docking with Dynamic Residue Protonation States
Source: PLoS Comput Biol. 2014 Dec 11;10(12):e1004018. doi: 10.1371/journal.pcbi.1004018 (PMC4263365; doi:10.1371/journal.pcbi.1004018)
Supplement: S3 Table — Ensemble pHDock performance summary. PDB IDs and pH values of the benchmark subset used for ensemble pHDock. Discrimination scores, Irmsd, f nat, and number of recovered native interface hydrogen bonds in the top-ranked models generated using ensemble pHDock are compared to pHDock and RosettaDock. (PDF) [file pcbi.1004018.s014.pdf]

Table S3. Ensemble pHDock performance summary. PDB IDs and pH values of the benchmark subset used for ensemble pHDock. Discrimination scores, Irmsd, f_{nat}, and number of recovered native interface hydrogen bonds in the top-ranked models generated using ensemble pHDock are compared to pHDock and RosettaDock.

		Discrimination score			Best scored Irmsd			Best scored f_{nat}			Recovered native interface hydrogen bonds			
**PDB**	**pH**	**RsDock**	**pHDock**	**Ens pHDock**	**RsDock**	**pHDock**	**Ens pHDock**	**RsDock**	**pHDock**	**Ens pHDock**	**Total**	**RsDock**	**pHDock**	**Ens pHDock**
1ACB	6.5	0.01	-0.24	0.46	3.15	2.37	4.32	0.22	0.21	0.19	7	1	0	1
1AY7	7	-0.17	-0.17	-0.67	1.32	0.63	0.72	0.74	0.74	0.82	9	2	6	6
1B6C	8.5	-0.34	-0.36	-0.58	1.80	1.90	1.50	0.61	0.71	0.78	2	0	0	0
1BVK	6.5	0.11	0.24	0.05	8.92	5.70	1.97	0.06	0.18	0.61	8	1	1	1
1CGI	8.5	0.04	0.03	0.01	3.59	9.65	2.60	0.24	0.06	0.42	8	0	0	1
1DFJ	5	-0.71	-0.17	-0.50	1.48	2.14	1.96	0.62	0.40	0.60	4	0	0	0
1EAW	8	0.00	-0.06	-0.10	3.73	3.55	1.51	0.15	0.15	0.50	11	0	1	2
1KTZ	4.5	-0.52	-0.35	-0.38	0.51	0.42	1.25	0.93	0.93	0.71	6	2	3	3
1MAH	7	-0.13	-0.16	-0.23	2.32	1.52	1.80	0.38	0.52	0.44	5	1	0	0
1MLC	6	0.13	-0.11	-0.07	2.59	1.12	2.44	0.37	0.71	0.51	6	0	0	0
2BTF	7	0.33	0.12	-0.10	7.51	3.06	2.11	0.07	0.37	0.43	7	0	0	1
2JEL	5.8	-0.20	-0.58	-0.74	1.08	0.41	0.68	0.88	0.95	0.93	7	3	3	4
2PCC	7	0.45	0.43	0.40	3.08	7.87	3.83	0.25	0.20	0.50	1	0	0	0
2SNI	5.6	-0.30	-0.22	-0.19	0.94	1.19	1.50	0.66	0.66	0.63	8	4	2	4
