# Supplementary material for: Protein-Protein Docking with Dynamic Residue Protonation States
Source: PLoS Comput Biol. 2014 Dec 11;10(12):e1004018. doi: 10.1371/journal.pcbi.1004018 (PMC4263365; doi:10.1371/journal.pcbi.1004018)
Supplement: S1 Text — Command lines for pHDock, RosettaDock and ensemble pHDock. (PDF) [file pcbi.1004018.s015.pdf]

Text S1. Command lines for pHDock, RosettaDock and ensemble pHDock.

The algorithms in the paper are implemented using the Rosetta molecular modeling suite which is free for academic and non-profit use and downloadable from www.rosettacommons.org. All algorithms and supporting scripts are distributed in the Rosetta release. The Rosetta command-line arguments used for the calculations are as follows:

Pre-packing stage

(a) pHDock

```
docking_prepack_protocol.<exe>
      -s 1A2K.u.pdb -native 1A2K.b.pdb
      -partners AB_C
      -dock_ppk
      -pH:pH_mode -pH:value_pH 5.6
      -core:weights pH_pack.wts
      -ex1 -ex2aro
      -unboundrot 1A2K.u.pdb
```

where the unbound complex `1A2K.u.pdb` is pre-packed at pH `5.6`. The `-partners` argument identifies the receptor and ligand chains and `-native` provides the bound complex `1A2K.b.pdb` for comparison.

(b) RosettaDock

```
docking_prepack_protocol.<exe>
      -s 1A2K.u.pdb -native 1A2K.b.pdb
      -partners AB_C
      -dock_ppk
      -ex1 -ex2aro
      -unboundrot 1A2K.u.pdb
```

(c) Ensemble pHDock

```
docking_prepack_protocol.<exe>
      -s 1ACB.u.pdb -native 1ACB.b.pdb
      -partners A_B
      -ensemble1 pdblist1 -ensemble2 pdblist2
      -dock_ppk
      -pH:pH_mode -pH:value_pH 6.5
      -core:weights pH_pack.wts
      -ex1 -ex2aro
      -unboundrot 1ACB.u.pdb
      -out:nooutput
```

where `pdblist1` and `pdblist2` are the list of coordinate files for receptor and ligand backbone ensembles.

Docking stage

(a) pHDock

```
docking_protocol.<exe>
      -s 1A2K.u.ppk.pdb -native 1A2K.b.pdb
      -partners AB_C
      -dock_pert 3 8 -spin
      -pH:pH_mode -pH:value_pH 5.6
      -pack_patch pH_pack -high_patch pH_dock -high_min_patch pH_min
      -ex1 -ex2aro
      -unboundrot 1A2K.u.pdb
      -nstruct 1000
```

where the unbound pre-packed complex `1A2K.u.ppk.pdb` is docked at pH `5.6`. The new score term weights are implemented using the three `-patch` arguments.

(b) RosettaDock

```
docking_protocol.<exe> -database <path_to_rosetta_database>
      -s 1A2K.u.ppk.pdb -native 1A2K.b.pdb
      -partners AB_C
      -dock_pert 3 8 -spin
      -ex1 -ex2aro
      -unboundrot 1A2K.u.pdb
      -nstruct 1000
```

(c) Ensemble pHDock

```
docking_protocol.<exe>
      -s 1ACB.u.ppk.pdb -native 1ACB.b.pdb
      -partners A_B
      -ensemble1 pdblist1 -ensemble2 pdblist2
      -dock_pert 3 8 -spin
      -pH:pH_mode -pH:value_pH 6.5
      -pack_patch pH_pack -high_patch pH_dock -high_min_patch pH_min
      -ex1 -ex2aro
      -unboundrot 1A2K.u.pdb
      -nstruct 1000
```

(d) Fix pHDock

```
docking_protocol.<exe>
      -s 1ACB.u.ppk.pdb -native 1ACB.b.pdb
      -partners A_B
      -dock_pert 3 8 -spin
      -pH:pH_mode -pH:value_pH 6.5
      -pH:keep_input_protonation_state
      -pH:fix_protonation_states
      -pack_patch pH_pack -high_patch pH_dock -high_min_patch pH_min
      -ex1 -ex2aro
      -unboundrot 1A2K.u.pdb
      -nstruct 1000
```

Backbone ensemble generation

(a) RosettaRelax

```
relax.<exe>
      -s 1ACB.l.pdb
      -ex1 -ex2aro
      -nstruct 500
```

where `1ACB.l.pdb` is the unbound ligand.
